# Proceedings of The 8th Romanian National HIV/AIDS Congress and The 3rd Central European HIV Forum: Sibiu, Romania. 5-7 May 2016

**DOI:** 10.1186/s12879-016-1480-8

**Published:** 2016-06-27

**Authors:** Ivailo Alexiev, Reneta Dimitrova, Anna Gancheva, Asya Kostadinova, Mariyana Stoycheva, Daniela Nikolova, Ivaylo Elenkov, Cătălin Tilișcan, Mioara Predescu, Bogdan Păunescu, Anca Streinu-Cercel, Oana Săndulescu, Claudiu Mihai Șchiopu, Mădălina Hristache, Lăcrămioara Aurelia Brîndușe, Adrian Streinu-Cercel, Marija Todorovic, Marina Siljic, Dubravka Salemovic, Valentina Nikolic, Ivana Pesic-Pavlovic, Jovan Ranin, Djordje Jevtovic, Maja Stanojevic, Ana Maria Tudor, Delia Vlad, Mariana Mărdărescu, Sorin Petrea, Cristina Petre, Ruxandra Neagu-Drăghicenoiu, Rodica Ungurianu, Alina Cibea, Odette Chirilă, Cristian Anghelina, Ileana Coserea, Pantelia-Amalia Krikelli, Eirini Pavlitina, Mina Psichogiou, Demetris Lamnisos, Leslie Williams, Anya Korobchuk, Britt Skaathun, Pavlo Smyrnov, John Schneider, Vana Sypsa, Dimitrios Paraskevis, Angelos Hatzakis, Samuel R. Friedman, Georgios K. Nikolopoulos, Gordana Dragović, Danica Srdić, Al Musalhi Khawla, Ivan Soldatović, Jelena Nikolić, Djordje Jevtović, Devaki Nair, Aura Temereanca, Adelina Rosca, Luminita Ene, Benchawa Soontornniyomkij, Carmen Diaconu, Claudia Dita, Cristian Achim, Simona Ruta, Șerban Benea, Ruxandra Moroti, Raluca Jipa, Eliza Manea, Andrada Stan, Elisabeta Benea, Dan Oțelea, Adriana Hristea, Adriana Hristea, Irina Lăpădat, Raluca Jipa, Ruxandra Moroti, Șerban Benea, Doina Antonică, Irina Panait, Roxana Petre, Justyna D. Kowalska, Ewa Pietraszkiewicz, Ewa Grycner, Ewa Firlag-Burkacka, Andrzej Horban, Ovidiu Vlaicu, Leontina Bănică, Simona Paraschiv, Ana-Maria Tudor, Ruxandra Moroti, Dan Oțelea, Bojana Dimitrijević, Ivan Soldatović, Đorđe Jevtović, Jovana Kusić, Dubravka Salemović, Jovan Ranin, Gordana Dragović, Dragoș Florea, Ioana Bădicuț, Alexandru Rafila, Cornel Camburu, Adriana Histrea, Mihaela Frățilă, Dan Oțelea, Ivana Gmizic, Dubravka Salemovic, Ivana Pesic-Pavlovic, Marina Siljic, Valentina Nikolic, Miljana Djonin-Nenezic, Ivana Milosevic, Branko Brmbolic, Maja Stanojevic, Anca Streinu-Cercel, Oana Săndulescu, Alina Cristina Neguț, Mioara Predescu, Alexandra Mărdărescu, Mihai Săndulescu, Adrian Streinu-Cercel, Ana Belen Pérez, Natalia Chueca, Marta Álvarez, Juan Carlos Alados, Antonio Rivero, Francisco Vera, Marcial Delgado, Javier Salmeron, Miguel Jiménez, Maria José Blanco, Moises Diago, Miguel Garcia-deltoro, Marta Alvarez, Francisco Téllez, Federico García, Diana Tănase, Eliza Manea, Rodica Bacruban, Dragoș Florea, Dan Oțelea, Alexandru Rafila, Mariana Mărdărescu, Adriana Hristea, Ivana Grgic, Ana Planinic, Maja Santak, Lana Gorenec, Snjezana Zidovec Lepej, Adriana Vince, Eliza Manea, Adriana Hristea, Șerban Benea, Ruxandra Moroti, Diana Tănase, Cristian M. Niculae, Simona Merisor, Raluca Jipa, Dimitrios Paraskevis, Evangelia Kostaki, Georgios K. Nikolopoulos, Vana Sypsa, Mina Psichogiou, Dimitra Paraskeva, Athanassios Skoutelis, Meni Malliori, Samuel R. Friedman, Angelos Hatzakis, Malgorzata Hackiewicz, Piotr Zabek, Ewa Firlag-Burkacka, Andrzej Horban, Justyna Dominika Kowalska, Maja M. Lunar, Jana Mlakar, Mario Poljak, Leontina Bănică, Eliza Martin, Valeriu Gheorghiță, Andrei Petrescu, Dan Oțelea, Costin-Ioan Popescu, Simona Paraschiv, Emil Neaga, Vlaicu Ovidiu, Andrei Juncu, Leontina Bănică, Simona Paraschiv, Dan Oțelea, Costin-Ioan Popescu, Adrian Luca, Florin Lazăr, Anca Elena Luca, Luminița Ene, Cristian Achim, Cosmina Gingăraş, Ștefan Adrian Anton, Roxana Rădoi, Simona Tetradov, Grațiela Țârdei, Maria Nica, Razvan Alexandru Capşa, Cristian L. Achim, Cristiana Oprea, Luminița Ene, Bogna Szymańska, Natalia Gawron, Agnieszka Pluta, Emilia Łojek, Ewa Firląg-Burkacka, Andrzej Horban, Robert Bornstein, et HARMONIA3 Study Group, Olivia Burcoș, Simona Manuela Erscoiu, Filofteia Bănicioiu Cojanu, Andreea Toderan, Maria Nica, Ionuț Cristian Popa, Emanoil Ceaușu, Petre Iacob Calistru, Manuela Arbune, Mirela Alexandrache, Anca-Adriana Arbune, Doina-Carina Voinescu, Ioan-Alexandru Diaconu, Laurențiu Stratan, Victoria Aramă, Luciana Nichita, Alexandra Diaconu, Anca Negru, Alina Orfanu, Anca Leuștean, Daniela Adriana Ion, Irina Ianache, Cristiana Oprea, Anca Leuștean, Cristina Popescu, Alina Orfanu, Anca Negru, Remulus Catana, Cristina Murariu, Ioan-Alexandru Diaconu, Mihaela Rădulescu, Cătălin Tilișcan, Victoria Aramă, Iosif Marincu, Patricia Poptelecan, Valeria Bică, Florin Lazăr, Livius Tirnea, Irina Ianache, Roxana Rădoi, Manuela Nica, Grațiela Țârdei, Luminița Ene, Emanoil Ceaușu, Petre Calistru, Cristiana Oprea, Iurie Osoianu, Ala Halacu, Andreea Cristina Stoian, Florentina Dumitrescu, Iulian Diaconescu, Augustin Cupșa, Lucian Giubelan, Loredana Ionescu, Irina Niculescu, Carmen Chiriac, Nina Șincu, Iringo Zaharia Kezdi, Anca Georgescu, Brândușa Țilea, Cristina Girbovan, Andrea Incze, Andrea Fodor, Alina Cibea, Mariana Mărdărescu, Cristina Petre, Ruxandra Drăghicenoiu, Rodica Ungurianu, Ana Maria Tudor, Delia Vlad, Carina Matei, Elena Dumea, Lucian Cristian Petcu, Simona Claudia Cambrea, Florentina Dumitrescu, Augustin Cupsa, Andreea Cristina Stoian, Lucian Giubelan, Irina Niculescu, Iulian Diaconescu, Dan Hurezeanu, Livia Dragonu, Mioara Cotulbea, Simona Manuela Erscoiu, Ionuț Cristian Popa, Denisa Stroie, Petronela Ionescu, Nedeea Duță, Camelia Dobrea, Irina Voican, Emanoil Ceaușu, Petre Iacob Calistru, Florin Lazăr, Lucian Giubelan, Augustin Cupșa, Iulian Diaconescu, Florentina Dumitrescu, Dan Hurezeanu, Livia Dragonu, Irina Niculescu, Andreea Cristina Stoian, Oana Obretin, Mariana Stănescu, Mihai Jianu, Lana Gorenec, Snjezana Zidovec Lepej, Ivana Grgic, Ana Planinic, Janja Iscic Bes, Adriana Vince, Josip Begovac, Luminița Elena Horga, Corina Itu, Luminița Elena Horga, Laura Augusta David-Aldea, Anca Ciorogar, Cristian Jianu, Mihaela Lupșe, Iuliana Caramangiu, Ovidiu Roșca, Monica Cialma, Andreea Ardeleanu, Iosif Marincu, Raluca Jipa, Eliza Manea, Șerban Benea, Irina Lăpădat, Nicoleta Irimescu, Irina Panait, Cristian Niculae, Adriana Hristea, Jovana Kusic, Djordje Jevtovic, Dubravka Salemovic, Jovan Ranin, Bozana Dimitrijevic, Gordana Dragovic, Laura-Augusta Aldea-David, Carmen Manciuc, Cristina Nicolau, Liviu Prisăcariu, Alexandra Largu, Mariana Mărdărescu, Adrian Streinu-Cercel, Cristina Petre, Marieta Iancu, Sanda Vintilă, Daniela Vitelaru, Iosif Ionel, Claudiu Mihai Șchiopu, Alexandra-Henriette Mărdărescu, Pavel Micsanschi, Tiberiu Holban, Ina Bîstrițchi, Lucia Pârțână, Angela Nagîț, Svetlana Popovici, Maria Talmaci, Irina Cucerova, Sorina Georgiana Mitrescu, Dana Mihalcea, Iulia Caramangiu, Ovidiu Roșca, Iosif Maricu, Anca Negru, Daniela Munteanu, Victoria Aramă, Raluca Mihăilescu, Ioan Diaconu, Remulus Catana, Cristina Popescu, Alina Orfanu, Anca Leuștean, Mihaela Rădulescu, Cătălin Tilișcan, Raluca Năstase, Violeta Molagic, Irina Duport, Cristina Dragomirescu, Ștefan Sorin Aramă, Nicoleta M. Negruț, Violeta Elena Niță, Daniela Ioana Munteanu, Raluca Mihăilescu, Ioan Diaconu, Anca Negru, Cristina Popescu, Victoria Aramă, Alina Orfanu, Cristina Popescu, Anca Leuștean, Anca Negru, Remulus Catana, Ioan Diaconu, Cătălin Tilișcan, Victoria Aramă, Sorin Ștefan Aramă, Ivana Pesic Pavlovia, Dubravka Salemovic, Jovan Ranin, Djordje Jevtovic, Ovidiu Roșca, Andreea Ardeleanu, Iulia Caramangiu, Daniela Desaga, Valerica Bică, Sorina Mitrescu, Iosif Marincu, Marina Siljic, Dubravka Salemovic, Valentina Nikolic, Djordje Jevtovic, Ivana Pesic-Pavlovic, Jovan Ranin, Marija Todorovic, Maja Stanojevic, Nina-Ioana Șincu, Anca Georgescu, Brândușa Țilea, Iringo Zaharia Kezdi, Andrea Incze, Cristina Gârbovan, Carmen Lucia Chiriac, Anca Elena Luca, Florin Lazăr, Adrian Luca, Luminița Ene, Roxana Rădoi, Adina Talnariu, Silvia Suciu, Cristian Achim, Diana Gabriela Iacob, Dragoș Florea, Simona Iacob, Manuela Arbune, Miruna Drăgănescu, Alina Iancu, Ruxandra Moroti, Cristian M. Niculae, Simona Merisor, Eliza Manea, Serban Benea, Andrada Stan, Raluca Hrisca, Raluca Jipa, Diana Tanase, Adriana Hristea, Ivana Grgic, Ana Planinic, Lana Gorenec, Snjezana Zidovec Lepej, Adriana Vince

**Affiliations:** National HIV Reference Laboratory, National Center of Infectious and Parasitic Diseases, Sofia, Bulgaria; Department of Infectious Diseases, Medical University, Plovdiv, Bulgaria; Clinic of Infectious Diseases, Medical University, Varna, Bulgaria; Hospital for Infectious and Parasitic Diseases, Sofia, Bulgaria; National Institute for Infectious Diseases “Prof. Dr. Matei Balș”, Bucharest, Romania; Carol Davila University of Medicine and Pharmacy, Bucharest, Romania; Totem Communications, Bucharest, Romania; National Institute of Public Health, Bucharest, Romania; University of Belgrade Faculty of Medicine, Belgrade, Serbia; Infectious and Tropical Diseases University Hospital, Clinical Center Serbia, HIV/AIDS Unit, Belgrade, Serbia; Virology Department, Clinical Center Serbia, Belgrade, Serbia; National Institute for Infectious Diseases “Prof. Dr. Matei Balș”, Bucharest, Romania; Carol Davila University of Medicine and Pharmacy, Bucharest, Romania; European University of Cyprus, Nicosia, Cyprus; Transmission Reduction Intervention Project, Athens, Greece; Laikon General Hospital, Department of Propedeutic Medicine, Athens, Greece; National Development and Research Institutes, New York, NY USA; Alliance for Public Health, Kyiv, Ukraine; University of Chicago Medical Center, Center for AIDS Elimination, Chicago, IL USA; Medical School, University of Athens, Athens, Greece; Hellenic Centre for Disease Control and Prevention, Amarousio, Greece; Department of Pharmacology, Clinical Pharmacology and Toxicology, School of Medicine, University of Belgrade, Belgrade, Serbia; Department of Clinical Biochemistry, Royal Free Hospital and University College London, London, UK; Institute for Biomedical Statistics, School of Medicine, University of Belgrade, Belgrade, Serbia; Infectious and Tropical Diseases Hospital, School of Medicine, University of Belgrade, Belgrade, Serbia; Carol Davila University of Medicine and Pharmacy, Bucharest, Romania; “Stefan S. Nicolau” Institute of Virology, Bucharest, Romania; “Dr Victor Babes” Hospital for Infectious and Tropical Diseases, Bucharest, Romania; University of California San Diego, La Jolla, San Diego, CA USA; National Institute for Infectious Diseases “Prof Dr Matei Balș”, Bucharest, Romania; Carol Davila University of Medicine and Pharmacy, Bucharest, Romania; National Institute for Infectious Diseases “Prof Dr Matei Balș”, Bucharest, Romania; Carol Davila University of Medicine and Pharmacy, Bucharest, Romania; National Institute of Aerospace Medicine, Bucharest, Romania; Hospital for Infectious Diseases, HIV Out-Patient Clinic, Warsaw, Poland; Department for Adult’s Infectious Diseases, Medical University of Warsaw, Warsaw, Poland; Molecular Diagnostic Laboratory, National Institute for Infectious Diseases “Prof. Dr. Matei Balș”, Bucharest, Romania; Pediatric Department, National Institute for Infectious Diseases “Prof. Dr. Matei Balș”, Bucharest, Romania; Clinical Department III, National Institute for Infectious Diseases “Prof. Dr. Matei Balș”, Bucharest, Romania; Department of Pharmacology, Clinical Pharmacology and Toxicology, School of Medicine, University of Belgrade, Belgrade, Serbia; Institute for Biomedical Statistics, School of Medicine, University of Belgrade, Belgrade, Serbia; Infectious and Tropical Diseases Hospital, School of Medicine, University of Belgrade, Belgrade, Serbia; National Institute for Infectious Diseases “Prof. Dr. Matei Balș”, Bucharest, Romania; Carol Davila University of Medicine and Pharmacy, Bucharest, Romania; Clinic for Infectious and Tropical Diseases, Clinical Centre of Serbia, Belgrade, Serbia; Faculty of Medicine, University of Belgrade, Belgrade, Serbia; Carol Davila University of Medicine and Pharmacy, Bucharest, Romania; National Institute for Infectious Diseases “Prof. Dr. Matei Balș”, Bucharest, Romania; Romanian HIV Center, Bucharest, Romania; Servicio de Microbiologia. CHUG, Hospital San Cecilio, Instituto de investigacion IBS, Granada, Spain; Servicio de Microbiologia. Hospital de Jerez de la Frontera, Cádiz, Spain; Servicio de Enfermedades Infecciosas, Hospital Reina Sofia, Cordoba, Spain; Servicio de Enfermedades Infecciosas, Hospital Santa Lucia, Cartagena, Spain; Servicio de Enfermedades Infecciosas, Hospital Regional Carlos Haya, Malaga, Spain; UGC Digestivo, HUSC, Granada, Spain; UGC Digestivo, Hospital Regional Carlos Haya, Malaga, Spain; UGC Enfermedades Digestivas, Hospital de Jerez de la Frontera, Cádiz, Spain; Servicio de Ap. Digestivo, H Arnau de Vilanova, Valencia, Spain; Servicio de Ap. Digestivo, H General de Valencia, Valencia, Spain; Unidad de Enfermedades Infecciosas, H General de Valencia, Valencia, Spain; Servicio de Ap. Digestivo, H de Poniente, El Ejido, Almeria, Spain; Servicio de Medicina Interna, Hospital de la Linea, Cadiz, Spain; National Institute for Infectious Diseases “Prof. Dr. Matei Balș”, Bucharest, Romania; Department for Molecular Diagnostic And Flow Cytometry, University Hospital for Infectious Diseases Zagreb, Zagreb, Croatia; Department of Viral Hepatitis, University Hospital for Infectious Diseases Zagreb, Zagreb, Croatia; Centre for Research and Knowledge Transfer in Biotechnology, Laboratory for Molecular Biomedicine, University of Zagreb, Zagreb, Croatia; National Institute for Infectious Diseases “Prof Dr Matei Balș”, Bucharest, Romania; Carol Davila University of Medicine and Pharmacy, Bucharest, Romania; Department of Hygiene, Epidemiology and Medical Statistics, Medical School, University of Athens, Athens, Greece; Hellenic Center for Disease Control and Prevention, Amarousio, Greece; Department of Propedeutic Medicine, Laikon General Hospital, Athens, Greece; Fifth Department of Internal Medicine and Infectious Diseases, Evangelismos Hospital Athens, Athina, Greece; Medical School, University of Athens, Athens, Greece; National Development and Research Institutes (NDRI), New York, USA; Department for Adults Infectious Diseases, Medical University of Warsaw, Warsaw, Poland; HIV Out-Patient Clinic, Hospital for Infectious Diseases, Warsaw, Poland; Institute of Microbiology and Immunology, Faculty of Medicine, University of Ljubljana, Ljubljana, Slovenia; Molecular Diagnostic Laboratory, National Institute for Infectious Diseases “Prof. Dr. Matei Balș”, Bucharest, Romania; Institute of Biochemistry of the Romanian Academy, Bucharest, Romania; Clinical Department, National Institute for Infectious Diseases “Prof. Dr. Matei Balș”, Bucharest, Romania; National Institute of Infectious Disease “Prof. Dr. Matei Balș”, Bucharest, Romania; Institute of Biochemistry of the Romanian Academy, Bucharest, Romania; Faculty of Psychology and Education Sciences, University of Bucharest, Bucharest, Romania; Faculty of Sociology and Social Work, University of Bucharest, Bucharest, Romania; “Dr. Victor Babeș” Hospital for Infectious and Tropical Diseases, Bucharest, Romania; HIV Neurobehavioral Research Center, University of California, San Diego, USA; 5th Clinical Department of Infectious Diseases (HIV Department), Dr. Victor Babeș Hospital for Infectious and Tropical Diseases, Bucharest, Romania; Virology, Immunology, Molecular Biology Department, Dr. Victor Babeș Hospital for Infectious and Tropical Diseases, Bucharest, Romania; Microbiology Department, Dr. Victor Babeș Hospital for Infectious and Tropical Diseases, Bucharest, Romania; Dr. Victor Babeș Medical Center for Diagnosis and Treatment, Bucharest, Romania; Department of Radiology, Fundeni Institute, Bucharest, Romania; Departments of Psychiatry and Pathology, UC San Diego, La Jolla, CA USA; Carol Davila University of Medicine and Pharmacy, Bucharest, Romania; Outpatients Clinic, Hospital for Infectious Diseases, Warsaw, Poland; The Faculty of Psychology, University of Warsaw, Warsaw, Poland; World Hearing Center, Institute of Physiology and Pathology of Hearing, Nadarzyn, Poland; Medical University of Warsaw, Warsaw, Poland; Department of Psychiatry and Behavioral Health, Ohio State University, Columbus, OH USA; Clinical Hospital for Infectious and Tropical Diseases “Dr. Victor Babeș”, Bucharest, Romania; Carol Davila University of Medicine and Pharmacy, Bucharest, Romania; Clinical Department, ‘Dunărea de Jos’ University Galați, Galați, Romania; Infectious Diseases Clinical Hospital Iași, Iași, Romania; Clinical Department 6, Carol Davila University of Medicine and Pharmacy, Bucharest, Romania; National Institute for Infectious Diseases “Prof. Dr. Matei Balș”, Bucharest, Romania; Carol Davila University of Medicine and Pharmacy, Bucharest, Romania; Colentina Clinical Hospital, Bucharest, Romania; “Marius Nasta” Pneumophtisiology Institute, Bucharest, Romania; 5th Clinical Department for Infectious Diseases (HIV Department), “Victor Babeș” Hospital for Infectious and Tropical Diseases, Bucharest, Romania; Carol Davila University of Medicine and Pharmacy, Bucharest, Romania; National Institute for Infectious Diseases “Prof. Dr. Matei Balș”, Bucharest, Romania; Carol Davila University of Medicine and Pharmacy, Bucharest, Romania; Department of Infectious Diseases, Pneumology, Epidemiology and Parasitology, “Victor Babeș” University of Medicine and Pharmacy, Timișoara, Romania; Faculty of Sociology and Social Work, University of Bucharest, Bucharest, Romania; 5th Clinical Department for Infectious Diseases (HIV Department), “Dr Victor Babeș” Hospital for Infectious and Tropical Diseases, Bucharest, Romania; Laboratory of Microbiology, “Dr Victor Babeș” Hospital for Infectious and Tropical Diseases, Bucharest, Romania; Laboratory of Virology, Immunology, Molecular Biology, “Dr Victor Babeș” Hospital for Infectious and Tropical Diseases, Bucharest, Romania; Carol Davila University of Medicine and Pharmacy, Bucharest, Romania; National Center of Public Health, Chișinău, Republic of Moldova; Department of Infectious Diseases, University of Medicine and Pharmacy from Craiova, Craiova, Romania; Clinical Hospital of Infectious Diseases from Craiova, Craiova, Romania; Department of Infectious Diseases, University of Medicine and Pharmacy Tîrgu-Mureș, Târgu Mureș, Romania; Department of Laboratory Medicine, University of Medicine and Pharmacy Tîrgu-Mureș, Târgu Mureș, Romania; National Institute for Infectious Diseases “Prof. Dr. Matei Balș”, Bucharest, Romania; Carol Davila University of Medicine and Pharmacy, Bucharest, Romania; Ovidius University, Constanța, Romania; Infectious Diseases Clinical Hospital, Constanța, Romania; Infectious Diseases Department, University of Medicine and Pharmacy from Craiova, Craiova, Romania; “Victor Babeș” Hospital of Infectious Diseases and Pneumology, Craiova, Romania; Carol Davila University of General Medicine and Pharmacy Bucharest, Bucharest, Romania; Clinical Hospital for Infectious and Tropical Diseases Dr. Victor Babeș Bucharest, Bucharest, Romania; Monza Hospital, Bucharest, Romania; Bucharest University Emergency Hospital, Bucharest, Romania; Bucharest University, Bucharest, Romania; University of Medicine and Pharmacy, Craiova, Romania; Victor Babeș Hospital of Infectious Diseases and Pneumology, Craiova, Romania; Department of Molecular Diagnostics and Flow Cytometry, University Hospital for Infectious Diseases Zagreb, Zagreb, Croatia; Department of Viral Hepatitis University Hospital for Infectious Diseases Zagreb, and University of Zagreb, School of Medicine, Zagreb, Croatia; Department of HIV/AIDS, University Hospital for Infectious Diseases Zagreb, and University of Zagreb, School of Medicine, Zagreb, Croatia; Infectious Diseases Hospital Cluj-Napoca, Cluj-Napoca, Romania; Clinical Hospital for Infectious Diseases, Cluj-Napoca, Romania; Iuliu Hațieganu University of Medicine and Pharmacy, Cluj-Napoca, Romania; Department of Infection Diseases I, “Victor Babeș” Clinical Hospital of Infectious Diseases Timișoara, Timișoara, Romania; Department of Infection Diseases I, University of Medicine and Pharmacy “Victor Babeș” Timișoara, Timișoara, Romania; National Institute of Infectious Diseases “Prof. Dr. Matei Balș”, Bucharest, Romania; Carol Davila University of Medicine and Pharmacy, Bucharest, Romania; The HIV-AIDS Center, Institute of Infectious and Tropical Disease ‘Dr Kosta Todorovic’, School of Medicine, University of Belgrade, Belgrade, Serbia; Department for Pharmacology, Clinical Pharmacology and Toxicology, School of Medicine, University of Belgrade, Belgrade, Serbia; Infectious Diseases Hospital, Cluj-Napoca, Romania; “Sf. Parascheva” Infectious Diseases Clinical Hospital, Iași, Romania; “Gr. T. Popa” University of Medicine and Pharmacy, Iași, Romania; National Institute for Infectious Diseases “Prof. Dr. Matei Balș”, Bucharest, Romania; Compartment for Monitoring and Evaluation of HIV/AIDS Data in Romania, Bucharest, Romania; National Institute for Public Health, Romania-National Centre for Surveillance and Control of Transmissible Diseases, Bucharest, Romania; Department of Infectious Diseases, Parasitology and Tropical Medicine, Nicolae Testemițanu State Medical and Pharmacy University, Chișinău, Republic of Moldova; Hospital of Dermatology and Communicable Diseases, Chișinău, Republic of Moldova; Department of Infection Diseases I, “Victor Babeș” Clinical Hospital of Infectious Diseases Timișoara, Timișoara, Romania; Department of Infection Diseases I, University of Medicine and Pharmacy “Victor Babeș” Timișoara, Timișoara, Romania; Adults 3 Department of Matei Balș National Institute of Infectious Diseases, Bucharest, Romania; Infectious Diseases Department, Carol Davila University of Medicine and Pharmacy, Bucharest, Romania; Physiopathology and Immunology Department, Carol Davila University of Medicine and Pharmacy, Bucharest, Romania; Department of Neuroscience and Recovery, Faculty of Medicine and Pharmacy, University of Oradea, Oradea, Romania; National Institute of Infectious Disease “Prof. Dr. Matei Balș”, Bucharest, Romania; Carol Davila University of Medicine and Pharmacy, Bucharest, Romania; National Institute for Infectious Diseases “Prof. Dr. Matei Balș”, Bucharest, Romania; Carol Davila University of Medicine and Pharmacy, Bucharest, Romania; Department of Microbiology, Clinical Centre of Serbia, Belgrade, Serbia; Belgrade University School of Medicine Infectious & Tropical Diseases Hospital, Belgrade, Serbia; Department of Infectious Diseases I, “Victor Babeș” University of Medicine and Pharmacy Timișoara, Timișoara, Romania; Department of Infectious Diseases, Clinical Hospital of Infectious Diseases and Pneumology “Victor Babeș”, Timișoara, Romania; Institute of Microbiology and Immunology, University of Belgrade Faculty of Medicine, Belgrade, Serbia; University Hospital for Infectious and Tropical Diseases CCS, HIV/AIDS Unit, Belgrade, Serbia; Microbiology Department, Clinical Center Serbia, Belgrade, Serbia; Department of Infectious Diseases, University of Medicine and Pharmacy Tîrgu-Mureș, Tîrgu-Mureș, Romania; “Dr. Victor Babeș” Hospital for Infectious and Tropical Diseases, Bucharest, Romania; Faculty of Sociology and Social Work, University of Bucharest, Bucharest, Romania; Faculty of Psychology and Education Sciences, University of Bucharest, Bucharest, Romania; HIV Neurobehavioral Research Center, University of California, San Diego, USA; National Institute for Infectious Diseases “Prof. Dr. Matei Balș”, Bucharest, Romania; Carol Davila University of Medicine and Pharmacy, Bucharest, Romania; Clinical Department, “Dunărea de Jos” University Galați, Galați, Romania; Infectious Diseases Clinical Hospital Galați, Galați, Romania; Morphologic and Functional Sciences Department, “Dunărea de Jos” University Galați, Galați, Romania; Clinical Department, National Institute for Infectious Diseases “Prof. Dr. Matei Balș”, Bucharest, Romania; Carol Davila University of Medicine and Pharmacy, Bucharest, Romania; Department for Molecular Diagnostic and Flow Cytometry, University Hospital for Infectious Diseases Zagreb, Zagreb, Croatia; Department of Viral Hepatitis, University Hospital for Infectious Diseases Zagreb, Zagreb, Croatia

## Abstract

O1 HIV-1 diversity in Bulgaria (current molecular epidemiological picture)

Ivailo Alexiev, Reneta Dimitrova, Anna Gancheva, Asya Kostadinova, Mariyana Stoycheva, Daniela Nikolova, Ivaylo Elenkov

O2 Knowledge, attitudes and practices of the general population on HIV/AIDS, hepatitis B and C in Romania

Cătălin Tilișcan, Mioara Predescu, Bogdan Păunescu, Anca Streinu-Cercel, Oana Săndulescu, Claudiu Mihai Șchiopu, Mădălina Hristache, Lăcrămioara Aurelia Brîndușe, Adrian Streinu-Cercel

O3 The prevalence of human leukocyte antigen-B*57:01 allele carriers and CXCR4 tropism among newly diagnosed HIV infected patients in Serbia

Marija Todorovic, Marina Siljic, Dubravka Salemovic, Valentina Nikolic, Ivana Pesic-Pavlovic, Jovan Ranin, Djordje Jevtovic, Maja Stanojevic

O4 HIV transmission among stable serodiscordant couples from the former Pediatric Cohort follow up in the National Institute of Infectious Diseases

Ana Maria Tudor, Delia Vlad, Mariana Mărdărescu, Sorin Petrea, Cristina Petre, Ruxandra Neagu-Drăghicenoiu, Rodica Ungurianu, Alina Cibea, Odette Chirilă, Cristian Anghelina, Ileana Coserea

O5 Unemployment is associated with syringe sharing among people who inject drugs in Greece

Pantelia-Amalia Krikelli, Eirini Pavlitina, Mina Psichogiou, Demetris Lamnisos, Leslie Williams, Anya Korobchuk, Britt Skaathun, Pavlo Smyrnov, John Schneider, Vana Sypsa, Dimitrios Paraskevis, Angelos Hatzakis, Samuel R. Friedman, Georgios K. Nikolopoulos

O6 Correlation of adipocytokine levels in different types of lipodystrophy in HIV/AIDS patients

Gordana Dragović, Danica Srdić, Al Musalhi Khawla, Ivan Soldatović, Jelena Nikolić, Djordje Jevtović, Devaki Nair

O7 IP10 – a possible biomarker for the progression of HIV infection

Aura Temereanca, Adelina Rosca, Luminita Ene, Benchawa Soontornniyomkij, Carmen Diaconu, Claudia Dita, Cristian Achim, Simona Ruta

O8 A permanent challenge: persistent low viremia in HIV positive patients on ART

Șerban Benea, Ruxandra Moroti, Raluca Jipa, Eliza Manea, Andrada Stan, Elisabeta Benea, Dan Oțelea, Adriana Hristea

O9 Infections in IDUs according to their HIV status

Adriana Hristea, Irina Lăpădat, Raluca Jipa, Ruxandra Moroti, Șerban Benea, Doina Antonică, Irina Panait, Roxana Petre

O10 Trends in combined antiretroviral therapy used in methadone program integrated with HIV care - 20 years of experience

Justyna D. Kowalska, Ewa Pietraszkiewicz, Ewa Grycner, Ewa Firlag-Burkacka, Andrzej Horban

O11 Extracellular cyclophilin A – inflammatory mediator in HIV infected patients

Ovidiu Vlaicu, Leontina Bănică, Simona Paraschiv, Ana-Maria Tudor, Ruxandra Moroti, Dan Oțelea

O12 High cardiovascular disease risk in Serbian population, an issue of concern

Bojana Dimitrijević, Ivan Soldatović, Đorđe Jevtović, Jovana Kusić, Dubravka Salemović, Jovan Ranin, Gordana Dragović

O13 Genotypic rifampicin resistance in HIV/ tuberculosis coinfected patients from a tertiary level infectious diseases hospital

Dragoș Florea, Ioana Bădicuț, Alexandru Rafila, Cornel Camburu, Adriana Histrea, Mihaela Frățilă, Dan Oțelea

O14 Occurrence of residual HCV RNA in liver and peripheral blood mononuclear cells among patients with chronic hepatitis C infection and/or HCV/HIV coinfection after IFN-based therapy

Ivana Gmizic, Dubravka Salemovic, Ivana Pesic-Pavlovic, Marina Siljic, Valentina Nikolic, Miljana Djonin-Nenezic, Ivana Milosevic, Branko Brmbolic, Maja Stanojevic

O15 Romanian nationwide screening for infection with HIV and hepatitis B and C viruses

Anca Streinu-Cercel, Oana Săndulescu, Alina Cristina Neguț, Mioara Predescu, Alexandra Mărdărescu, Mihai Săndulescu, Adrian Streinu-Cercel

O16 Treatment emergent variants to combined direct antiviral agents therapy against hepatitis C virus

Ana Belen Pérez, Natalia Chueca, Marta Álvarez, Juan Carlos Alados, Antonio Rivero, Francisco Vera, Marcial Delgado, Javier Salmeron, Miguel Jiménez, Maria José Blanco, Moises Diago, Miguel Garcia-deltoro, Marta Alvarez, Francisco Téllez, Federico García

O17 Clinical and epidemiological aspects in tuberculosis/HIV coinfected patients

Diana Tănase, Eliza Manea, Rodica Bacruban, Dragoș Florea, Dan Oțelea, Alexandru Rafila, Mariana Mărdărescu, Adriana Hristea

O18 Resistance to NS3 protease inhibitors in persons with chronic hepatitis C infected with hepatitis C virus subtype 1a from Croatia

Ivana Grgic, Ana Planinic, Maja Santak, Lana Gorenec, Snjezana Zidovec Lepej, Adriana Vince

O19 Analysis of a simplified diagnostic score for tuberculous meningitis in HIV-infected adults with meningitis

Eliza Manea, Adriana Hristea, Șerban Benea, Ruxandra Moroti, Diana Tănase, Cristian M. Niculae, Simona Merisor, Raluca Jipa

O20 Molecular tracing of the origin of HIV-1 infection among persons who inject drugs in Athens: a phyloethnic study

Dimitrios Paraskevis, Evangelia Kostaki, Georgios K. Nikolopoulos, Vana Sypsa, Mina Psichogiou, Dimitra Paraskeva, Athanassios Skoutelis, Meni Malliori, Samuel R. Friedman, Angelos Hatzakis

O21 The dynamics of virological response to HIV-1 infection and antiretroviral therapy initiation in patients with and without HLA-B*5701 Allele

Malgorzata Hackiewicz, Piotr Zabek, Ewa Firlag-Burkacka, Andrzej Horban, Justyna Dominika Kowalska

O22 Increase in the numbers of non-B subtypes and potential recombinant forms circulating among Slovenian MSM in the recent years

Maja M. Lunar, Jana Mlakar, Mario Poljak

O23 Genotyping intrahost polymorphisms in hepatitis C virus E2 protein associated with resistance to antibody neutralization

Leontina Bănică, Eliza Martin, Valeriu Gheorghiță, Andrei Petrescu, Dan Oțelea, Costin-Ioan Popescu, Simona Paraschiv

O24 Genotyping of HCV NS3 protease inhibitors resistance and phenotyping of rare double resistance mutations in HCV cell culture system

Emil Neaga, Vlaicu Ovidiu, Andrei Juncu, Leontina Bănică, Simona Paraschiv, Dan Oțelea, Costin-Ioan Popescu

O25 Employment status controls the relationship between neurocognitive impairment and depression in a cohort of young HIV-infected adults since childhood

Adrian Luca, Florin Lazăr, Anca Elena Luca, Luminița Ene, Cristian Achim

O26 Predictors of survival in parenterally-infected HIV positive children and youth diagnosed with progressive multifocal leukoencephalopathy

Cosmina Gingăraş, Ștefan Adrian Anton, Roxana Rădoi, Simona Tetradov, Grațiela Țârdei, Maria Nica, Razvan Alexandru Capşa, Cristian L. Achim, Cristiana Oprea, Luminița Ene

O27 Neurocognitive and brain functioning in HIV-infected young MSM treated with cART

Bogna Szymańska, Natalia Gawron, Agnieszka Pluta, Emilia Łojek, Ewa Firląg – Burkacka, Andrzej Horban, Robert Bornstein, et HARMONIA3 Study Group

O28 Clinical value of RT-PCR detection of *Toxoplasma gondii* DNA in cerebrospinal fluid

Olivia Burcoș, Simona Manuela Erscoiu, Filofteia Bănicioiu Cojanu, Andreea Toderan, Maria Nica, Ionuț Cristian Popa, Emanoil Ceaușu, Petre Iacob Calistru

O29 Characteristics of sleep disorders in Romanian adults infected with human immunodeficiency virus

Manuela Arbune, Mirela Alexandrache, Anca-Adriana Arbune, Doina-Carina Voinescu

O30 Diagnosing neuroHIV: the rift between clinicians and pathologists

Ioan-Alexandru Diaconu, Laurențiu Stratan, Victoria Aramă, Luciana Nichita, Alexandra Diaconu, Anca Negru, Alina Orfanu, Anca Leuștean, Daniela Adriana Ion

O31 A challenging neurological complication in a HIV-infected young woman with multiple opportunistic infections

Irina Ianache, Cristiana Oprea

O32 Brain abscess with uncertain etiology in a late-presenter HIV infected patient

Anca Leuștean, Cristina Popescu, Alina Orfanu, Anca Negru, Remulus Catana, Cristina Murariu, Ioan-Alexandru Diaconu, Mihaela Rădulescu, Cătălin Tilișcan, Victoria Aramă

O33 Cerebral toxoplasmosis and left crural monoparesis with fatal evolution in a noncompliant patient with AIDS C3

Iosif Marincu, Patricia Poptelecan, Valeria Bică, Florin Lazăr, Livius Tirnea

O34 Opportunistic infections still a problem in HIV-infected patients in cART era: a Romanian single center experience

Irina Ianache, Roxana Rădoi, Manuela Nica, Grațiela Țârdei, Luminița Ene, Emanoil Ceaușu, Petre Calistru, Cristiana Oprea

P1: Epidemiological aspects of co-infection of HIV/TB in Moldova

Iurie Osoianu, Ala Halacu

P2 Perinatal exposure at HIV infection in Oltenia region

Andreea Cristina Stoian, Florentina Dumitrescu, Iulian Diaconescu, Augustin Cupșa, Lucian Giubelan, Loredana Ionescu, Irina Niculescu

P3 Women living with HIV in Mureș county

Carmen Chiriac, Nina Șincu, Iringo Zaharia Kezdi, Anca Georgescu, Brândușa Țilea, Cristina Girbovan, Andrea Incze, Andrea Fodor

P4 Late diagnosis of HIV infection in children - a challenge for Romania

Alina Cibea, Mariana Mărdărescu, Cristina Petre, Ruxandra Drăghicenoiu, Rodica Ungurianu, Ana Maria Tudor, Delia Vlad, Carina Matei

P5 Cirrhosis Assessment in Patients Co-infected HIV-Hepatitis B Virus

Elena Dumea, Lucian Cristian Petcu, Simona Claudia Cambrea

P6 HIV late presenters in Craiova Regional Center, Romania

Florentina Dumitrescu, Augustin Cupsa, Andreea Cristina Stoian, Lucian Giubelan, Irina Niculescu, Iulian Diaconescu, Dan Hurezeanu, Livia Dragonu, Mioara Cotulbea

P7 Some aspects of malignancies in patients HIV / AIDS

Simona Manuela Erscoiu, Ionuț Cristian Popa, Denisa Stroie, Petronela Ionescu, Nedeea Duță, Camelia Dobrea, Irina Voican, Emanoil Ceaușu, Petre Iacob Calistru

P8 Factors associated with resilience among people living with HIV in Romania

Florin Lazăr

P9 Fever in HIV-infected patients: a thorny problem to be solved by the clinicians

Lucian Giubelan, Augustin Cupșa, Iulian Diaconescu, Florentina Dumitrescu, Dan Hurezeanu, Livia Dragonu, Irina Niculescu, Andreea Cristina Stoian, Oana Obretin, Mariana Stănescu, Mihai Jianu

P10 Th1, Th2, Th9, Th17 and Th22 cytokines in acute and chronic HIV-1 infection

Lana Gorenec, Snjezana Zidovec Lepej, Ivana Grgic, Ana Planinic, Janja Iscic Bes, Adriana Vince, Josip Begovac

P11 Dyslipidemia in HIV-infected patients treated with protease inhibitors – case report

Luminița Elena Horga

P12 Why use less treatment for the metabolic abnormalities in HIV patients-too many drugs?

Corina Itu, Luminița Elena Horga, Laura Augusta David-Aldea, Anca Ciorogar, Cristian Jianu, Mihaela Lupșe

P13 Sacral Herpes Zoster, with hyperalgesic form, in a patient with C3 stage HIV infection

Iuliana Caramangiu, Ovidiu Roșca, Monica Cialma, Andreea Ardeleanu, Iosif Marincu

P14 Factors associated with in-hospital mortality in tuberculous and cryptococcal meningitis

Raluca Jipa, Eliza Manea, Șerban Benea, Irina Lăpădat, Nicoleta Irimescu, Irina Panait, Cristian Niculae, Adriana Hristea

P15 Lipodystrophy: still present adverse event in resource-limited settings

Jovana Kusic, Djordje Jevtovic, Dubravka Salemovic, Jovan Ranin, Bozana Dimitrijevic, Gordana Dragovic

P16 TB and HIV coinfected patient, an emergent challenge - case report

Laura-Augusta Aldea-David

P17 Efficacy of prophylactic antiretroviral treatment in new-born infants from HIV-positive mothers in 2012-2014, for the North-Eastern part of Romania

Carmen Manciuc, Cristina Nicolau, Liviu Prisăcariu, Alexandra Largu

P18 Surveillance of mother to child transmission of HIV in Romania – 31 December 2015

Mariana Mărdărescu, Adrian Streinu-Cercel, Cristina Petre, Marieta Iancu, Sanda Vintilă, Daniela Vitelaru, Iosif Ionel, Claudiu Mihai Șchiopu, Alexandra-Henriette Mărdărescu

P19 The antiretroviral therapy failure and the need to select the effective treatment in the Republic of Moldova

Pavel Micsanschi, Tiberiu Holban, Ina Bîstrițchi, Lucia Pârțână, Angela Nagîț, Svetlana Popovici, Maria Talmaci, Irina Cucerova

P20 Disseminated cryptococcosis in a patient with C3 HIV stage and multiresistant to antiretroviral therapy with lethal evolution

Sorina Georgiana Mitrescu, Dana Mihalcea, Iulia Caramangiu, Ovidiu Roșca, Iosif Maricu

P21 Aspects of tuberculosis infection in HIV-positive patients from Romania – our experience

Anca Negru, Daniela Munteanu, Victoria Aramă, Raluca Mihăilescu, Ioan Diaconu, Remulus Catana, Cristina Popescu, Alina Orfanu, Anca Leuștean, Mihaela Rădulescu, Cătălin Tilișcan, Raluca Năstase, Violeta Molagic, Irina Duport, Cristina Dragomirescu, Ștefan Sorin Aramă

P22 Dyslipidemia in HIV-infected patients

Nicoleta M Negruț

P23 Challenges in the management of an HIV seropositive patient with psoriasis undergoing immunomodulator therapy

Violeta Elena Niță, Daniela Ioana Munteanu, Raluca Mihăilescu, Ioan Diaconu, Anca Negru, Cristina Popescu, Victoria Aramă

P24 Acute peritonitis as a sign of IRIS in an HIV-infected patient with MAC latent infection

Alina Orfanu, Cristina Popescu, Anca Leuștean, Anca Negru, Remulus Catana, Ioan Diaconu, Cătălin Tilișcan, Victoria Aramă, Sorin Ștefan Aramă

P25 The virologic outcome of the treatment of chronic hepatitis B among HIV co-infected patients on HAART

Ivana Pesic Pavlovia, Dubravka Salemovic, Jovan Ranin, Djordje Jevtovic

P26 A case of HIV encephalopathy with aphasia, agnosia, apraxia and right homonymous hemianopsia

Ovidiu Roșca, Andreea Ardeleanu, Iulia Caramangiu, Daniela Desaga, Valerica Bică, Sorina Mitrescu, Iosif Marincu

P27 Molecular footprints on human immunodeficiency virus -1 genome and association with phylogenetic clustering among subtype B infected patients in Serbia

Marina Siljic, Dubravka Salemovic, Valentina Nikolic, Djordje Jevtovic, Ivana Pesic-Pavlovic, Jovan Ranin, Marija Todorovic , Maja Stanojevic

P28 Neurosyphilis and human immunodeficiency virus infection: double challenge

Nina-Ioana Șincu, Anca Georgescu, Brândușa Țilea, Iringo Zaharia Kezdi, Andrea Incze, Cristina Gârbovan, Carmen Lucia Chiriac

P29 Differences between HIV-infected adults since childhood and non HIV-infected persons on managing everyday life

Anca Elena Luca, Florin Lazăr, Adrian Luca, Luminița Ene, Roxana Rădoi, Adina Talnariu, Silvia Suciu, Cristian Achim

P30 Molecular detection of *Bartonella quintana* in a HIV immunodepressed patient with fever and isolated lymphadenopathy - Case report

Diana Gabriela Iacob, Dragoș Florea, Simona Iacob

P31 Present epidemiological characteristics of HIV/AIDS newly diagnosed cases in South-Eastern Romania

Manuela Arbune, Miruna Drăgănescu, Alina Iancu

P32 The gender’s preferences among opportunists?

Ruxandra Moroti, Cristian M Niculae, Simona Merisor, Eliza Manea, Serban Benea, Andrada Stan, Raluca Hrisca, Raluca Jipa, Diana Tanase, Adriana Hristea

P33 Polymorphism of interleukin-28B gene in persons with chronic hepatitis C from Croatia

Ivana Grgic, Ana Planinic, Lana Gorenec, Snjezana Zidovec Lepej, Adriana Vince

## ORAL PRESENTATIONS

### Session title: epidemiology, subtypes, regional issues

#### O1 HIV-1 diversity in Bulgaria (current molecular epidemiological picture)

##### Ivailo Alexiev^1^, Reneta Dimitrova^1^, Anna Gancheva^1^, Asya Kostadinova^1^, Mariyana Stoycheva^2^, Daniela Nikolova^3^, Ivaylo Elenkov^4^

###### ^1^National HIV Reference Laboratory, National Center of Infectious and Parasitic Diseases, Sofia, Bulgaria; ^2^Department of Infectious Diseases, Medical University, Plovdiv, Bulgaria; ^3^Clinic of Infectious Diseases, Medical University, Varna, Bulgaria; ^4^Hospital for Infectious and Parasitic Diseases, Sofia, Bulgaria

####### **Correspondence:** Ivailo Alexiev (ivoalexiev@yahoo.com) – National HIV Reference Laboratory, National Center of Infectious and Parasitic Diseases, Sofia, Bulgaria

**Objectives**

In Bulgaria 1606 cases with HIV/AIDS were diagnosed from 1986 until 2012. Epidemiological data indicated greater heterogeneity of HIV-1 positive population, including minority groups, migrants, and recent increase of men who have sex with men (MSM) and intravenous drug users (IDUs). The aim of the present study was to investigate the current molecular epidemiological picture of HIV-1 diversity among different transmission groups in Bulgaria.

**Materials and Methods**

HIV-1 diversity was analyzed in 637 (39.7 %) of all HIV/AIDS patients in the country. HIV-1 pol sequences were generated with TruGene and/or ViroSeq Genotyping Systems. The sequence alignment contained Bulgarian sequences and reference sequences from Los Alamos and GenBank. Phylogenetic relationships were inferred by ML and Bayesian analysis. Recombinations were analyzed with bootscan analysis using SimPlot software.

**Results**

The most prevalent HIV-1 strain in the studied population was found to be subtype B 38.8 %. That strain was followed by CRF01_AE 20.3 %, CRF02_AG 13.8 %, subtype C 3.6 %, subtype A1 1.6 % and the rest of strains were defined as 3 different HIV-1 subtypes (F, G and H) and 7 circulating recombinant forms (05_DF, 14_BG, 03_AB, 04_cpx, 12_BF, 33_01B and 36_cpx). In addition, 30 different unique recombinant forms consisting of 18.2 % were identified. We found that unlike in the most European countries, where subtype B is dominating, non-B HIV-1 subtypes were the most prevalent in Bulgaria. Phylogenetic analysis showed multiple clusters demonstrating rapid development of sub-epidemics in different transmission groups. In addition, unequal distribution of HIV-1 clades across different regions and transmission groups of the populations were found. Moreover, we found that various viral clades were introduced in Bulgaria from abroad by migrants.

**Conclusions**

Our analysis revealed wide HIV-1 diversity and unequal distribution of different strains among MSM, IDUs and heterosexual individuals. Phylogenetic analysis showed multiple clusters demonstrating development of local-epidemics. Significant number of HIV-1 clades were introduced from abroad by migrants. Our findings indicated that providing of detailed molecular epidemiological surveillance of HIV-1 in Bulgaria is of great importance to better understand the epidemic in the country.

#### O2 Knowledge, attitudes and practices of the general population on HIV/AIDS, hepatitis B and C in Romania

##### Cătălin Tilișcan^1,2^, Mioara Predescu^1^, Bogdan Păunescu^3^, Anca Streinu-Cercel^1,2^, Oana Săndulescu^1,2^, Claudiu Mihai Șchiopu^1^, Mădălina Hristache^1^, Lăcrămioara Aurelia Brîndușe^4^, Adrian Streinu-Cercel^1,2^

###### ^1^National Institute for Infectious Diseases “Prof. Dr. Matei Balș”, Bucharest, Romania; ^2^Carol Davila University of Medicine and Pharmacy, Bucharest, Romania; ^3^Totem Communications, Bucharest, Romania; ^4^National Institute of Public Health, Bucharest, Romania

####### **Correspondence:** Cătălin Tilișcan (catalin.tiliscan@gmail.com) – National Institute for Infectious Diseases “Prof. Dr. Matei Balș”, Bucharest, Romania

**Background**

Hepatitis B (HBV), hepatitis C (HCV) and HIV infections are important global public health issues. Assessing the level of knowledge, attitudes and practices regarding HIV, HBV and HCV infections in the general population represents a very important basis for an effective strategy that aims to inform and raise awareness in this field.

**Methods**

The study was based on two query methods: an opinion poll and a face to face interview based on a questionnaire applied by the interview operator to two target populations – adults over 18 years of age from general population and injecting drug users from Bucharest. There were two sample groups - a probabilistic, bistadial, stratified group consisting of 1005 respondents from the general population (GP) and a group of 200 injecting drug users (IDUs). The questionnaires were processed by TOTEM Communications during August-September 2015.

**Results**

Most respondents know about HIV/AIDS and hepatitis B and C. One of five respondents from the general population considers that hepatitis B/C is caused by lack of hygiene compared to only 1 % of IDUs. In terms of transmission risks, IDUs have higher knowledge on ways of transmission of HIV/AIDS, HBV, HCV as they benefitted from detailed information in this area. Three quarters of IDUs shared injecting equipment with other persons. Persons from the general population living in rural areas shared needles with other persons more than their peers in urban locations. Approximately a third of respondents from the general population are comfortable around a person infected with HIV/HBV/HCV compared with over eight out of ten IDUs. Half of the respondents from the GP would conceal that one of their kin is infected with HIV. Two of ten respondents from GP and three out of ten IDUs consider that a child infected with HIV should not be granted access to school. Almost three quarters of respondents disapprove of a teacher’s academic career if he or she is infected with HIV, HBV or HCV. Three quarters of respondents in the target populations would get a vaccine against HBV.

**Conclusions**

We noted significant differences between GP and IDUs regarding knowledge and attitudes on HIV, HBV and HCV infections.

Acknowledgement

This study is part of the RO 19.02. Project “Strengthening the prevention and control of HIV/AIDS, HBV, HCV in Romania”, financed by the Norway Financial Mechanism 2009-2014, “Public Health Initiatives”.

#### O3 The prevalence of human leukocyte antigen-B*57:01 allele carriers and CXCR4 tropism among newly diagnosed HIV infected patients in Serbia

##### Marija Todorovic^1^, Marina Siljic^1^, Dubravka Salemovic^2^, Valentina Nikolic^1^, Ivana Pesic-Pavlovic^3^, Jovan Ranin^1,2^, Djordje Jevtovic^1,2^, Maja Stanojevic^1^

###### ^1^University of Belgrade Faculty of Medicine, Belgrade, Serbia; ^2^Infectious and Tropical Diseases University Hospital, Clinical Center Serbia, HIV/AIDS Unit, Belgrade, Serbia; ^3^Virology Department, Clinical Center Serbia, Belgrade, Serbia

####### **Correspondence:** Marija Todorovic (mtodorovic5@gmail.com) – University of Belgrade Faculty of Medicine, Belgrade, Serbia

**Background**

Abacavir is an effective antiretroviral drug, among the most commonly used nucleoside reverse transcriptase inhibitor (NRTI) in Serbia. Although generally well tolerated, a percentage of patients experience a potentially life-threatening hypersensitivity reaction (HSR), shown to be associated with the presence of the class I MHC allele, HLA B*57:01. Genotyping for HLA B*57:01 prior to starting abacavir is nowadays recommended in international HIV treatment guidelines for avoiding abacavir-induced HSR. On the other hand, CCR5 (R5) tropic viruses are known to be associated with early stages of infection, whereas CXCR4 (X4) HIV-1 tropic strains prevail in advanced infection. Recently, a change in the genotype-predicted tropism frequency in early disease has been described with increased frequency of non R5 strains. The aim of our study was to estimate the prevalence of HLA B*57:01 allele and genotype-predicted viral tropism among newly diagnosed HIV-1 infected patients in Serbia.

**Methods**

The presence of HLA B*57:01 allele was analyzed in 273 HIV-1 infected patients aged 18 or more, who were abacavir naive. Buccal swab samples were obtained from all participants and assayed for the presence of HLA B*57:01 using a commercial HLA B*57:01 real time PCR kit. Baseline plasma samples were used to assess the HIV-1 genotypic tropism with triplicate V3-loop sequencing. The non-R5 tropism prediction thresholds were assigned using a false positive rate (FPR) of 10 %.

**Results**

The presence of HLA B*57:01 allele was found in 22 of 273 tested individuals (8 %, 95 % CI 5.4-11.9 %). The overall non-R5 tropism frequency was 15.4 %.

**Conclusions**

Abacavir still remains one of the key drugs of antiretroviral regimens against HIV-1 in Serbia as in other countries. This is the first study that estimated HLA-B*57:01 prevalence in Serbia. Very high prevalence of HLA B*57-01 found in our study, strongly supports HLA B*57:01 genotyping that should be implemented prior to initiation of abacavir containing therapy to reduce the risk of potentially life threatening hypersensitivity reaction. R5 tropism predominates among the treatment of naive individuals, however, the frequency of non-R5 tropic variants needs to be monitored.

Acknowledgement

This work was partially funded by the Ministry of Education and Science Republic of Serbia (grant MNTR175024).

#### O4 HIV transmission among stable serodiscordant couples from the former Pediatric Cohort follow up in the National Institute of Infectious Diseases

##### Ana Maria Tudor^1,2^, Delia Vlad^1^, Mariana Mărdărescu^1^, Sorin Petrea^1^, Cristina Petre^1^, Ruxandra Neagu-Drăghicenoiu^1^, Rodica Ungurianu^1^, Alina Cibea^1^, Odette Chirilă^1^, Cristian Anghelina^1^, Ileana Coserea^1^

###### ^1^National Institute for Infectious Diseases “Prof. Dr. Matei Balș”, Bucharest, Romania; ^2^Carol Davila University of Medicine and Pharmacy, Bucharest, Romania

####### **Correspondence:** Ana Maria Tudor (tudoranamaria@yahoo.com) – National Institute for Infectious Diseases “Prof. Dr. Matei Balș” Bucharest, Romania

**Background**

In the last years, patients from the former Romanian Pediatric Cohort engaged in stable couples. Some of those couples are serodiscordant. Our aim is to evaluate the risk of HIV transmission among those patients.

**Methods**

We retrospectively collected the data on results of HIV tests performed in patients’ partners followed up in the Pediatric HIV Department between 2012 and 2015. We also collected the data on viral load in HIV positive patients and the declared use of condoms in studied partners.

**Results**

We identified 81 serodiscordant couples, in 70 couples one partner was part of the former Pediatric Cohort. The incidence of HIV infection in patients’ partners was 2.4 % (2/81cases). In these two couples, the male partner was diagnosed with HIV in early childhood, but at the moment of partner diagnosis the viral load was high (5log_10_), since they refused the antiretroviral treatment. In our study 35/81 (43.2 %) of discordant couples the male was positive. During the studied period we encountered 33 pregnancies. Just one couple used safe methods of conception, sperm washing. The use of condoms was declared in 24 discordant couples. 8 out of 35 (22.8 %) HIV positive males and 17 out of 49 (34.7) positive females declared the use of condoms.

**Conclusions**

One third of our patients used condoms in HIV discordant couples, but we found only 2 cases of transmission. We need to focus more on education for HIV prevention and safe conception in our patients in the context of patients’ desire of starting a traditional family.

#### O5 Unemployment is associated with syringe sharing among people who inject drugs in Greece

##### Pantelia-Amalia Krikelli^1^, Eirini Pavlitina^2^, Mina Psichogiou^3^, Demetris Lamnisos^1^, Leslie Williams^4^, Anya Korobchuk^5^, Britt Skaathun^6^, Pavlo Smyrnov^5^, John Schneider^6^, Vana Sypsa^7^, Dimitrios Paraskevis^7^, Angelos Hatzakis^7^, Samuel R. Friedman^4^, Georgios K. Nikolopoulos^2,8^

###### ^1^European University of Cyprus, Nicosia, Cyprus; ^2^Transmission Reduction Intervention Project, Athens, Greece; ^3^Laikon General Hospital, Department of Propedeutic Medicine, Athens, Greece; ^4^National Development and Research Institutes, New York, NY, USA; ^5^Alliance for Public Health, Kyiv, Ukraine; ^6^University of Chicago Medical Center, Center for AIDS Elimination, Chicago, IL, USA; ^7^Medical School, University of Athens, Athens, Greece; ^8^Hellenic Centre for Disease Control and Prevention, Amarousio, Greece

####### **Correspondence:** Dimitrios Paraskevis (dparask@med.uoa.gr) – Medical School, University of Athens, Athens, Greece

**Background**

Greece has been hit hard by a serious economic and social crisis. An HIV outbreak among people who inject drugs (PWID) started early in 2011 and resulted in more than 1000 new diagnoses by 2013. This work studied the association between needle/syringe sharing and unemployment and homelessness, which comprise two salient characteristics of economic instability.

**Methods**

Data on PWID were collected by a social network tracing project (Transmission Reduction Intervention Project - TRIP). Baseline interviews were conducted between June 2013 and July 2015 in Athens, Greece. The statistical analyses included chi-squared test and logistic regression modeling. The multivariable models included age, sex, level of education, nationality, employment, homelessness, married status, sexual orientation, years of injection and knowledge about recent HIV infection.

**Results**

322 PWID (mean age 35.7 ± 8 years, females 18.9 %) were interviewed. The proportion of PWID who reported they never used syringes someone else had used before was 64 %. The proportion of never sharing was lower among participants who were non-Greeks (41.9 %) vs. Greeks (66.3 %, p = 0.05), homeless (52.6 %) vs. with stable accommodation (67.5 %, p = 0.07), without a job (69.1 %) or unable to work because of drug use or illness (57.4 %) vs. those who were employed (81.3 %; p = 0.05), and with elementary education (55.6 %) vs. high school (67 %) or university graduates (85.7 %, p = 0.26). Age, education level and employment retained their significance in multivariable logistic regression. The adjusted odds ratios (OR) for never sharing were: for the unemployed vs. the employed: 0.36; 95 % Confidence Interval (CI): 0.17-0.79; for age (per year increase) 1.05; 95 % CI: 1.02-1.08; and for high school/university education vs. elementary 1.68; 95 % CI: 1.02-2.74.

**Conclusion**

Needle/syringe programs scaled-up after the start of the outbreak in 2011 but unemployed PWID in 2013-2015 were 64 % less likely to report they never shared syringes than PWID who were economically active. The unemployment rate in Greece is higher than 25 % and shows no signs of decreasing. Efforts to help PWID get a job may reduce risky behaviors among PWID in an unstable setting, and following a serious HIV outbreak.

**Funding**

US National Institute on Drug Abuse (NIDA) (Grant: DP1 DA034989 and Hellenic Scientific Society for the Study of AIDS and STDs).

## ORAL PRESENTATIONS

### Session title: comorbidities, ageing with HIV

#### O6 Correlation of adipocytokine levels in different types of lipodystrophy in HIV/AIDS patients

##### Gordana Dragović^1^, Danica Srdić^1^, Al Musalhi Khawla^2^, Ivan Soldatović^3^, Jelena Nikolić^4^, Djordje Jevtović^4^, Devaki Nair^2^

###### ^1^Department of Pharmacology, Clinical Pharmacology and Toxicology, School of Medicine, University of Belgrade, Belgrade, Serbia; ^2^Department of Clinical Biochemistry, Royal Free Hospital and University College London, London, United Kingdom; ^3^Institute for Biomedical Statistics, School of Medicine, University of Belgrade, Belgrade, Serbia; ^4^Infectious and Tropical Diseases Hospital, School of Medicine, University of Belgrade, Belgrade, Serbia

####### **Correspondence:** Gordana Dragović (gozza@beotel.net) – Department of Pharmacology, Clinical Pharmacology and Toxicology, School of Medicine, University of Belgrade, Belgrade, Serbia

**Background**

Combination antiretroviral therapy (cART) can induce metabolic complications including lipodystrophy, dyslipidemia, and insulin resistance in HIV-infected patients. Adipokines may play important roles in these alterations, especially in lipodystrophy. The aim of this study was to evaluate the relationship between serum levels of adipocytokines in four different categories of fat distribution: lipoatrophy (LA), lipohypertrophy (LH), mixed fat redistribution (MFR) and no-lipodystrophy in HIV-infected patients.

**Methods**

Cross-sectional study of 66 HIV-infected adults. Levels of adiponectin, resistin, leptin, interleukins (IL-1α, IL-1β, IL-2, IL-4, IL-6, IL-8, IL-10), plasminogen-activator-inhibitor-1 (PAI-1), C-peptide, cystatin-C, tumor necrosis factor alpha (TNF-α), vascular-endothelial-growth-factor (VEGF), epidermal-growth-factor (EGF), interferon-gamma (IFN-γ) and monocyte-chemoattractant-protein-1 (MCP-1) were measured. Differences between groups were tested using t-test and Mann-Whitney test, and analysis of covariance to examine relationship between adiponectin and leptin and lypodystrophy categories adjusted for confounding variables.

**Results**

The lipodystrophy was observed in 29 (44 %) patients, while 15 (52 %) of them had LA, 4 (14 %) had LH and 10 (34 %) patients had MFR. LH patients had higher levels of adiponectin (p = 0.011), leptin (p = 0.039), cystatin-C (p = 0.001), IL-6 (p = 0.065), and lower levels of IL-4 (p = 0.052). LA patients had lower levels of IL-4 (p = 0.043), IL-10 (p = 0.031) and IL-1α (p = 0.051). Correlation of adiponectin with lipodystrophy remains statistically significant in the subgroup of patients with lipohypertrophy after adjustment for age, BMI, cystatin-C, PAI-1, IFN-γ (p = 0.001).

**Conclusions**

Adiponectin was shown to be an important marker in fat disturbances in patients undergoing cART and directly associated with lipohypertrophy, and could be the possible target for new therapeutic strategies in HIV/AIDS patients.

#### O7 IP10 – a possible biomarker for the progression of HIV infection

##### Aura Temereanca^1,2^, Adelina Rosca^1,2^, Luminita Ene^3^, Benchawa Soontornniyomkij^4^, Carmen Diaconu^2^, Claudia Dita^2^, Cristian Achim^4^, Simona Ruta^1,2^

###### ^1^Carol Davila University of Medicine and Pharmacy, Bucharest, Romania; ^2^“Stefan S. Nicolau” Institute of Virology, Bucharest, Romania; ^3^“Dr Victor Babeș” Hospital for Infectious and Tropical Diseases, Bucharest, Romania; ^4^University of California San Diego, La Jolla, San Diego, CA, USA

####### **Correspondence:** Aura Temereanca (auratemereanca@yaoo.com) – Carol Davila University of Medicine and Pharmacy, Bucharest, Romania

**Background**

IP10 (interferon gamma-induced protein) is associated with chronic immune activation and HIV-1 viral replication. Our study evaluates the relationship between IP10 and immunologic and virologic status in a group of extensively-treated HIV-infected Romanian patients, long term survivors.

**Materials and methods**

90 HIV+ subjects (median age: 23 years, males: 53.3 %) with a median duration on cART of 12 years were included. Paired plasma and CSF samples were available for 45 patients. Current median HIV viral load and CD4 count were 1.53 log_10_ HIV RNA copies/mL and 464.16 cells/cmm (range 2-1720), respectively. 43 % had undetectable viral load and 38 % had CD4 > 500 cells/cmm. Plasma and CSF concentration of IP-10 was measured using multiplex assay on the Meso Scale Discovery platform (Meso Scale Diagnostics, LLC. Gaithersburg, MD). Patients were classified into two groups based on the median threshold value of IP10 (median = 444 pg/mL; IQR = 296-756 pg/mL): group 1 with higher and group 2 with lower values, respectively.

**Results**

Plasma and CSF IP10 levels were positively correlated with HIV viral load (rho = 0.54, p < 0.001; rho = 0.58, p < 0.002) and the degree of immune suppression (rho = 0.45, p < 0.001; rho = 0.32, p < 0.03). The group of patients with higher plasma and CSF IP10 levels had significantly higher median HIV RNA (0 log copies/mL vs. 2.46 log copies/mL; p < 0.001) and significantly lower median time from VL Zenith (5 vs. 8 years; p = 0.01). Elevated IP10 levels were associated with lower CD4 T-cell counts (419 vs. 663 cells/cmm, p < 0.001) and lower CD4:CD8 ratio (57 % vs. 36 %, p = 0.03). No differences were observed among the 2 groups in terms of sex, median age, CD4 count nadir, AIDS-defining events (stage C) and cumulative ART exposure. Patients with active viral replication (HIV viral load > 1000 copies/mL) had significantly higher concentrations of IP10 in both plasma and CSF compared with those presenting undetectable HIV RNA (1007 vs. 376 pg/mL, p < 0.0001; 4943 vs. 557 pg/mL, p = 0.04).

**Conclusions**

Elevated IP10 levels are associated with high viral load and immune suppression in heavily-treated HIV-infected Romanian patients, suggesting that IP10 could be a marker of HIV-1 disease progression.

#### O8 A permanent challenge: persistent low viremia in HIV positive patients on ART

##### Șerban Benea^1,2^, Ruxandra Moroti^1,2^, Raluca Jipa^1^, Eliza Manea^1^, Andrada Stan^1^, Elisabeta Benea^1,2^, Dan Oțelea^1^, Adriana Hristea^1,2^

###### ^1^National Institute for Infectious Diseases “Prof. Dr. Matei Balș”, Bucharest, Romania; ^2^Carol Davila University of Medicine and Pharmacy, Bucharest, Romania

####### **Correspondence:** Șerban Benea (serbanb_16@yahoo.com) – National Institute for Infectious Diseases “Prof. Dr. Matei Balș”, Bucharest, Romania

**Background**

With new and potent antiretroviral drugs, the goal of maintaining a HIV viral load below the limits of assay detection seems to be within reach. Nevertheless, persistent low-level viremia (LLV) remains a challenge for clinicians, with unclear consequences regarding virologic failure and resistance mutations.

The main goal of our study was to identify and describe characteristics associated with persistent low-level viremia in HIV infected patients from one department.

**Methods**

We performed a retrospective, descriptive study, analyzing the records of HIV infected patients in our department, Adult Clinic IV of the National Institute for Infectious Diseases “Prof. Dr. Matei Balș” over a period of 15 years (2001-2015). The inclusion criteria for our patients were: antiretroviral therapy (ART) for more than 24 weeks, with two consecutive detectable viral loads (VL) of >20 and <1000 copies/mL. We analyzed demographical data, CDC stage, the immune-virological status (last evaluation and at baseline), ART regimen (drugs used and duration).

**Results**

Out of 153 patients monitored in our department, 143 (93.4 %) are on ART. Eighty-seven (60.8 %) patients on ART had undetectable VL at the last evaluation, including 23 of them (26.4 %) that had at least one episode of persistent LLV followed by undetectable VL and 18 (20.6 %) had one or more VL blips. Fifty-six patients had detectable VL at the last evaluation in 2015. Eighteen (12.6 %) of patients on ART met the inclusion criteria for our study. Most patients (66.6 %, n = 12) were men and the median age was 39 years. Based on CDC classification 4 patients (22.2 %) were A, 8 (44.4 %) were B and 6 were C (33.3 %). The median nadir CD4 cell count was 172 cells/cmm (IQR, 86-240) and the median baseline VL was 5.66 log_10_ (IQR, 5.09 log_10_-6.38 log_10_). The median duration of ART was 36 months (IQR, 12-96). Our patients received a median number of 2 (IQR, 1-4) ART regimens. Only 7 (39 %) patients had at least one undetectable VL while on ART. At the moment of our study, all patients were on a regimen containing two NRTIs combined with: a PI (boosted- lopinavir, n = 3; boosted-atazanavir, n = 3, boosted-darunavir, n = 10), an integrase inhibitor (n = 1) or a NNRTI (n = 1). All patients experienced an increase in the CD4 cell count, with the current median value of 574 cells/cmm (IQR, 403-739). The last median VL was 52 copies/mL (IQR, 25-179). Notably, all our patients with a persistent LLV had less than 500 copies/mL, although the median number of detectable VL was 3 (IQR, 2-8).

**Conclusions**

Despite new regimens containing potent drugs, LLV remains a problem for HIV positive patients on ART. Clinicians need to closely monitor this category of patients while further studies will be required to investigate the consequences of persistent LLV.

#### O9 Infections in IDUs according to their HIV status

##### Adriana Hristea^1,2^, Irina Lăpădat^1^, Raluca Jipa^1^, Ruxandra Moroti^1,2^, Șerban Benea^1,2^, Doina Antonică^1^, Irina Panait^1^, Roxana Petre^3^

###### ^1^National Institute for Infectious Diseases “Prof. Dr. Matei Balș”, Bucharest, Romania; ^2^Carol Davila University of Medicine and Pharmacy, Bucharest, Romania; ^3^National Institute of Aerospace Medicine, Bucharest, Romania

####### **Correspondence:** Adriana Hristea (adriana_hristea@yahoo.com) – National Institute for Infectious Diseases “Prof. Dr. Matei Balș”, Bucharest, Romania

**Background**

The introduction of new psychoactive substances on the Romanian market in 2008 was associated with high risk behavior for HIV acquisition compared to opioid abuse, which resulted in an increased number of new HIV infections among injecting drug users (IDUs). One hundred and forty nine (21 %) patients were IDUs out of a total of 698 newly diagnosed with HIV infection in Romania, in 2015. Our aim was to analyze the characteristics of IDUs according to their status for HIV infection.

**Methods**

We performed a retrospective descriptive study, analyzing the records of IDUs hospitalized between January and December 2015 in our institution.

**Results**

We identified 93 IDUs, of which 79 (85 %) tested positive for HIV infection. Seventeen (22 %) HIV infected patients were diagnosed in 2015. There was an overall male predominance in both groups, 64 (81 %) in HIV infected patients and 12 (86 %) in HIV non-infected patients. Fifteen (19 %) HIV infected patients were on opioid substitution therapy versus 4 (29 %) HIV non-infected patients (p = 0.3, OR: 0.6, 95%CI: 0.2-2.1). The median age was similar in both groups, 31 (IQR: 26-35) in HIV infected patients versus 30 years (IQR: 25-33) in HIV non-infected patients (p = 0.7). The median CD4 cell count was 92 (IQR: 12-340). Most of the patients (81 %) were in stage B and C of HIV disease. Antiretroviral treatment (ART) coverage was of 37 %. Median length of hospitalization was 13 days (IQR: 5-30) in HIV infected patients vs 7 days (IQR: 3-10) in HIV non-infected patients (p = 0.1). Chronic hepatitis B was identified in 6 (8 %) HIV infected vs 2 (14 %) HIV non-infected patients (p = 0.3, OR: 0.5, 95%CI: 0.09-2.7) and chronic hepatitis C in 66 (84 %) HIV infected vs 12 (86 %) in HIV non-infected patients (p = 0.5, OR: 0.8, 95%CI: 0.2-4.2). The main causes of hospitalization were: cellulitis in 27 (34 %) HIV infected vs 2 (14 %) HIV non-infected patients (p = 0.3, OR: 3.4, 95%CI: 0.2-46.4); pneumonia in 30 (38 %) HIV infected vs 4 (29 %) HIV non-infected patients (p = 0.3, OR: 1.5, 95%CI: 0.4-5.3); tuberculosis in 21 (27 %) HIV infected vs 1(7 %) HIV non-infected patients (p = 0.5, OR: 1.7, 95%CI: 0.1-20.7) and endocarditis in 9 (11 %) HIV infected patients. Nine (11 %) HIV infected patients died during hospitalization.

**Conclusion**

The majority of IDUs were young men, with HIV/HCV co-infection. Most HIV infected IDUs were hospitalized in a late stage of HIV infection and had a longer duration of hospitalization. The ART coverage in this population is very low. Cellulitis, pneumonia and tuberculosis were more frequently seen in HIV-infected patients.

#### O10 Trends in combined antiretroviral therapy used in methadone program integrated with HIV care - 20 years of experience

##### Justyna D. Kowalska^1,2^, Ewa Pietraszkiewicz^1,2^, Ewa Grycner^1^, Ewa Firlag-Burkacka^1^, Andrzej Horban^1,2^

###### ^1^Hospital for Infectious Diseases, HIV Out-Patient Clinic, Warsaw, Poland; ^2^Medical University of Warsaw, Department for Adult's Infectious Diseases, Warsaw, Poland

####### **Correspondence:** Justyna D. Kowalska (jdkowalska@gmail.com) – Hospital for Infectious Diseases, HIV Out-Patient Clinic, Warsaw, Poland

**Background**

In 1995 a methadone program was started at HIV clinic as integrated services. The main purpose was to increase uptake, retention and adherence to cART among HIV-infected IDU in Central Poland. Here we evaluate trends in antiretroviral therapy regimens used across 20 years of observation.

**Methods**

This is a retrospective analysis of observational database cohort of cART naive, HIV-infected adults enrolled at registration to HIV care. The cohort has been established with the purpose of prospective, active observation of HIV-positive patients routinely followed in HIV Out-Patient Clinic, in the Hospital of Infectious Diseases in Warsaw. Data are collected in real-time from the clinic database and include demographic characteristics, history of clinical visits, cART history and results of all tests performed in the clinic. In statistical analyses Chi-squared and Kruskal-Wallis tests were used as appropriate for group comparisons, all tests of significance were two-sided. Confidence interval (CI) of 95 % was accepted. All analyses were performed using SAS version 9.4 (SAS Institute, Cary, NC, USA).

**Results**

In total 267 (6.5 %) of 4062 patients registered in HIV Out-Patient Clinic entered integrated methadone program (MET). There was a significant difference between MET and non-MET groups in terms of age at registration (median 35.2 [IQR: 30.3 - 41.2] vs. 33.27 [28.5 -40.0] years; p = 0.004), baseline lymphocyte CD4 count (440 [255 - 619] vs. 370 [211 - 531] cells/uL; p < 0.0001), nadir CD4 count (129 [49 - 217] vs. 242 [114 - 359]; p < 0.0001), baseline HIV RNA (4.16 [3.22 - 4.80] vs. 4.31 [3.39 - 4.93] log copies/mL; p = 0.012), most recent CD4 count (362 [207 - 560] vs. 503 [346 - 672]; p < 0.0001) and most recent HIV RNA (1.69 [1.27 - 2.96] vs. 1.69 [1.27 - 1.97]; p = 0.006). In general MET participants had longer person-years of follow-up (9.4 [6.0 - 12.7] vs. 4.8 [1.9 - 8.7]; p < 0.0001) and were longer on cART (7.6 [3.8 - 12.0] vs. 3.2 [1.0 - 7.0] years; p < 0.0001). Of all MET patients 251 (94.0 %) started cART, 199 (79.3 %) with protease inhibitor (PI) and 52 (20.7 %) with non-nucleoside reverse transcriptase inhibitor (NNRTI), p = 0.53. Among non-MET patients 3179 (83.3 %) started cART, 2227 (70.0 %) PI based, 853 (26.8 %) NNRTI based, 68 (2.1 %) integrase inhibitor (INI) based and 31 (1.0 %) fusion inhibitors based regimen, p < 0.0001 (for cART group comparison between MET and non-MET p = 0.003).

**Conclusions**

MET participants were more likely to start PI-containing cART, as compared to non-MET patients. The utilization of INI is not yet an option used for MET patients. Data from clinical trials are showing no drug-drug interactions between methadone and INI, thus encouraging to move this experience into clinical practice. Forming observational cohorts investigating the effects of this new drug group, especially in respect to adverse drug reactions, seems to be rational and valid approach.

#### O11 Extracellular cyclophilin A – inflammatory mediator in HIV infected patients

##### Ovidiu Vlaicu^1^, Leontina Bănică^1^, Simona Paraschiv^1^, Ana-Maria Tudor^2^, Ruxandra Moroti^3^, Dan Oțelea^1^

###### ^1^Molecular Diagnostic Laboratory, National Institute for Infectious Diseases “Prof. Dr. Matei Balș”, Bucharest, Romania; ^2^Pediatric Department, National Institute for Infectious Diseases “Prof. Dr. Matei Balș”, Bucharest, Romania; ^3^Clinical Department III, National Institute for Infectious Diseases “Prof. Dr. Matei Balș”, Bucharest, Romania

####### **Correspondence:** Ovidiu Vlaicu (vlaicu.ovidiu@yahoo.com) – Molecular Diagnostic Laboratory, National Institute for Infectious Diseases “Prof. Dr. Matei Balș”, Bucharest, Romania

**Background**

Cyclophilin A (CypA) is known for its intracellular roles in protein folding, trafficking and T cell activation. However, a soluble form of CypA was reported to be secreted as a result to inflammation. CypA proinflammatory potential was validated in some pathologic and physiologic conditions (cardiovascular diseases, rheumatoid arthritis, sepsis, cancer, ageing). Based on these facts we aimed to evaluate the level of CypA and the soluble form of its receptor CD147 (sCD147) in HIV infected patients and their association with inflammation.

**Methods**

Plasma samples from 29 HIV infected patients and 17 healthy controls (HC) were tested for levels of IL-6, C-reactive protein (CRP), CypA, sCD147 by ELISA. Statistical evaluation of the data was performed with Man Whitney t test and Spearman correlation test, in GraphPad Prism and p values < 0.05 were considered significant.

**Results**

HIV infected patients showed higher levels of inflammatory markers (CRP and IL-6) as compared to HC, while CypA and sCD147 levels were not significantly different. However, when HIV infected patients were stratified according to viral loads (VL), those with detectable VL had significantly higher levels of plasmatic CypA. As expected, HIV infected patients with detectable VL had significantly lower CD4 T cell counts and CD4/CD8 ratios than those with undetectable VL. CypA levels correlated negatively with CD4/CD8 ratios and positively with VL; sCD147 was directly correlated with CD8 counts in HIV infected patients.

**Conclusion**

HIV infection may induce CypA secretion with a positive feedback effect on inflammation.

Acknowledgment

This study was partly supported by UEFISCDI, Grant HIV-ID (260/2015) and POSCCE Program, CRCBABI Project (642/2014).

#### O12 High cardiovascular disease risk in Serbian population, an issue of concern

##### Bozana Dimitrijević^1^, Ivan Soldatović^2^, Đorđe Jevtović^3^, Jovana Kusić^1^, Dubravka Salemović^3^, Jovan Ranin^3^, Gordana Dragović^1^

###### ^1^Department of Pharmacology, Clinical Pharmacology and Toxicology, School of Medicine, University of Belgrade, Belgrade, Serbia; ^2^Institute for Biomedical Statistics, School of Medicine, University of Belgrade, Belgrade, Serbia; ^3^Infectious and Tropical Diseases Hospital, School of Medicine, University of Belgrade, Belgrade, Serbia

####### **Correspondence:** Bozana Dimitrijević(drdimitrijevicb@gmail.com) – Department of Pharmacology, Clinical Pharmacology and Toxicology, School of Medicine, University of Belgrade, Belgrade, Serbia

**Background**

Cardiovascular disease (CVD) and coronary heart disease (CHD) have become major causes of morbidity and mortality in HIV infected patients, due to the usage of combination antiretroviral therapy (cART) as well as the pro-inflammatory effects of HIV infection, and traditional risk factors. In addition of this, Serbia is a country with one of the highest rates of age-standardized mortality from CVD and CHD within South Eastern Europe in general population, with almost no primary prevention programmes on National level. Thus the aim of this study was to estimate CVD and CHD risk scores in HIV infected patients receiving cART in the HIV-treatment center in Belgrade (HCB).

**Methods**

A cross-sectional analysis of 202 consecutive HIV infected patients aged between 40 and 79 years who received antiretroviral therapy for at least 6 months at the HCB. We estimated CVD and CHD risk scores using Data Collection on Adverse Effects of Anti-HIV Drugs study (DAD) model prevalence of cardiovascular risks in HIV-patients. The study was approved by the Clinical Center of Serbia Ethics Committee.

**Results**

There were 51 (25.2 %) females and 151 (74.8 %) males included in the study. Clinical AIDS was observed in 89 (43.9 %) patients. The median CD4+ T-cells count was 461 (IQR = 194-625) cells/mm^3^. The prevalence of current smoking, hypertension and hypercholesterolemia (>6.2 mmol/L) were 100 (49.5 %), 64 (31.5 %) and 72 (35.4 %), respectively. Fifty-one (25.2 %) persons were overweight, 15 (7.4 %) were obese, 45 (22.3 %) had metabolic syndrome and diabetes 7 (3.5 %). Forty-three (21.3 %) persons were eligible for statin therapy according to EACS (95 % confidence intervals [CI], 16.3 % to 27.4 %). A high 5-year DAD CVD and CHD risk scores were 2.5 % (95 % CI 1.2 % - 6 %) and 2.1 % (95 % CI 1 % - 5.2 %), respectively. High 10-year DAD CVD and CHD risk scores were 6.8 % (95 % CI 2.8 % - 15.5 %) and 5.4 % (95 % CI 2.5 % - 13.5 %), respectively. While high (>20 %) DAD CVD and DAD CHD risk scores were recorded in 35 (17.3 %) and 24 (11.8 %) patients, respectively.

**Conclusion**

We have demonstrated that almost one third of patient population included in this study has elevated CVD and CHD risk scores in accordance with the DAD model. Thus, intervention for reducing high risk and preventive activities needs to be done in next coming period.

## ORAL PRESENTATIONS

### Session title: coinfections

#### O13 Genotypic rifampicin resistance in HIV/ tuberculosis coinfected patients from a tertiary level infectious diseases hospital

##### Dragoș Florea^1,2^, Ioana Bădicuț^1^, Alexandru Rafila^1,2^, Cornel Camburu^1^, Adriana Histrea^1,2^, Mihaela Frățilă^1^ and Dan Oțelea^1^

###### ^1^National Institute for Infectious Diseases “Prof. Dr. Matei Balș”, Bucharest, Romania; ^2^Carol Davila University of Medicine and Pharmacy, Bucharest, Romania

####### **Correspondence:** Dragoș Florea (dflorea2002@yahoo.com) – National Institute for Infectious Diseases “Prof. Dr. Matei Balș”, Bucharest, Romania

**Background**

Tuberculosis occurs frequently and has a major prognostic impact in HIV patients. Rapid and reliable diagnostic methods for *Mycobacterium tuberculosis* infection are important both for adequate treatment decisions and timely infection control procedures. The objective of the present study was to analyze the rate of tuberculosis coinfection and rifampicin resistance in HIV infected patients from a tertiary level infectious diseases hospital in Romania.

**Methods**

Retrospective analysis of tuberculosis coinfection in HIV positive patients admitted in the National Institute for Infectious Diseases, Bucharest, Romania from January 2014 to December 2015. The diagnosis of tuberculosis and rifampicin resistance was based on genotypic (MTB/RIF Xpert) and routine phenotypic methods (microscopy, Lowenstein-Jensen cultivation).

**Results**

A number of 190 samples from HIV positive patients were received over a two years period. Pulmonary samples were predominant, but non-pulmonary samples were also collected: sputum (36.8 %), cerebrospinal fluid (30 %), bronchial aspirate (20.5 %), pleural effusion (4.2 %), lymph node aspirate (3.1 %), broncho-alveolar lavage (2.6 %). In 56 samples from 54 patients *M. tuberculosis* was detected using genotypic and/or phenotypic methods. The positivity rate was 35 % for pulmonary samples and 21 % for non-pulmonary samples. Genotypic rifampicin resistance was detected in 10 HIV infected patients (18.5 %), most of them with pulmonary tuberculosis (7 cases).

**Conclusions**

Rifampicin resistance is detected in a high proportion of HIV/tuberculosis coinfected patients from a tertiary level infectious diseases hospital. Genotypic methods able to provide a rapid diagnosis of tuberculosis infection and rifampicin resistance are needed in the management of HIV patients.

Acknowledgments

This study was partly supported by POSCCE Program, CRCBABI Project (642/2014).

#### O14 Occurrence of residual HCV RNA in liver and peripheral blood mononuclear cells among patients with chronic hepatitis C infection and/or HCV/HIV coinfection after IFN-based therapy

##### Ivana Gmizic^1,2^, Dubravka Salemovic^1^, Ivana Pesic-Pavlovic^1^, Marina Siljic^2^, Valentina Nikolic^2^, Miljana Djonin-Nenezic^1^, Ivana Milosevic^1^, Branko Brmbolic^1^, Maja Stanojevic^2^

###### ^1^Clinic for Infectious and Tropical Diseases, Clinical Centre of Serbia, Belgrade, Serbia; ^2^Faculty of Medicine, University of Belgrade, Belgrade, Serbia

####### **Correspondence:** Ivana Gmizic (gmizic_ivana@yahoo.com) – Clinic for Infectious and Tropical Diseases, Clinical Centre of Serbia, Belgrade, Serbia

**Background**

An entity of occult HCV infection has been described in various studies in past decade defining it as detectable HCV-RNA in the liver or peripheral blood mononuclear cells (PBMCs) in the absence of detectable serum or plasma HCV-RNA. Still, the clinical significance of these findings remains uncertain, in both treated and untreated patients. Treatment with pegylated interferon (IFN) plus ribavirin (RBV) has long been standard of care for patients with chronic hepatitis C virus (HCV) infection. Usually, liver biopsy was used a prerequisite for starting therapy. Response to this therapy is known to depend on the HCV genotype, with rates of sustained virological response (SVR) in 40-50 % of genotype 1-infected and in about 70-80 % of genotype 2-infected individuals. This rate is lower in case of persisting HCV/HIV co-infection.

**Material/methods**

The aim of this study was to investigate the occurrence of occult HCV infection among patients with chronic HCV infection and/or HCV/HIV coinfection after achieved SVR with IFN based therapy in Serbia. We report preliminary results of this study. We examined the presence of HCV RNA in liver tissue and PBMC in 47 patients who underwent liver re-biopsy after successful treatment with combination peginterferon plus ribavirin, that yielded negative plasma viremia. All patients were over 18 years old and consenting to participate in the study.

**Results**

Average age of included patients was 45 ± 13.6 (range 26-73), whereas 29/47 patients were male (61.7 %). Pre-therapy genotyping revealed genotype 1 as the most common one, with 57.4 % (n = 27/47), followed by genotype 3 with 34 % (n = 16/47), genotype 2 and 4 in 4.3 % (n = 2/47). The most common risk was intravenous drug use and surgical procedures (25.5 %). Average therapy duration was 37.4 weeks, whereas re-biopsy was performed on average 24.3 months post therapy completion (range 2-80 months). There were 5 patients (10.6 %) with HCV/HIV coinfection, 4/5 were male with average age 46 ± 14.8, with genotype 3 as predominant, and 7.6 ± 8.2 months passed after therapy till re-biopsy performed. Our preliminary findings demonstrated HCV RNA in 3/47 (6.38 %) liver samples where 2/3 had pre-therapy genotype 3, including one patient who had HCV/HIV coinfection.

**Conclusions**

Residual HCV RNA is present in 6.38 % in this study including one of the patients, who has HIV as coinfection. Further studies are underway.

Acknowledgement

Funded as project 157024, by Ministry of Education, Science and Technological development.

#### O15 Romanian nationwide screening for infection with HIV and hepatitis B and C viruses

##### Anca Streinu-Cercel^1,2^, Oana Săndulescu^1,2^, Alina Cristina Neguț^1,2^, Mioara Predescu^2^, Alexandra Mărdărescu^3^, Mihai Săndulescu^1^, Adrian Streinu-Cercel^1,2^

###### ^1^Carol Davila University of Medicine and Pharmacy, Bucharest, Romania; ^2^National Institute for Infectious Diseases “Prof. Dr. Matei Balș”, Bucharest, Romania; ^3^Romanian HIV Center, Bucharest, Romania

####### **Correspondence:** Anca Streinu-Cercel (anca_sc@yahoo.com) – Carol Davila University of Medicine and Pharmacy, Bucharest, Romania

**Introduction**

Despite the introduction of national immunization against HBV since 1996 through neonatal vaccination and catch-up regimens, hepatitis B remains prevalent and underdiagnosed in Romania in 2016. Hepatitis C and HIV infection also remain important public health concerns and require the implementation of nationwide screening studies to ascertain real-life incidences and the extent to which they are underdiagnosed.

**Methods**

We have implemented a study to screen for hepatitis B and C viruses (HBV, HCV) and HIV infection in the general population in Romania. Patients were referred to the National Institute for Infectious Diseases “Prof. Dr. Matei Balș” from the general practitioner (GP). The GPs were previously trained by infectious diseases specialists and received support during the program. All subjects voluntarily signed a study-specific informed consent form and filled out a questionnaire assessing risk factors for transmission of HBV, HCV and HIV. We present the results of the first 9937 patients screened in this study from May to August 2015.

**Results**

The tested population was made up of 36 % males and 64 % females, with a mean age of 48 years old ± 29 years. Out of the total number of 9937 persons tested, 540 were found positive for HBsAg (5.4 %), 267 for anti-HCV (2.7 %), and 11 (0.1 %) had positive ELISA tests for HIV infection. However, viral loads for HBV DNA, HCV RNA and HIV RNA were negative in 230 (42.6 %), 104 (39.0 %) and 2 (18.2 %) cases, respectively. The Romani population group had good addressability to general practitioners and to the screening study. Notably, the incidence of HBV, HCV and HIV infection was lower in the Romani group than in the rest of the patients: 2/186 (1.1 %) HBV, 1/186 (0.5 %) HCV, 0/186 (0 %) HIV infection vs. 308/9751 (3.2 %) HBV, 162/9751 (1.7 %) HCV and 9/9751 (0.1 %) HIV infection.

**Conclusion**

The preliminary results of the study characterized the relative incidence of HBV, HCV and HIV infection in Romania. The study group was representative, and the age distribution was normal, suggesting that the data are valid for the general population in Romania, although specific high-risk groups still need to be defined. The relative incidences of HIV and hepatitis B and C in this study were fairly low, and the Romani population had lower incidences compared with the rest of the group, suggesting that these viral infections may still be underdiagnosed in the general population.

#### O16 Treatment emergent variants to combined direct antiviral agents therapy against hepatitis C virus

##### Ana Belen Pérez^1^, Natalia Chueca^1^, Marta Álvarez^1^, Juan Carlos Alados^2^, Antonio Rivero^3^, Francisco Vera^4^, Marcial Delgado^5^, Javier Salmeron^6^, Miguel Jiménez^7^, Maria José Blanco^8^, Moises Diago^9^, Miguel Garcia-deltoro^10^, Francisco Téllez^11^, Federico García^1^

###### ^1^Servicio de Microbiologia. CHUG, Hospital San Cecilio, Instituto de investigacion IBS, Granada, Spain; ^2^Servicio de Microbiologia. Hospital de Jerez de la Frontera, Cádiz, Spain; ^3^Servicio de Enfermedades Infecciosas, Hospital Reina Sofia, Cordoba, Spain; ^4^Servicio de Enfermedades Infecciosas, Hospital Santa Lucia, Cartagena, Spain; ^5^Servicio de Enfermedades Infecciosas, Hospital Regional Carlos Haya, Malaga, Spain; ^6^UGC Digestivo, HUSC, Granada, Spain; ^7^UGC Digestivo, Hospital Regional Carlos Haya, Malaga, Spain; ^8^UGC Enfermedades Digestivas, Hospital de Jerez de la Frontera, Cádiz, Spain; ^9^Servicio de Ap. Digestivo, H General de Valencia, Valencia, Spain; ^10^Unidad de Enfermedades Infecciosas, H General de Valencia, Valencia, Spain; ^11^Servicio de Medicina Interna, Hospital de la Linea, Cadiz, Spain

####### **Correspondence:** Federico García (fegarcia@ugr.es) – Servicio de Microbiologia. CHUG, Hospital San Cecilio, Instituto de investigacion IBS, Granada, Spain

**Background**

Treatment emergent variants (TEVs) to first line direct antiviral agents against HCV may represent a problem of major concern. The information on real life setting is scarce and may vary from that of clinical trials. AASLD now recommend testing for Q80K and NS5a RAVs in patients that, having failed first line regimens, have cirrhosis and/or are in need of urgent retreatment. Here we describe the first data of TEVs in a real life setting in Spain.

**Patients & Methods**

We conducted an observational study, including all patients referred to our Reference Center for antiviral resistance from across Spain, from July to November 2015. TEVs were investigated using population sequencing on the failing sample, and in parallel stored baseline samples, when available. Genotype specific primers were used for NS5a (codons 1-99; positions 28, 29, 30, 31, 32, 58, 62, 92 & 93) and NS3 sequencing (codons 1-181; positions 36, 43, 55, 56, 80, 122, 155, 156, 168 & 170). Pangenotypic primers were used for NS5b sequencing, which was also used for re-genotyping.

**Results**

31 patients failing an interferon free DAA combination were included. Most of them were male (86.2 %) with a median age of 53 (IQR 50-61), and a median HCV viral load of 6.22 logs (IQR 5.64-6.49). 8 patients were HCV GT1a, 14 GT1b, 5 GT 3a and 4 GT4d. 15 patients had failed to Sofosbuvir-Simeprevir based regimens, and 16 to a Sofosbuvir-NS5a inhibitor combination (Daclatasvir or Ledipasvir). We did not observe any Sofosbuvir TEV. TEVs were observed in 9 of the 15 patients failing Simeprevir based regimens (60.0 %), including S122T, n = 1; D168H, n = 1; D168V, n = 4; V36D + D168V, n = 1; Q80K + R155K, n = 1; Q80K + A156S, n = 1. For patients on an NS5A inhibitor based regimen, TEVs were found for 14 out of 16 patients (87.5 %), including Q30H, n = 2; Q30K, n = 1; Q30R, n = 1; L31M, n = 1; Y93H, n = 4; L31M + Y93H, n = 2; L31V + Y93H, n = 2; T28A + Q30R, n = 1. NS5b, NS3 & NS5A sequencing, reclassified two patients initially genotyped as GT1b as GT1a. Reinfection was confirmed using massive parallel sequencing and phylogenetic analysis on baseline (GT1a) and relapse samples (GT3a).

**Conclusions**

We present the first results on the genetic barrier and TEV characterization of patients that relapse to Sofosbuvir based regimens in Spain. The high genetic barrier to Sofosbuvir was confirmed, as well as a high prevalence of NS5a associated TEVs. We have also shown the ability of resistance assays to detect errors in genotyping by commercial genotyping assays, as well as to document reinfection rather than relapse.

#### O17 Clinical and epidemiological aspects in tuberculosis/HIV coinfected patients

##### Diana Tănase, Eliza Manea, Rodica Bacruban, Dragoș Florea, Dan Oțelea, Alexandru Rafila, Mariana Mărdărescu, Adriana Hristea

###### National Institute for Infectious Diseases “Prof. Dr. Matei Balș”, Bucharest, Romania

####### **Correspondence:** Diana Tănase (dianapetrache07@yahoo.com) – National Institute for Infectious Diseases “Prof. Dr. Matei Balș”, Bucharest, Romania

**Background**

In Romania HIV and tuberculosis (TB) epidemics are independent and most of the TB patients are not infected with HIV. Nevertheless, the increasing prevalence of HIV infection, especially in high-risk populations like intravenous drug users (IDUs), may also contribute to an increased number of TB co-infected patients. In Romania, 113 of the 698 new HIV patients, were co-infected with TB in 2015.

Objective

The aim of this study was to describe the demographic, clinical profile and the HIV status of the patients diagnosed with TB in a tertiary infectious disease hospital.

**Methods**

We studied retrospectively adults with suspected or confirmed TB, hospitalized in our institution between January and December 2015. We collected demographic, clinical and laboratory data. TB confirmation was based on routine microbiological methods and/or GeneXPERT.

**Results**

Out of 114 studied patients 63 (55.3 %) were HIV-positive, 40 (35.1 %) HIV negative and 11 (9.6 %) with status unknown. Male patients were predominant in both groups: 68.3 % in HIV infected and 57.5 % in HIV non-infected group, respectively (p = 0.1). The median age was 32 (IQR 27-39) in HIV-positive patients compared with 39 (IQR 28-62) in HIV-negative patients (p < 0.01). A quarter (23.8 %) of the 63 TB/HIV co-infected patients were IDUs. Thirteen (20.6 %) TB/HIV co-infected patients were newly diagnosed with HIV and were very late presenters, with a median CD4 of 33 cells/cmm (IQR 16-108). There was no significant difference between the HIV-positive and HIV negative group regarding TB localization. Pulmonary TB (PTB) was found in 54 % versus 50 %, extra-pulmonary TB (EPTB) in 27 % versus 38 % and PTB associated with EPTB were found 19 % versus 12 % of cases. TB was confirmed in 47 (74.6 %) HIV infected patients versus 23 (57.5 %) non-infected patients. Microbiologically confirmed TB (smear/culture) was seen in 26 (55.3 %) versus 10 (43.4 %) in HIV-infected versus HIV non-infected respectively (p = 0.06). TB was confirmed by PCR in 61 samples, 40 (63.5 %) in HIV infected and 21 (52.5 %) in HIV non-infected patients (p = 0.1). Ten (16.4 %) samples were resistant to rifampicin, 8 (80 %) of them isolated from HIV-infected patients. A short duration from admission to a positive diagnosis of TB (5.2 days) was obtained for both groups.

**Conclusion**

More than half of newly diagnosed TB patients were HIV-coinfected. HIV/TB patients were younger, but there was no difference between the two groups regarding the TB localization. We noticed a similar proportion of EPTB regardless of HIV status. We found a high rate of rifampicin resistance mainly in HIV-infected patients.

Acknowledgments

This study was partly supported by POSCCE Program, CRCBABI Project (642/2014).

#### O18 Resistance to NS3 protease inhibitors in persons with chronic hepatitis C infected with hepatitis C virus subtype 1a from Croatia

##### Ivana Grgic^1^, Ana Planinic^1^, Maja Santak^3^, Lana Gorenec^1^, Snjezana Zidovec Lepej^1^, Adriana Vince^2^

###### ^1^Department for Molecular Diagnostic And Flow Cytometry, University Hospital for Infectious Diseases Zagreb, Zagreb, Croatia; ^2^Department of Viral Hepatitis, University Hospital for Infectious Diseases Zagreb, Zagreb, Croatia; ^3^Centre for Research and Knowledge Transfer in Biotechnology, Laboratory for Molecular Biomedicine, University of Zagreb, Zagreb, Croatia

####### **Correspondence:** Ivana Grgic (ivanahobby@gmail.com) – Department for Molecular Diagnostic And Flow Cytometry, University Hospital for Infectious Diseases Zagreb, Zagreb, Croatia

**Background**

An NS3 inhibitor simeprevir is one of the treatment options for chronic hepatitis C in Croatia (in combination with pegylated interferon alpha and ribavirin). Mutation Q80K in persons infected with HCV subtype 1a is associated with reduced treatment response to simeprevir. The aim of this study was to analyze the frequency of Q80K mutations in persons with HCV subtype 1a infection prior to treatment with direct acting antivirals and to analyze other mutations associated with HCV resistance to NS3 inhibitors.

**Methods**

The study included 85 persons with chronic hepatitis C infected with HCV subtype 1a receiving clinical care at the Department of Viral Hepatitis of the University Hospital for Infectious Diseases, Zagreb and Croatian Reference Center for Viral Hepatitis. HCV subtype was determined by using Inno LiPA genotyping test. Detection of Q80K and other mutations associated with resistance to NS3 inhibitors was performed as a part of pre-treatment diagnostic workup by population-based sequencing on ABI PRISM - 3100 Genetic Analyzer. Geno2Pheno algorithm was used for the interpretation of resistance analysis results.

**Results**

Resistance to Simeprevir was detected in 36 of 85 (42 %) of patients. Subtype 1a clade I was detected in 41 patients and clade II in 44 patients. Mutation Q80K was detected only in patients infected with HCV subtype 1a clade I (32 of 41 patients). The majority of patients with clade I (n = 21) were infected with strains carrying Q80K mutation only whereas 11 patients carried a combination of Q80K and other mutations associated with NS3 resistance mutations. Mutations associated with resistance to NS3 inhibitors excluding Q80K were detected in 4 of 44 patients infected with subtype 1a clade II. The patterns of resistance mutations included: 155 T and 170 V (n = 1 patient), 36 L, 155 K and 170 V (n = 1), 138A, 155S, 168G, 170C and 43S (n = 1) and 36 L (n = 1, possible resistance).

**Conclusion**

The results of this study have shown a high prevalence of Q80K in patients with chronic hepatitis C infected with clade I subtype 1a from Croatia. The contribution of other mutations to NS3 inhibitors resistance was limited.

#### O19 Analysis of a simplified diagnostic score for tuberculous meningitis in HIV-infected adults with meningitis

##### Eliza Manea^1^, Adriana Hristea^1,2^, Șerban Benea^1,2^, Ruxandra Moroti^1,2^, Diana Tănase^1^, Cristian M. Niculae^1^, Simona Merisor^1^, Raluca Jipa^1^

###### ^1^National Institute for Infectious Diseases “Prof. Dr. Matei Balș”, Bucharest, Romania; ^2^Carol Davila University of Medicine and Pharmacy, Bucharest, Romania

####### **Correspondence:** Eliza Manea (elizamanea10@yahoo.com) – National Institute for Infectious Diseases “Prof. Dr. Matei Balș”, Bucharest, Romania

**Background**

Tuberculous meningitis (TBM) and cryptococcal meningitis (CM) are the main causes of meningitis in HIV infected adults. A simplified diagnostic score (SDS) based on four variables was created as a prediction rule to differentiate patients with TBM from those with viral meningitis. (Hristea et al Int J Tuberc Lung Dis, 2012) The four variables were: duration of symptoms before admission of ≥5 days, presence of neurological impairment (altered consciousness, seizures, mild focal signs, multiple cranial nerve palsies, dense hemiplegia or paraparesis), CSF/blood glucose ratio <0.5 and CSF protein level > 100 mg/dL).We examined if the SDS would correctly identify TBM vs CM in HIV-infected patients.

**Methods**

Retrospective study of HIV-infected patients hospitalized between January 2012-December 2015 for TBM or CM. Patients were classified according to the consensus definition and scoring system published by Marais into three categories: definite (positive CSF culture for *M. tuberculosis* and/or positive commercial nucleic acid amplification test), probable and possible. CM diagnosis was established upon positive India ink stain, culture and/or cryptococcal antigen assay.

**Results**

We identified 17 patients with TBM and 18 patients with CM. Out of the 17 TBM patients 13 (77 %) had definite, 3 (17 %) probable and 1 (6 %) possible TBM. All patients with TBM had a duration of symptoms before admission of ≥5 days; presence of neurological impairment in 11(65 %); CSF/blood glucose ratio <0.5 in 15 (88 %); CSF protein level >100 mg/dL in 13 (76 %). The SDS correctly identified 16 (94 %) cases (including all cases of microbiologically documented TBM). In patients with CM, 12 (67 %) patients had a duration of symptoms before admission of ≥5 days, 14 (78 %) patients had neurological impairment, 13 (72 %) patients had CSF /blood glucose ratio <0.5 and 6 (33 %) patients had CSF protein level >100 mg/dL. The SDS would have labeled 13 (72 %) patients with CM as TBM.

The SDS sensitivity was 94.1 (95CI 71.3-99.8) and the specificity 27,8 (95CI 9.7-53.5) in differentiating TBM vs CM.

**Conclusions**

In HIV-infected patients the SDS does not satisfactorily differentiate between the two causes of chronic meningitis, TBM and CM.

Acknowledgments

This study was partly supported by POSCCE Program, CRCBABI Project (642/2014).

## ORAL PRESENTATIONS

### Session: resistance, tropism, laboratory monitoring

#### O20 Molecular tracing of the origin of HIV-1 infection among persons who inject drugs in Athens: a phyloethnic study

##### Dimitrios Paraskevis^1^, Evangelia Kostaki^1^, Georgios K. Nikolopoulos^2^, Vana Sypsa^1^, Mina Psichogiou^3^, Dimitra Paraskeva^3^, Athanassios Skoutelis^4^, Meni Malliori^5^, Samuel R. Friedman^6^, Angelos Hatzakis^1^

###### ^1^Department of Hygiene, Epidemiology and Medical Statistics, Medical School, University of Athens, Athens, Greece; ^2^Hellenic Center for Disease Control and Prevention, Amarousio, Greece; ^3^Laikon General Hospital, Department of Propedeutic Medicine, Athens, Greece; ^4^Fifth Department of Internal Medicine and Infectious Diseases, Evangelismos Hospital Athens, Athens, Greece; ^5^Medical School, University of Athens, Athens, Greece; ^6^National Development and Research Institutes (NDRI), New York, USA

####### **Correspondence:** Dimitrios Paraskevis (dparask@med.uoa.gr) – Department of Hygiene, Epidemiology and Medical Statistics, Medical School, University of Athens, Athens, Greece

**Background**

High numbers of HIV-1 infections among people who inject drugs (PWIDs) have been diagnosed in Athens, Greece since 2011. We aimed to trace the geographic origin of HIV-1 infection for migrant PWIDs and to investigate if transmissions occur more frequently among individuals with a non-Greek origin versus those between migrants and Greeks.

**Methods**

Our sample included 2,977 HIV-1 infected individuals diagnosed in Southern Greece between 1/1/2011-31/10/2014. Phylogenetic analyses used maximum likelihood method. The hypothesis of ethnic compartmentalization was tested by reconstructing ancestral states of characters at the tips using the criterion of parsimony over a set of bootstrap trees (statistical phyloethnic study).

**Results**

In our sample, 29.4 % (N = 874) were PWIDs. Phylogenetic analyses showed the existence of four major PWID-local transmission networks (LTNs): CRF14_BG (456, 58.3 %), CRF35_AD (149, 19.1 %), subtype B (118, 15.1 %), and subtype A (59, 7.5 %). The target population was 190 individuals (184 non-Greek PWIDs and 6 non-PWID migrants) who had been infected within the PWID-LTNs. 150 (78.9 %) of our target population belonged to LTNs and therefore the origin of their infection was assumed to be in Greece. Similarly, 24 persons (12.6 %) with recombinants with at least one subtype identified in LTNs were probably infected in Greece. The putative origin of infection for 16 unclustered sequences was in: Greece (subtypes A, B, CRF01_AE; N = 7; 3.7 %), Romania (subtype F, CRF14_BG; N = 2; 1.1 %), Turkey and Bulgaria (URF A/U; N = 1; 0.5 %), Greece or Israel (CRF03_AB; N = 1; 0.5 %), Turkey (unclassified sequences, N = 4; 2.1 %), and a non-Greek region (unclassified; N = 1; 0.5 %). Statistical phyloethnic analysis provided evidence for significant ethnic compartmentalization for subtype A and CRF14_BG.

**Conclusion**

Our analysis showed that the majority (95.3 %) of infections among migrants (PWIDs and non-PWIDs) clustered within PWID-LTNs originated in Greece. Our findings suggest that across different ethnic groups, HIV infections are more likely to have been locally acquired. This is a novel finding and contrasts with patterns of HIV spread among migrants (non-B subepidemic) in Europe. We also showed the existence of significant transmission networking between migrants. These findings could have public health implications, especially in PWID-related epidemics.

**Funding**

US National Institute on Drug Abuse (NIDA) (Grant: DP1 DA034989 and Hellenic Scientific Society for the Study of AIDS and STDs).

Acknowledgements

The study was implemented under NSRF 2007-2013 and cofounded by European Social Fund and national resources. Additional financial support was provided by the Hellenic Scientific Society for the Study of AIDS and STDs and the grant “Preventing HIV Transmission by Recently-Infected Drug Users” project (NIH National Institute of Drug Abuse grant DP1 DA034989).

#### O21 The dynamics of virological response to HIV-1 infection and antiretroviral therapy initiation in patients with and without HLA-B*5701 Allele

##### Malgorzata Hackiewicz^1^, Piotr Zabek^2^, Ewa Firlag-Burkacka^2^, Andrzej Horban^1,2^, Justyna Dominika Kowalska^1,2^

###### ^1^Department for Adults Infectious Diseases, Medical University of Warsaw, Warsaw, Poland; ^2^HIV Out-Patient Clinic, Hospital for Infectious Diseases, Warsaw, Poland

####### **Correspondence:** Malgorzata Hackiewicz (hackiewiczmalgorzata@gmail.com) – Department for Adults Infectious Diseases, Medical University of Warsaw, Warsaw, Poland

**Background**

HLA-B*5701 is linked to better control of HIV-1 replication. We investigate the dynamics in HIV replication and suppression in relation to HLA-B*5701 and antiretroviral therapy (ART).

**Methods**

HIV Out-Patient Clinic database collects information on laboratory values and medical visits since 1994. Patients with available HLA-B*5701 test and >3 HIV RNA measurements (or 3 before starting ART) were eligible for analyses. HLA-B*5701 allele was determined by PCR-SSP (Inno-Train HLA-B Ready Gene B57 PCR-SSP). Non-parametric tests were used for group comparison and Kaplan-Meier survival analyses to estimate the probability of achieving HIV RNA <50 copies/ml.

**Results**

In total 440 patients were included and 31 (7 %) were HLA-B*5701(+). 341 (77.5 %) patients started ART, 95.3 % achieved HIV RNA < 50. Baseline, second and third HIV RNA values were lower in the HLA-B*5701 positive. Baseline median values were 3.6 vs 4.16 log copies/ml; p = 0.008, second HIV RNA median values were 3.45 vs 4.11 log copies/ml, and third median values were 3.82 vs 4.21 log copies/ml. CD4 baseline count was not statistically significant in the compared groups (553 cells/ul HLA-B*5701 positive, and 509 cells/ul HLA-B*5701 negative p = 0.08). Median time to starting ART did not differ significantly between HLA(+) and (-) group (2.83 and 2.34 years; p = 0.16), but median time to achieving HIV RNA <50 did (0.14 and 0.34 years; p = 0.008). Median parameters showing HIV positive patients' general condition, like hemoglobin and CRP were the same in both groups. Median hemoglobin was 14.8 mg% (p = 0.81) in both HLA-B*5701 positive and negative. Median CRP was 5 mg/l in both HLA-B*5701 (+) and (-) (p = 0.02). Median D-dimers did not differ significantly between two groups (236 ug/l HLA-B*5701 (+) and 220 ug/l HLA-B*5701 (-) p = 0.91).

**Conclusions**

We present that patients with HLA-B*5701(+) have both lower viral set point before starting treatment and shorter time to achieving undetectable HIV RNA after starting ART. HLA-B*5701 may play an additive role to HIV suppression in patients starting ARV.

#### O22 Increase in the numbers of non-B subtypes and potential recombinant forms circulating among Slovenian MSM in the recent years

##### Maja M. Lunar^1^, Jana Mlakar^1^, Mario Poljak^1^

###### ^1^Institute of Microbiology and Immunology, Faculty of Medicine, University of Ljubljana, Ljubljana, Slovenia

####### **Correspondence:** Maja M. Lunar (maja.lunar@mf.uni-lj.si) – Institute of Microbiology and Immunology, Faculty of Medicine, University of Ljubljana, Ljubljana, Slovenia

**Background**

In Slovenia as in many Western countries subtype B is still a predominant subtype and was historically correlated with the epidemic among men who have sex with men (MSM). In the recent years, several reports demonstrating an increasing prevalence of non-B subtypes have been published. The majority of infections with non-B subtypes were linked with the heterosexual mode of transmission in previous Slovenian studies, thus the aim of this study was to investigate whether non-B subtypes are becoming more prevalent also among MSM in Slovenia.

**Methods**

Between the years 2000-2014 a total of 520 persons were diagnosed with HIV-1 infection in Slovenia. Under the study of HIV-1 transmitted drug resistance filled questionnaires were obtained for 440 patients and amongst 326 patients reported homosexual contact as the most probable mode of HIV acquisition. Subsequently, partial pol sequences were obtained for 252 MSM patients which were included in the present study. Sequences were analyzed using the following automatic subtyping tools: REGA 2.0, REGA 3.0, COMET HIV-1 1.0, jpHMM and SCUEAL. Sequences that gave divergent subtyping results were considered to be potential recombinant forms. Temporal trend of the proportion of non-B and potential recombinant sequences (both combined termed as “non-pure subtype B” sequences) was evaluated with Fisher exact test used for the assessment of statistical significance.

**Results**

All five subtyping tools gave concordant subtyping results to 230/252 (91.3 %) of sequences. Pure subtype B was assigned to 226/252 (89.7 %) sequences and subtype A, subtype C, subtype F and CRF01_AE were determined in one patient each (0.4 %). The remaining 22/252 (8.7 %) sequences yielded divergent results with at least one of the subtyping tools, an indication to a possible recombination event in the past. An increase in the proportion of “non-pure subtype B” HIV-1 variants was noted over the years with 0 % (95 % confidence interval (CI): 0-16 %), 5 % (95 % CI: 1-15 %), 4 % (95 % CI: 0-13 %), 11 % (95 % CI: 4-21 %) and 25 % (95 % CI: 15-39 %) determined in the years 2000-2002, 2003-2005, 2006-2008, 2009-2011 and 2012-2014, respectively. The marked increase was on account of an increasing number of potential recombinant sequences with subtype B as one of the founder subtypes, since this subtype was identified in 21/22 of divergent sequences. The remaining divergent sequence was a complex recombinant containing subtypes D and G. Additionally, all 4 pure non-B subtyped sequences were determined in patients diagnosed in the last two years (2013-2014). The obtained results indicate that subtypes other than B are entering the HIV-1 MSM epidemic in Slovenia, making recombination events among different subtypes more plausible.

**Conclusion**

A marked increase in the numbers of non-B subtypes and potential recombinant forms was observed among MSM in Slovenia in the recent years. This finding indicates that subtypes other than B are entering the MSM epidemic in our region bearing diagnostic and clinical significance.

#### O23 Genotyping intrahost polymorphisms in hepatitis C virus E2 protein associated with resistance to antibody neutralization

##### Leontina Bănică^1^, Eliza Martin^2^, Valeriu Gheorghiță^3^, Andrei Petrescu^2^, Dan Oțelea^1^, Costin-Ioan Popescu^2^, Simona Paraschiv^1^

###### ^1^Molecular Diagnostic Laboratory, National Institute for Infectious Diseases “Prof. Dr. Matei Balș”, Bucharest, Romania; ^2^Institute of Biochemistry of the Romanian Academy, Bucharest, Romania; ^3^Clinical Department, National Institute for Infectious Diseases “Prof. Dr. Matei Balș”, Bucharest, Romania

####### **Correspondence:** Leontina Bănică (leo_mirela@yahoo.com) – Molecular Diagnostic Laboratory, National Institute for Infectious Diseases “Prof. Dr. Matei Balș”, Bucharest, Romania

**Background**

Hepatitis C virus (HCV) represents a serious global health problem with 170 million people infected worldwide. Despite the major advance in the HCV standard of care there is need of a vaccine for both prophylactic and therapeutic purposes. The major challenge in vaccine development is represented by the genetic diversity of the HCV envelope protein which may be overcome by a proper cellular and humoral immune response. In chronic infection the antibodies generated are unable to neutralize the virus due to multiple immune evasion mechanisms among which neutralizing resistance polymorphisms. Herein, we present a method to evaluate intrahost variability in HCV E1E2 region and to identify polymorphisms possibly associated with resistance to antibody neutralization.

**Methods**

Blood samples from two HCV genotype 1b infected patients with different stages of fibrosis were collected. Viral RNA was extracted from patient sera and following reverse transcription, the E1E2 region of HCV genome was specifically amplified in 2 rounds PCR reaction. The amplicon was cloned in the mammalian expression vector pcDNA3.1/V5-His TOPO TA following the manufacturer instructions. For each clone, the E1E2 region was sequenced using 6 different primers and 3500 ABI instrument. Seqscape software was used to assemble and generate the consensus sequences for each clone and BioEdit and FastTree softwares were further used to analyse the genetic evolution and the intra and interhost viral diversity. Structural modeling and sequence analysis bioinformatic techniques were used to select E1E2 envelopes for further analysis. The functionality and the sensitivity to neutralization of the selected HCV envelopes were evaluated using the HCV pseudotyping system.

**Results**

Ten complete ORFs of HCV E1E2 envelope glycoproteins for each patient were cloned. High intra-host variability was observed for both patients, one of them (F1 fibrosis stage patient) presenting two distinct viral sub-populations, as indicated by the phylogenetic analysis. Further, we have focused on aminoacid positions previously reported to be involved in resistance to antibody neutralization. The residues were mapped on a 3D structural model of E2 glycoprotein based on the crystal structure of the core domain of HCV E2. Relying on the bioinformatic analysis, we selected variants which are likely to be resistant to antibody neutralization. Further, we tested the functionality and sensitivity to neutralization of the HCV E1E2 envelopes. We are discussing possible mechanisms of resistance to antibody neutralization.

**Conclusions**

Mapping the intrahost diversity of HCV E1E2 glycoproteins will contribute to the understanding of the molecular mechanism governing resistance to neutralizing antibodies and vaccine design.

Acknowledgments

This study was supported by ANCSI grant EEA-JRP-RO-NO-2013-1-0022, Grant GreenVac and by POSCCE Program, CRCBABI Project (642/2014).

#### O24 Genotyping of HCV NS3 protease inhibitors resistance and phenotyping of rare double resistance mutations in HCV cell culture system

##### Emil Neaga^1^, Vlaicu Ovidiu^1^, Andrei Juncu^2^, Leontina Bănică^1^, Simona Paraschiv^1^, Dan Oțelea^1^, Costin-Ioan Popescu^2^

###### ^1^National Institute for Infectious Disease “Prof. Dr. Matei Balș”, Bucharest, Romania; ^2^Institute of Biochemistry of the Romanian Academy, Bucharest, Romania

####### **Correspondence:** Costin-Ioan Popescu (pop@biochim.ro) – National Institute for Infectious Disease “Prof. Dr. Matei Balș”, Bucharest, Romania

**Background**

For human immunodeficiency virus (HIV) infected patients, the hepatitis C virus (HCV) co-infection represents a serious problem because HIV-HCV co-infection represents the major cause of liver related morbidity among this group. Recently, the HCV standard of care was profoundly upgraded with direct acting antivirals (DAA) which are administrated alone or in combination with pegylated interferon alpha and ribavirin. Although the efficacy of the new therapies is quite high, drug resistance and viral genetic diversity are still issues to be investigated.

**Methods**

For our study, we selected sera from genotype 1b patients who failed to achieve sustained virological response (SVR) after treatment with triple therapy consisting of first or second generation HCV NS3 protease inhibitors (PI), alpha interferon and ribavirin. The resistance was genotyped by population sequencing in the HCV NS3 serine protease region. To phenotype the resistance, we used two recombinant viruses: genotype 1a TNcc cell culture adapted virus and 5’UTRNS5A1a/2a chimeric virus. The replication and secretion capacity were evaluated by anti-HCV core ELISA assay and infectivity was assessed by titration using the foci forming unit assay.

**Results**

Interestingly, we found a rare combination of double lower level resistance mutations (T54S, R155K, A156S) associated with elevated viral loads suggesting a minimal effect on the viral fitness. We are discussing possible compensation mechanisms between HCV NS3 PI resistance mutations.

**Conclusion**

Phenotyping of drug resistance in clinical relevant cell culture systems will contribute to our understanding of drug resistance mechanisms in the emerging interferon-free HCV therapy era.

Acknowledgments

The project was supported by Grant POSDRU/1591/1.5/S/135760-CERO and by POSCCE Program, CRCBABI Project (642/2014).

## ORAL PRESENTATIONS

### Session: NeuroHIV

#### O25 Employment status controls the relationship between neurocognitive impairment and depression in a cohort of young HIV-infected adults since childhood

##### Adrian Luca^1^, Florin Lazăr^2^, Anca Elena Luca^3^, Luminița Ene^3^, Cristian Achim^4^

###### ^1^Faculty of Psychology and Education Sciences, University of Bucharest, Bucharest, Romania; ^2^Faculty of Sociology and Social Work, University of Bucharest, Bucharest, Romania; ^3^“Dr. Victor Babeș” Hospital for Infectious and Tropical Diseases, Bucharest, Romania; ^4^HIV Neurobehavioral Research Center, University of California, San Diego, USA

####### **Correspondence:** Adrian Luca (adrianluca@gmail.com) – Faculty of Psychology and Education Sciences, University of Bucharest, Bucharest, Romania

**Background**

Children with parenterally acquired HIV infection in childhood in the early ’90, living with chronic HIV for over 25 years might have HIV-associated neurocognitive impairment (NCI). Moreover they face challenges regarding everyday functionality, depression, social interactions and low employment that we aimed to investigate in relationship with their NCI.

**Methods**

A clinic-based survey among 233 HIV+ participants with a median age of 24.3 years, 47.6 % males infected since childhood was carried out between 2012-2015 in an infectious diseases hospital in Bucharest, Romania. Patients’ functioning was assessed using two self-report questionnaires that report level of competence in performing instrumental and basic tasks of everyday life (Activities of Daily Living (ADL) (α = 0.67)) and subjective cognitive complaints (Patient’s Assessment of Own Functioning Inventory (PAOFI) (α = 0.93)), NCI was investigated with a neurocognitive battery assessing 7 domains known to be most affected by HIV with a composite global deficit score (GDS). Other measures included: Beck Depression Inventory II (BDI) (α = 0.89), a performance-based skills assessment (UPSA, α = 0.65) together with data on their job status, and social interactions (number of daily interactions outside family). The present analysis is focusing on the association between depression and NCI.

**Results**

Depression is positively associated with impairment (GDS) (sig. = 0.026), daily living dysfunctionalities (ADL) (sig. = 0.000), more cognitive psychological complaints (PAOFI) (sig. = 0.000) and fewer social interactions (sig. = 0.006). Moreover, having a job controls the relationship between depression and cognitive functioning (GDS), zero-order correlation (r = 0.145, sig. = 0.026) and correlation with control for employment status is r = 0.119, sig = 0.070. Unemployed depressive persons demonstrate a cognitive deficit (mGDS = 0.63, F = 2.899, sig. = 0.036, η2 = 0.037), a decline in daily functioning (mADL = 4.44, F = 9.329, sig. = 0.000, η2 = 0.109), more cognitive complaints (mPAOFI = 5.19, F = 18.922, sig = 0.000, η2 = 0.199) and the fewest social interactions (mSI = 7.33, F = 5.323, sig. = 0.001, η2 = 0.065) in comparison with employed depressed persons or not-depressed ones. The employed people (with/without depression) perform better at UPSA (mD + =81.81, mD- = 81.05). Being employed without depression symptoms is associated with better cognitive abilities (mGDS = 0.32), better functioning (mADL = 2.19), less cognitive complaints (mPAOFI = 0.64) and more social interactions (mSI = 16.48). Being unemployed without depression is associated with a mild cognitive deficit (mGDS = 0.50).

**Conclusion**

Being employed supports HIV infected people to unravel at practical activities, have less cognitive complaints, be more functional in everyday tasks, less depressed, with more social contacts and a better cognitive functioning. Having a job increases person’s functioning by putting cognitive abilities in action.

Acknowledgement

NIH-funded grant R01MH094159-01 “Long term effects of chronic HIV infection on the developing brain”.

#### O26 Predictors of survival in parenterally-infected HIV positive children and youth diagnosed with progressive multifocal leukoencephalopathy

##### Cosmina Gingăraş^1^, Ștefan Adrian Anton^1^, Roxana Rădoi^1^, Simona Tetradov^1^, Grațiela Țârdei^2^, Maria Nica^3^, Razvan Alexandru Capşa^4,5^, Cristian L. Achim^6^, Cristiana Oprea^1,7^, Luminița Ene^1^

###### ^1^5^th^ Clinical Department of Infectious Diseases (HIV Department), “Dr. Victor Babeș” Hospital for Infectious and Tropical Diseases, Bucharest, Romania; ^2^Virology, Immunology, Molecular Biology Department, “Dr. Victor Babeș” Hospital for Infectious and Tropical Diseases, Bucharest, Romania; ^3^Microbiology Department, “Dr. Victor Babeș” Hospital for Infectious and Tropical Diseases, Bucharest, Romania; ^4^“Dr. Victor Babeș” Medical Center for Diagnosis and Treatment, Bucharest, Romania; ^5^Department of Radiology, Fundeni Institute, Bucharest, Romania; ^6^UC San Diego, Departments of Psychiatry and Pathology, La Jolla, CA, USA; ^7^Carol Davila University of Medicine and Pharmacy, Bucharest, Romania

####### **Correspondence:** Cosmina Gingăraş (cosminagingaras@gmail.com) – 5^th^ Clinical Department of Infectious Diseases (HIV Department), “Dr. Victor Babeș” Hospital for Infectious and Tropical Diseases, Bucharest, Romania

**Background**

Progressive multifocal leukoencephalopathy (PML) is a severe opportunistic infection of the central nervous system (CNS), with high mortality and rare spontaneous remissions, even in the setting of highly active antiretroviral therapy (HAART). We aimed to investigate the prevalence, outcomes and factors associated with survival in patients with PML who belong to the cohort of children parenterally-infected with HIV at the end of the 1980’s in Romania.

**Methods**

Retrospective chart review of all HIV positive patients parenterally-infected and diagnosed with PML between 1990-2016 in a tertiary care center. PML diagnosis was established based on clinical symptoms, neuroimaging, cerebrospinal fluid (CSF) findings and neurohistological examination. We performed summary statistics of demographic, clinical and laboratory collected data. Logistic regression was used to determine predictors of survival at several time points. Data were analyzed using STATA 11 (College Station, TX, USA).

**Results**

A total of 46 patients were diagnosed with PML, 45 % before 2006. Half of the patients were male and the average age at diagnosis (baseline) was 17.8 years (range: 8.2-27.7) with a bimodal distribution, with peaks at 11 and 22 years. The median baseline CD4 count was 32.5 cells/uL (IQR: 10-90 cells/uL); median HIV viral load (VL): 5.44 log copies/mL (IQR: 4.3-5.8); median HIV VL in CSF: 3.5 log copies/mL (IQR: 2.6-4.2). Twenty-six (58 %) cases were confirmed by JCV PCR. MRI lesions were supratentorial in 34.9 % of patients, subtentorial in 25.6 %, and mixed in 39.5 %. Seventeen patients (37 %) were alive at the end of the study. Median survival time was 16 months (range: one month-15.8 years). Survivors were older at diagnosis and had better baseline clinical, immunological and virological parameters. Those who died had a median survival of 2.8 months (IQR: 1.5-13.6) after PML diagnosis and a preponderance of subtentorial or mixed lesions on MRI. The strongest predictors of survival at the moment of diagnosis were baseline CD4 count and VL. Univariate logistic regression analysis identified age at PML diagnosis, baseline viral load and use of cART before diagnosis as strong predictors of survival beyond one year from diagnosis, and a marginal significance for use of cART with greater CNS penetrability scores, but none of these factors maintained significance in multivariate models.

**Conclusions**

In our study period, PML was almost as prevalent after the introduction of HAART as it was before. However, being exposed to cART and having a lower baseline viral load and higher CD4 count were associated with long-term survival.

#### O27 Neurocognitive and brain functioning in HIV-infected young MSM treated with cART

##### Bogna Szymańska^1^, Natalia Gawron^2^, Agnieszka Pluta^3^, Emilia Łojek^2^, Ewa Firląg – Burkacka^1^, Andrzej Horban^4^, Robert Bornstein^5^, et HARMONIA3 Study Group

###### ^1^Outpatients Clinic, Hospital for Infectious Diseases in Warsaw, Poland; ^2^The Faculty of Psychology, University of Warsaw, Warsaw, Poland; ^3^World Hearing Center, Institute of Physiology and Pathology of Hearing, Nadarzyn, Poland; ^4^Medical University of Warsaw, Warsaw, Poland; ^5^Department of Psychiatry and Behavioral Health, Ohio State University, Columbus, OH, USA

####### **Correspondence:** Bogna Szymańska (bogna.szymanska@gmail.com) – Outpatients Clinic, Hospital for Infectious Diseases in Warsaw, Poland

**Background**

There is growing evidence that despite successful antiretroviral treatment, neurocognitive and brain dysfunctions can be still observable in HIV-infected individuals. The aim of this study was to investigate neurocognitive and brain functioning in young HIV(+) MSM, treated with cART, with undetectable HIV1-RNA in serum.

**Methods**

Fifty HIV(+) individuals and 42 HIV(-) controls aged 20-40, compared on socio-demographic variables were analyzed in this study. The characteristics of HIV(+) group were as follows: duration of HIV infection M = 3.7 years, SD = 3.2; CD4 + nadir M = 311 cells/μL, SD = 125; CD4+ current M = 599 cells/μL, SD = 193; duration of cART M = 2.9 years, SD = 2.9; 54 % were diagnosed with STD (syphilis) in the past. They were treated with ARV for a minimum 10 months. We analyzed 3 types of cART regimens: 2NRTI + PI/r, 2NRTI + NNRTI and other regimens. Out of both groups, 30 HIV(+) and 27 HIV(-) individuals were examined on the n-back task in 3-Tesla MRI Scanner. The participants were performed a battery of neuropsychological tests and psychological questionnaires.

**Results**

Despite preserved abilities on general mental status, intelligence, reasoning, language, psychomotor skills and mood, HIV(+) individuals showed significant impairment comparing with the controls in the domains of working memory, psychomotor speed, attention and executive function: VMS Forward (p < 0.05) and Backward (p < 0.002), WAIS Digit Span (p < 0.002), RFFT (p < 0.002), WCST (p < 0.05). We have not observed differences between cART regiments. In the psychological questionnaires HIV(+)MSM exposed better coping strategy (KPD) (p < 0.05), greater social activity (SAQ) (p < 0.004), social support (SSQ) (p < 0.05) and stronger masculine features than controls (PAQ) (p < 0.001).

Although similarities of the results in the n-back task, the patterns of brain activation during that task in fMRI were different in both groups. Bilateral activation was observed in the middle frontal gyrus, insula, putamen, thalamus and the parietal lobule in both groups. Whereas the controls showed increased activation in superior parietal lobule in right hemisphere in comparison with HIV(+) individuals.

**Conclusion**

Young HIV(+) MSM presented selected deficits in working memory, psychomotor abilities, attention and executive domains although good psychological factors. They also showed slight changes in brain activity during the fMRI task. Thus despite effective cART HIV(+) MSM manifested changes in the neurocognitive, psychological and brain functioning comparing with the controls.

Acknowledgement

1. Hospital for Infectious Diseases in Warsaw, Poland.

2. The Faculty of Psychology, University of Warsaw, Poland.

#### O28 Clinical value of RT-PCR detection of *Toxoplasma gondii* DNA in cerebrospinal fluid

##### Olivia Burcoș^1^, Simona Manuela Erscoiu^2^, Filofteia Bănicioiu Cojanu^1^, Andreea Toderan^1^, Maria Nica^2^, Ionuț Cristian Popa^1^, Emanoil Ceaușu^2^, Petre Iacob Calistru^2^

###### ^1^Clinical Hospital for Infectious and Tropical Diseases “Dr. Victor Babeș”, Bucharest, Romania; ^2^Carol Davila University of Medicine and Pharmacy, Bucharest, Romania

####### **Correspondence:** Olivia Burcoș (olivia_burcos@yahoo.com) – Clinical Hospital for Infectious and Tropical Diseases “Dr. Victor Babeș”, Bucharest, Romania

**Background**

*Toxoplasma gondii* encephalitis (TE) in HIV infected patients is a severe and frequent opportunistic infection, even in highly active antiretroviral era. Magnetic resonance imaging (MRI) is often suggestive, but sometimes the differential diagnosis of cerebral mass can be very difficult. Sensitivity of the PCR in CSF in the literature ranges between 33-100 % and specificity between 94-100 %. We calculated these constants in our clinic.

**Method**

Probable toxoplasmosis was defined as neurological symptoms appeared in HIV patients, with typical MRI and favorable outcome under etiologic therapy. Data were collected retrospectively from January 2012 to January 2016. A control group consisted of HIV patients with no personal history of TE, confirmed with other central nervous system conditions (HIV encephalopathy, progressive multifocal leukoencephalopathy, cryptococcal or tuberculous meningitis, cerebral lymphoma-confirmed by RT-PCR of JC virus, *Mycobacterium* or *Cryptococcus* cultures, pathologic examination), and no additional lesions suggestive for TE coinfection. Lumbar puncture was performed in the first day after the diagnosis (media 0.47 days). For RT-PCR technique was used nucleic acid extraction kit straight from CSF, Master Pure Complete DNA&RNA Purification Kit (Illumina), commercial primer Primerdesign genesig Kit for *Toxoplasma gondii* genomes and Light Scanner 32 Instrument (Idaho Technologies, USA). Chemiluminescense method was used for serologic tests.

For RT-PCR technique was used nucleic acid extraction kit straight from EDTA blood, Master Pure Complete DNA&RNA Purification Kit/Epicentre Biotechnologies, commercial primer Primer Design UK and Light Scanner 32/Idaho Technologies.

**Results**

RT-PCR for *Toxoplasma* was performed in the CSF of 23 patients (out 33 suspected cases of TE). Only in 3 cases the RT-PCR was negative (one of them had a positive RT-PCR in the brain biopsy). 7 patients with compatible symptoms and characteristic MRI, with positive RT-PCR for *Toxoplasma* in CSF, died of severe disease and were excluded from the study. Serologic tests for *Toxoplasma* (IgG) in the plasma were positive (18 of 23 tested) and all serologic tests in CSF were negative (10 out of 23 tested). 17 patients with other neurologic conditions were selected for the control group. All had negative RT-PCR in the CSF. The calculated sensitivity of the RT-PCR for *Toxoplasma gondii* in CSF was 84.2 %, 100 % specificity, positive predictive value 100 % and negative predictive value 85 %.

**Conclusion**

RT-PCR for *Toxoplasma gondii* in CSF is a good diagnostic method compared to CSF serologic tests, and relatively non invasive compared to brain biopsy.

#### O29 Characteristics of sleep disorders in Romanian adults infected with human immunodeficiency virus

##### Manuela Arbune^1^, Mirela Alexandrache^2^, Anca-Adriana Arbune^3^, Doina-Carina Voinescu^1^

###### ^1^Clinical Department, “Dunărea de Jos” University Galați, Galați, Romania; ^2^Infectious Diseases Clinical Hospital Iași, Iași, Romania; ^3^Clinical Department 6, Carol Davila University of Medicine and Pharmacy, Bucharest, Romania

####### **Correspondence:** Manuela Arbune (manuela.arbune@ugal.ro) – Clinical Department, “Dunărea de Jos” University Galați, Galați, Romania

**Background**

Sleep disorders are common symptoms in chronic diseases and contribute to decreased quality of life. Increased frequency of sleep disorders in patients with HIV infection was associated with reduced slow-wave sleep, but the influence of immunosuppression, opportunistic diseases or antiretroviral drugs (Efavirenz) is controversial. The aim of the study is to characterize the sleep problems in a group of Romanian HIV seropositive patients.

**Methods**

A cross sectional study assessed the sleep disorders by self-report of Epworth sleep questionnaire and Pittsburgh Sleep Quality Index with seven domains: subjective sleep quality, sleep latency, sleep duration, habitual sleep efficiency, sleep disturbances, use of sleeping medication and daytime dysfunction. Patients with PSQI ≥5 were diagnosed with poor sleep quality. Demographic data, antiretroviral therapy, Beck-II depression score, CD4 cell count were collected from medical records. Exclusion criteria were illiteracy and severe or moderate depression (Beck-II score >18). Categorical variables as gender, marital status category, medication group category, CD4 cell count category below or above 200 cells/cmm, Beck score bellow or above 13, are presented as frequencies (%) and group differences compared using Chi^2^ test, with the significance level <0.05.

**Results**

The characteristics of patients were age 30.66 ± 6.86, 50.6 % males, 56.6 % urban living area, 49.3 % married/stable relationship, 16.8 % employed. Most patients had immunity LCD4 > 500 cells/cmm (56.2 %). Risk factors of sleep disorder were: smoking (46.9 %), hypertension (21.69 %), obesity (12.05 %), depression (33.73 % scoring Beck-II between 13 and 18), snoring (36.14 %). Epworth score was 5.20 ± 4.34 while 26.25 % patients with values >7 have the risk to fall asleep during the daytime. The mean PSQI value was 4.89 ± 2.89 and 49.4 % patients associate sleep dysfunctions. Sleep disturbance and daytime dysfunctions were the main disorders. The PSQI global score was correlated with age and level of immunity. Snoring was correlated with age, hypertension and obesity. Patients with Efavirenz treatment have reported frequently daytime dysfunction and sleeping pills use.

**Conclusions**

Practically half of adult patients with HIV infection under age 50 have sleep problems and negative impact on daily functioning. The strategy to prevent and control those disorders in the coming years should consider the effective and less neurotoxicity antiretroviral therapy, the psychological support, hypertension and obesity care.

#### O30 Diagnosing neuroHIV: the rift between clinicians and pathologists

##### Ioan-Alexandru Diaconu^1†^, Laurențiu Stratan^1†^, Victoria Aramă^1,2^, Luciana Nichita^3^, Alexandra Diaconu^4^, Anca Negru^1,2^, Alina Orfanu^1,2^, Anca Leuștean^1^, Daniela Adriana Ion^1,2^

###### ^1^National Institute for Infectious Diseases “Prof. Dr. Matei Balș”, Bucharest, Romania; ^2^Carol Davila University of Medicine and Pharmacy, Bucharest, Romania; ^3^Colentina Clinical Hospital, Bucharest, Romania; ^4^“Marius Nasta” Pneumophtisiology Institute, Bucharest, Romania

####### **Correspondence:** Ioan-Alexandru Diaconu (diaconuia@yahoo.com) – National Institute for Infectious Diseases “Prof. Dr. Matei Balș”, Bucharest, Romania

^†^The authors had equal participation.

**Background**

There has been little research conducted in Romania regarding the neurological complications that arise from infection with the human immunodeficiency virus 1 (HIV-1), from a histopathologist’s point of view. Our study aims to create an extensive record of HIV-induced central nervous system involvement through post-mortem histopathological examination, immunohistochemistry and immunofluorescence in correlation with clinical diagnoses issued ante mortem.

**Methods**

The investigated material consisted entirely of human brain tissue samples collected during some of the autopsies conducted in the Pathology Department of Colentina Clinical Hospital, Bucharest, over a time span longer than 22 years (1993 - 2015) on 39 HIV+ patients hospitalized in the National Institute for Infectious Diseases “Prof. Dr. Matei Balș”. All cases were examined concurrently by two pathologists and quantified by common criteria in order to ensure a uniform assessment of the studied material.

**Results**

The group, composed of 39 HIV-positive patients, was created during a period of over 22 years and show marked heterogeneity, both in age, variety of diagnoses, treatment, as well as immunological status. From the group of 39 HIV-infected patients whose brain tissue was examined post-mortem, a third received antiretroviral therapy, 10 % of them for less than 5 months. The most common clinical diagnoses were bronchial pneumonia, oropharyngeal candidiasis, meningoencephalitis, pulmonary tuberculosis and HIV encephalopathy. The most common neurological histopathological diagnoses were hyperemia with edema, cryptococcal meningoencephalitis and HIV encephalopathy. In the non-cART branch, the top neurological histopathology diagnoses were hyperemia with edema, non-specific meningoencephalitis and cryptococcal meningoencephalitis. Hyperemia and edema was discovered in 20 % of the patients, with only a few being treated with antiretroviral therapy.

**Conclusions**

The results revealed that some of the clinical diagnoses, despite the use of modern methods of imaging and serological investigation, are inconsistent with the neurohistopathological findings, due to the heightened difficulty of the differential diagnosis. This situation is caused by the myriad of effects brought about by the HIV infection, illustrating the origin of a 60 % accuracy of diagnosis.

#### O31 A challenging neurological complication in a HIV-infected young woman with multiple opportunistic infections

##### Irina Ianache^1^, Cristiana Oprea^1,2^

###### ^1^5^th^ Clinical Department for Infectious Diseases (HIV Department), “Dr. Victor Babeș” Hospital for Infectious and Tropical Diseases, Bucharest, Romania; ^2^Carol Davila University of Medicine and Pharmacy, Bucharest, Romania

####### **Correspondence:** Irina Ianache (ianacheirina@gmail.com) – 5^th^ Clinical Department for Infectious Diseases (HIV Department), “Dr. Victor Babeș” Hospital for Infectious and Tropical Diseases, Bucharest, Romania

**Background**

Diagnosis of CNS immune reconstitution inflammatory syndrome (IRIS) is still an important challenge for the management of HIV-infected patients.

**Case description**

We present the case of a 25 year-old woman, who acquired HIV infection by parenteral mode (in the first years of life) and had a history of poor adherence to cART.

She was diagnosed with pulmonary tuberculosis (TB) with positive cultures in July 2013, but abandoned anti TB treatment shortly (after one month).

She was admitted in our department in January 2014, with fever, severe respiratory failure and left hemiparesis. The lab screen showed severe anemia, hypoxemia, increased LDH levels, severe immunosuppression (CD4 cell count - 9 /cmm) and high plasma viral load (VL) (5.92 log_10_ copies/mL). *Pneumocystis jirovecii* pneumonia (PCP) and pulmonary TB were diagnosed (chest X-rays and bacteriological exams). TB treatment and cotrimoxazole were initiated, with improvement of the respiratory symptoms but persistence of the neurological deficits. Brain MRI showed a hypo-intense round lesion on T1-sequence in the right parietal region, hyper-intense on T2, with ring enhancement and important edema, suggestive for cerebral toxoplasmosis. Cerebrospinal fluid (CSF) exam was unremarkable, with negative MTB cultures and negative PCR-DNA tests for both TB and toxoplasmosis. Serology for *Toxoplasma gondii* (IgG) was positive in plasma and CSF. After 6 weeks of cotrimoxazole and anti TB treatment and 3 weeks of cART (with integrase inhibitors) she was discharged with favorable clinical evolution and improved brain neuroimaging. Two months later, she was readmitted in our department with headache, generalized seizures, left hemiplegia and left facial palsy. We noticed an important increase in CD4 cell count (111/cmm) and a significant decrease in HIV-VL (2.5 log_10_ copies/mL). Brain MRI showed an increase in size and number of the cerebral lesions (parietal and frontal areas), with important edema, greater contrast enhancement in T1 and mass effect on the right lateral ventricle. Non-adherence to anti-toxoplasma treatment and resistance to cotrimoxazole were excluded. Paradoxical *Toxoplasma* IRIS was the most probable diagnosis in a patient with important immunological recovery, significant VL decrease after two months of cART, suggestive MRI imaging and increased inflammatory markers in plasma. Clinical evolution was favorable after continuing anti-toxoplasma treatment with pyrimethamine, sulphadiazine combined with corticotherapy with regression in size and number of the cerebral lesions.

**Conclusion**

We describe a particular case of paradoxical toxoplasma IRIS in a woman with multiple concomitant opportunistic infections.

**Consent**

Written informed consent was obtained from the patient for publication of this Case report and any accompanying images. A copy of the written consent is available for review by the Editor of this journal.

#### O32 Brain abscess with uncertain etiology in a late-presenter HIV infected patient

##### Anca Leuștean^1^, Cristina Popescu^1,2^, Alina Orfanu^1,2^, Anca Negru^1,2^, Remulus Catana^1,2^, Cristina Murariu^1^, Ioan-Alexandru Diaconu^1,2^, Mihaela Rădulescu^1,2^, Cătălin Tilișcan^1,2^, Victoria Aramă^1,2^

###### ^1^National Institute for Infectious Diseases “Prof. Dr. Matei Balș”, Bucharest, Romania; ^2^Carol Davila University of Medicine and Pharmacy, Bucharest, Romania

####### **Correspondence:** Anca Leuștean (anca_leustean@yahoo.com) – National Institute for Infectious Diseases “Prof. Dr. Matei Balș”, Bucharest, Romania

**Background**

CNS infection in HIV patients may occur during any stage but most frequent if CD4 count is below 200 cells/cmm. The onset of this infection can be acute, subacute or chronic and the most common etiology could be: toxoplasmosis (T), cryptococcal infection, primary CNS lymphoma (PL), progressive multifocal leukoencephalopathy (PML), AIDS dementia complex or CMV infection.

**Case presentation**

A 19-year old female was admitted in our clinic to investigate and stage a recently discovered HIV infection. She first presented to another hospital for fever, fainting, headache, paraesthesia in her lower limbs and fatigue. The laboratory results showed pancytopenia and a positive test for HIV. On admission in our clinic, the patient was afebrile, without neck stiffness but with positive Kernig sign and a slight facial asymmetry. The laboratory results confirmed an HIV infection with a CD4 count of 42 cells/cmm, an HIV viral load of 295807 copies/ml, positive IgG and negative IgM for *Toxoplasma gondii* and Cytomegalovirus, negative Quantiferon-TB, VDRL, IgM for Herpes Simplex and hepatitis viruses. We performed a lumbar puncture which showed clear, hypertensive cerebrospinal fluid (CSF) with 15 cells/cmm, positive Pandy reaction, elevated protein level (129 mg %), glucose level 26 mg % (serum glucose level 68 mg %), normal lactic acid, negative *Cryptoccocus*, negative PCR for *Mycobacterium* and an HIV viral load in CSF of 347752 copies/ml. The CT scan showed a low-density area of 2.8/2.1 cm, with peripheral contrast and important surrounding edema, localized in the capsule-lenticular and left parietal structures, with a mass effect and deviation of the median line structures to the right by 6 mm, interpreted as a cerebral abscess. The CT scan was repeated with cerebral perfusion to rule out a cerebral lymphoma. The possible etiologies taken into consideration were: pyogenic brain abscess, cerebral toxoplasmosis, cerebral tuberculosis, Herpes Simplex Virus infection and cerebral lymphoma. The antimicrobial treatment consisted in ceftriaxone (2gx2/day), vancomycin (1gx2/day) for 14 days, cotrimoxazole 12 tablets/day, intravenous acyclovir 500mgx2/day and also MAC prophylaxis with clarithromycin. After 7 days of treatment, the CT scan showed an improvement of the cerebral lesions. The treatment with cotrimoxazole 12 tablets/day was stopped after 3 weeks because of the aggravation of her pancytopenia. Given the high HIV viral load in the CSF and the low CD4 count, with a negative HLA-B5701, the antiretroviral therapy (ART) was initiated with Efavirenz and Abacavir/3TC, without any signs of Immune Reconstruction Inflammatory Syndrome (IRIS). The third CT scan (2 months later) showed a 6 mm low-density sechelar lesion lateral of the caudate nucleus, without any mass effect. After another two months, the CT scan showed the same sechelar lesion. Also, the hemogram was normal and the CD4 count was of 426 cells/cmm. The lumbar puncture was repeated, with a slightly hypertensive CSF, slightly elevated protein level and normal glucose and lactic acid levels and an HIV viral load in the CSF of 88 copies/mL.

**Conclusion**

Although in HIV infected patients with cerebral abscess toxoplasmosis is the most frequent etiology, pyogenic abscess must be taken into account. A less aggressive ART as a first regimen can sometimes avoid IRIS in a patient with very low CD4 count.

**Consent**

Written informed consent was obtained from the patient for publication of this Case report and any accompanying images. A copy of the written consent is available for review by the Editor of this journal.

#### O33 Cerebral toxoplasmosis and left crural monoparesis with fatal evolution in a noncompliant patient with AIDS C3

##### Iosif Marincu^1^, Patricia Poptelecan^1^, Valeria Bică^1^, Florin Lazăr^2^, Livius Tirnea^1^

###### ^1^Department of Infectious Diseases, Pneumology, Epidemiology and Parasitology, “Victor Babeș” University of Medicine and Pharmacy, Timișoara, Romania; ^2^Faculty of Sociology and Social Work, University of Bucharest, Bucharest, Romania

####### **Correspondence:** Iosif Marincu (imarincu@umft.ro) – Department of Infectious Diseases, Pneumology, Epidemiology and Parasitology, “Victor Babeș” University of Medicine and Pharmacy, Timișoara, Romania

**Background**

Cerebral toxoplasmosis remains the most frequent neurological opportunistic infection in patients with HIV/AIDS. We present a clinical case of cerebral toxoplasmosis with fatal evolution in an unadherent patient with C3 stage AIDS.

**Case report**

A 31 years old female patient coming from a rural area, diagnosed with HIV Infection 2 years before, was hospitalized in the Clinic of Infectious Diseases Timișoara, accusing: dysphagia for solids and liquids, poor motor function, left hemiparesis and left facial paralysis, paleness of skin, fatigue, loss of appetite. The patient is noncompliant at the antiretroviral (ARV) therapy and has a reduced level of medical education. The positive diagnosis was based on clinical elements (the altered general condition, conscious, with left facial paralysis, pallor, lingual candidiasis, etc.), the biological samples (blood cell identification, erythrocyte sedimentation rate (ESR), glucose, C-reactive protein (CRP), blood cultures, CD4 count, viral load (VL), *Toxoplasma* IgG and IgM antibodies, etc.) and paraclinical investigations (chest radiography, brain computed tomography (CT) scan, abdominal ultrasound). Biological samples were analyzed in the hospital laboratory.

From the blood tests we highlight: total white blood cell 2930/μL, Hb 10.5 g/dl, CRP 95.69 mg/L, ESR 85 mm/1st hour, *Toxoplasma* IgG Ab 282 U/mL, CD4 count 31 cells/μL, VL 590381 copies/mL, cultures from the lingual swabs showed Candida albicans >10 CFU. Serum electrolytes, liver and kidney function tests were normal. CT scan of the brain showed: hypodense areas, a fluid density image with a diameter of 2.2/1 cm in core lentiform left nuclei, a 1.5/2.2 cm image in the right lentiform nuclei in the right saphenous capsule (1/0.5 cm). Neurological exam revealed: conscious patient, cooperant, without neck stiffness, walking is possible with support, crural monoparesis, left Babinski reflex-plantar cutaneous left without objectives sensitivity disorders, upper limb myoclonus as predominantly left postural action, anisocoric mild cranial nerves (right > left). Diagnosis: crural monoparesis left upper limb myoclonus, right cerebral toxoplasmosis. Despite the complex therapy instituted (clindamycin, pyrimethamine, mannitol, glucose 10 %, NaCl 0.9 %, fluconazole, levetiracetam, dexamethasone, ARV, etc.), overall condition was aggravated, the patient becomes uncooperative, with progressive dysphagia for solids and liquids, with periods of unconsciousness and finally with unfavorable evolution.

**Conclusion**

Late diagnosis of opportunistic brain infections in HIV-infected and nonadherent patients, despite a complex rescue therapy may be associated frequently with unfavorable evolution of these patients. It is required a rigorous therapeutic clinical monitoring, along with a repeated psychological counseling of patients with ARV treatment.

**Consent**

Written informed consent was obtained from the patient for publication of this Case report and any accompanying images. A copy of the written consent is available for review by the Editor of this journal.

#### O34 Opportunistic infections still a problem in HIV-infected patients in cART era: a Romanian single center experience

##### Irina Ianache^1^, Roxana Rădoi^1^, Manuela Nica^2,4^, Grațiela Țârdei^3^, Luminița Ene^1^, Emanoil Ceaușu^1,4^, Petre Calistru^1,4^, Cristiana Oprea^1,4^

###### ^1^5th Clinical Department for Infectious Diseases (HIV Department), “Dr Victor Babeș” Hospital for Infectious and Tropical Diseases, Bucharest, Romania; ^2^Laboratory of Microbiology, “Dr Victor Babeș” Hospital for Infectious and Tropical Diseases, Bucharest, Romania; ^3^Laboratory of Virology, Immunology, Molecular Biology, “Dr Victor Babeș” Hospital for Infectious and Tropical Diseases, Bucharest, Romania; ^4^Carol Davila University of Medicine and Pharmacy, Bucharest, Romania

####### **Correspondence:** Irina Ianache (ianacheirina@yahoo.ca) – 5th Clinical Department for Infectious Diseases (HIV Department), “Dr Victor Babeș” Hospital for Infectious and Tropical Diseases, Bucharest, Romania

**Background**

Despite significant improvement of AIDS-related morbidity and mortality in the cART era, the incidence of opportunistic infections (OIs) in HIV-infected patients in some middle income countries is still high. The aim of our study was to evaluate the incidence, demographics and outcome in patients diagnosed with OIs in a tertiary HIV care center.

**Methods**

Prospective study on HIV-infected patients diagnosed with OIs at “Victor Babeș” Hospital between January 2011 and December 2015. Diagnosis of OIs was definitive in cases with positive microbiological exams or presumptive (typical clinical, epidemiological, imaging techniques). Statistical comparison between OIs groups was performed using SPSS v 19.0, survival being compared using Kaplan Meier methods.

**Results**

Out of 5,728 person-years (PY), 250 HIV-infected patients were diagnosed with 314 OIs (incidence 54.8/1000 PY). The majority 169 (67.6 %) were males, with a median age at OIs diagnosis of 28 years (IQR: 25-37). The route of HIV transmission was: heterosexual 35.6 % (HSX), due to injecting drug use 32.0 % (IDUs), by parenteral mode during childhood 28.8 % (PI), MSM 2.0 % and from mother-to-child 1.6 %. The incidence of OIs increased from 3.5 % in 2011 to 7.0 % in 2015 (p = 0.0005).

The median CD4 cell count/cmm and HIV viral load (copies/mL) at OIs diagnosis was 41 (IQR: 16-136) and 5.19 log_10_ (IQR 3.91-5.67) respectively. In 111 cases (35.3 %) HIV and OI were diagnosed simultaneously and among 143 patients who developed OIs on cART, 84.6 % were non-adherent. The most common OIs were: tuberculosis (TB) 187 (59.5 %), progressive multifocal leukoencephalopathy (PML) 39 (12.4 %), *Pneumocystis jirovecii* pneumonia 23 (7.3 %), cerebral toxoplasmosis 22 (7.0 %), esophageal candidiasis 19 (6.0 %), cryptococcal meningitis (CM) 12 (3.8 %) and other 12 (3.8 %). TB was more frequent in IDUs compared to HSX and PI (43.3 % vs 31.0 % vs 22.9 % p < 0.0001), the latest developing more often cerebral toxoplasmosis (p = 0.0008) and PML (p < 0.0001). Patients diagnosed with CM had the lowest CD4 cell count/cmm at diagnosis (12, p = 0.005). PI patients were younger (median years) at OIs and HIV diagnosis compared to HSX and IDUs (25, 9 vs 38, 33 vs 30, 29 respectively, p < 0.0001). The overall mortality rate was high (27.0 %). IDUs had lower median survival time (months) compared to HSX and PI (10.5 vs 16.7 vs 17.8 respectively, p = 0.002).

**Conclusions**

The incidence of OIs during the study period was high, TB being predominant. OIs are still contributing to the increased HIV-related morbidity and mortality, due to late presentations and/or non-adherence to cART.

## POSTER PRESENTATIONS

### P1 Epidemiological aspects of co-infection of HIV/TB in Moldova

#### Iurie Osoianu, Ala Halacu

##### National Center of Public Health, Chișinău, Republic of Moldova

###### **Correspondence:** Iurie Osoianu (iurieosoianu@gmail.com) – National Center of Public Health, Chișinău, Republic of Moldova

**Purpose**

To assess and study epidemiological particularities of co-infection of HIV/TB and associated risk factors to provide appropriate recommendations directed to optimize the existing system of epidemiological surveillance and control of these infections.

**Objectives**

- To study epidemiological particularities of co-infection of HIV/TB in the Republic of Moldova associated with occupation, geographic area, sex, age, social and behavioral factors, and identify the prevalent factors;

- Identify risk factors associated with TB/HIV cases;

- Provide recommendations directed to improve surveillance systems of these infections.

**Materials and methods**

Cross-sectional study, conducted by interviewing 321 persons with coinfection of HIV/TB during 2013-2014.

**Results**

71.2 percent are men, mean age 37 years; 72 percent live in urban areas and 4.7 percent - do not have stable place of life; 85 percent do not work. 84.4 percent (±3.82) have a history or are present intravenous drug users. Only 2.6 percent (±4.98) are on opioid substituent treatment during TB treatment. 37 percent (±4.47) had experienced or are heavy drinkers (53 percent are undocumented on alcohol use). 34 percent (±5.38) experienced or are in current detention. 28.9 percent (±4.78) were out of the country before TB case notification. 56 percent (±5.31) have at least one co-morbidity; 33 percent of them have hepatitis C and 14 percent hepatitis B. No one received preventive treatment with isoniazid. There is a growing share of new registered HIV/TB cases in the Republic of Moldova in recent years.

**Conclusions**

Patients with TB/HIV are the most vulnerable to the higher proportion of people living with HIV and affected by social vulnerabilities and risk factors and require careful management of the case. Most of the cases were associated with drug use, few were opioid substituent treatment during TB and HIV treatment, which could increase adherence to TB treatment and ARVs. A significant proportion of patients are outside the viewfinder profile institution or occur in late stages of HIV disease. A significant proportion of patients have history of alcohol consumption and detention.

### P2 Perinatal exposure at HIV infection in Oltenia region

#### Andreea Cristina Stoian^1^, Florentina Dumitrescu^1^, Iulian Diaconescu^1^, Augustin Cupșa^1^, Lucian Giubelan^1^, Loredana Ionescu^2^, Irina Niculescu^1^

##### ^1^Department of Infectious Diseases, University of Medicine and Pharmacy from Craiova, Craiova, Romania; ^2^Clinical Hospital of Infectious Diseases from Craiova, Craiova, Romania

###### **Correspondence:** Andreea Cristina Stoian (andreea_plr@yahoo.com) – Department of Infectious Diseases, University of Medicine and Pharmacy from Craiova, Craiova, Romania

**Background**

Through the correct implementation of the preventive measures, the maternal transmission of HIV infection can be reduced to <2 %. The aim of the study is the epidemiological analysis of mother-to-child transmission of HIV in Oltenia region (Dolj, Olt, Mehedinți and Gorj counties).

**Methods**

Retrospective study in the Regional Centre for Monitoring and Evaluation of HIV/AIDS in Craiova, between 01/01/2004-31/12/2015 on a group of 131 children born of mothers HIV infected. There were analyzed: the moment of the mother’s HIV diagnosis, the mother’s immuno-virological status, birth method, antiretroviral (ARV) treatment and the modality of the child’s alimentation. The statistical analysis used Epi Info programme, the p value being statistically significant at <0.05.

**Results**

Demographics: the group of 131 children, F/M = 63 (48.09 %)/68 (51.91 %); U/R = 46 (35.12 %) /91 (64.88 %). Chronological distribution of the births showed a top of incidence in 2009 (19 births-14.51 %) and in 2007 (17 births-12.98 %), with an upward linear trend. The mother who was infected with HIV from the parenteral group in 1987-1990 period, was 105 (80.16 %), 24 mothers (18.33 %) were infected with HIV sexually, 1 (0.77 %) drugs consuming pregnant woman and 1 (0.77 %) pregnant woman, with unknown HIV transmission. Viral-immune evaluation of pregnant women (with 1-3 months before birth) showed average of lymphocytes CD4 = 443.147 ± 32.52 cells/cmm and an average of viremia HIV = 35051 copies/mL (4.54 log copies/mL). The method of birth was: natural birth in 26 cases (19.85 %) and 105 (80.16 %) mother through a cesarean. Antiretroviral treatment was gotten by 105 pregnant women (80.16 %), the most used schedule being: CBV + LPV/r = 84 (80 %). Artificial alimentation was administered to 117 children (89.32 %), the postpartum ARV prophylaxis was administrated to 120 children (91.61 %). During the survey period, 11 children (8.39 %) were diagnosed with HIV; 2 children (1.52 %) are still being evaluated. The characteristics of children HIV infected were: 5 children naturally born (p = 0.04), 3 children without ARV postpartum (p = 0.02), 6 children with artificial alimentation (p = 0.0010) and 7 mothers without ARV treatment (p = 0.002)

**Conclusions**

In Oltenia region there was registered a progressive trend, upwards for children born of HIV infected mothers, most of them coming from the parenteral infected cohort, with a high transmission rate, according to the lack of complete preventing measures of materno-fetal transmission.

### P3 Women living with HIV in Mureș county

#### Carmen Chiriac^1^, Nina Șincu^1^, Iringo Zaharia Kezdi^1^, Anca Georgescu^1^, Brândușa Țilea^1^, Cristina Girbovan^1^, Andrea Incze^1^, Andrea Fodor^2^

##### ^1^Department of Infectious Diseases, University of Medicine and Pharmacy Tîrgu-Mureș, Tîrgu-Mureș, Romania; ^2^Department of Laboratory Medicine, University of Medicine and Pharmacy Tîrgu-Mureș, Tîrgu-Mureș, Romania

###### **Correspondence:** Carmen Chiriac (carmen.l.chiriac@gmail.com) – Department of Infectious Diseases, University of Medicine and Pharmacy Tîrgu-Mureș, Romania

**Background**

Women infected with human immunodeficiency virus (HIV) represent one of the important phenomena in the present HIV pandemics. The natural hormonal, emotional and social changes represent a supplementary burden in HIV-infected women compared to men.

**Methods**

We performed a cross-sectional observational study regarding the women infected with HIV currently monitored in Mureș Regional HIV/AIDS Center. We collected data regarding the patients’ socio-demographic features, immunologic and virologic status, the impact of antiretroviral therapy, correlated with adherence, on the patients’ outcome and the risk of HIV transmission via vertical route.

**Results**

414 HIV-infected patients are monitored in Mureș Regional HIV/AIDS Center, 42.27 % of them women, out of which 94.28 % are of fertile age. As a peculiar feature of this group, 63.42 % of the female patients are long-term survivors, infected with HIV clade F1 during childhood. 85.14 % of all HIV-infected women and 84.24 % of patients of fertile age receive HAART. In female patients of fertile age, the median CD4+ T-cells count was 518 cells/μL, compared to a median of CD4+ T-lymphocytes of 519 cells/μL in men (p = 0.6633). 50.96 % HIV-infected female patients and 52.30 % men had undetectable HIV-RNA plasma viral load (p = 0.8378). In December 2015, 78 infants had been born to HIV-infected mothers from Mureș Regional Center during the past 10 years, 62 (87.17 %) of them born to mothers with long history of HIV infection. Mother-to-infant transmission of HIV infection occurred in 3 cases (3.84 %).

**Conclusions**

The long-term surviving female patients infected with HIV clade F have undergone major biological transformations in the natural evolution of women, from childhood to childbirth, due to effective antiretroviral therapy and monitoring. A new generation of HIV-negative children was born to HIV-infected mothers.

### P4 Late diagnosis of HIV infection in children - a challenge for Romania

#### Alina Cibea^1^, Mariana Mărdărescu^1^, Cristina Petre^1^, Ruxandra Drăghicenoiu^1^, Rodica Ungurianu^1^, Ana Maria Tudor^1,2^, Delia Vlad^1^, Carina Matei^1^

##### ^1^National Institute for Infectious Diseases “Prof. Dr. Matei Balș”, Bucharest, Romania; ^2^Carol Davila University of Medicine and Pharmacy, Bucharest, Romania

###### **Correspondence:** Alina Cibea (a_cibea@yahoo.com) – National Institute for Infectious Diseases “Prof. Dr. Matei Balș” Bucharest, Romania

**Background**

In the past few years, the percentage of mother-to-child transmission of HIV in Romania has remained under 4 %, similar to the percentage reported for the European Union. This is a proof that the National Program of Prevention and Control of HIV infection in Romania has really worked. However, there are still children who are diagnosed with HIV infection after their first months of life.

**Objective**

To assess some pediatric cases of late HIV diagnosis (after the neonatal period).

**Material and method**

Medical records of 13 children, aged 3 months to 9 years, hospitalized in the NIID “Prof. Dr. Matei Balș” in the period Jan 2013 - Oct 2015 have been analyzed. The evaluated parameters: age at diagnosis, HIV status in child’s family, associated HIV diseases, CDC stage of HIV infection at diagnosis, number of previous confinements, and evolution of patients with antiretroviral treatment.

**Results**

10 of the 13 children (77 %) were diagnosed with advanced HIV infection and 5 of these (50 %) were hospitalized for a period longer than 1 week on over 5 occasions before diagnosis. Among the associated diseases at diagnosis have been mostly opportunistic infections: tuberculosis, fungal infections and pneumocystosis.

**Conclusions**

Thanks to the prevention program of mother-to-child HIV transmission, Romania has a low percentage of perinatally acquired HIV infection. Nevertheless, children may still be diagnosed late, due to: lack of compliance with medical care during pregnancy - the pregnant woman and the new-born are not tested, lack of routine HIV testing for children with multiple confinements for various intercurrent illnesses.

### P5 Cirrhosis Assessment in Patients Co-infected HIV-Hepatitis B Virus

#### Elena Dumea^1,2^, Lucian Cristian Petcu^2^, Simona Claudia Cambrea^1,2^

##### ^1^Ovidius University, Constanța, Romania; ^2^Infectious Diseases Clinical Hospital, Constanța, Romania

###### **Correspondence:** Elena Dumea (elenadumea@yahoo.com) – Ovidius University, Constanța, Romania

**Background and aims**

In HIV-infected patients, due to a decrease in AIDS related-deaths, liver disease is now one of the most common causes of death. It is necessary to find cheap, accessible tools to assess the amount and progression of liver fibrosis and the risk for cirrhosis. We chose to use as non-invasive bio-markers for liver damage APRI and FIB-4 because these are easily calculated scores from the usual tests of laboratory, that can be performed periodically and could be a method of assessing longitudinal progression of liver disease in the absence of other methods invasive or more expensive or which would require special equipment as transient elastography (FibroScan).

**Methods**

We evaluated FIB-4 and APRI’s ability to differentiate between cirrhosis (F4 Metavir) and non-cirrhosis (F < 4) taking as a reference value Fibro-Scan. We used as cut-off Fibro-scan to differentiate cirrhosis F4 > 12.5kPa. The APRI score is calculated using the patient's aspartate aminotransferase (AST) level and platelet count (PLT), and the upper limit of normal of AST and FIB-4 results has generated utilizing age, AST, alanine aminotransferase and PLT. AUROC was used to calculate the optimal value for each score to Identify significant fibrosis. We studied 71 patients with a documented mean of HIV and hepatitis co-infection duration of 14.2 years, possible infected in early childhood.

**Results**

AUROC for APRI to identify cirrhosis was 0.942 95 % CI (0.860, 0.983), P <0.0001. Cut-off value for APRI in detection of cirrhosis in patients with HIV + HBV infection with maximum sensitivity and specificity was established > 1.49 (specificity 96.9 %).

AUROC for FIB-4 for identifying cirrhosis was 0.931 95 % CI (0.845, 0.977), P <0.0001. Cut-off value for detection of cirrhosis for FIB-4 in HIV + HBV patients with maximum sensitivity and specificity was > 1.3 (96.9 % specificity).

There is a difference between areas of 0.011 with a p 0.792. There is sufficient evidence that the tests used APRI and FIB-4 have the ability to distinguish cirrhosis in this co-infected patients (F0-3 versus F4).

**Conclusions**

The performance validation of these diagnostic tests was not established yet for this group of HIV + HBV patients. This study is trying to get the diagnostic criteria for liver disease in virus B co-infected HIV patients, using available methods, non-invasive, reproducible. Will be required large study trials to compare non-invasive methods with liver biopsy or to discover new diagnostic scores, individualized for these groups of patients.

### P6 HIV late presenters in Craiova Regional Center, Romania

#### Florentina Dumitrescu^1^, Augustin Cupsa^1^, Andreea Cristina Stoian^1^, Lucian Giubelan^1^, Irina Niculescu^1^, Iulian Diaconescu^1^, Dan Hurezeanu^1^, Livia Dragonu^1^, Mioara Cotulbea^2^

##### ^1^Infectious Diseases Department, University of Medicine and Pharmacy from Craiova, Craiova, Romania; ^2^“Dr. Victor Babeș” Hospital of Infectious Diseases and Pneumology, Craiova, Romania

###### **Correspondence:** Florentina Dumitrescu (elenadumea@yahoo.com) – Infectious Diseases Department, University of Medicine and Pharmacy from Craiova, Romania

**Background**

Late presentation for HIV care remains a significant issue. The aim of the study is to evaluate the proportion and the risk factors for late HIV testing and presentation to care in the Craiova Regional Center (CRC), Romania.

**Methods**

All patients newly HIV diagnosed in CRC between January 2010 and December 2014 were included an initial CD4 count <350 cells/cmm or an AIDS-defining illness within 6 months after diagnosis defined late presenters (LP). Demographic, behavioral characteristics and biological data of LP were compared with those of non-late presenters (NLP).

**Results**

Of 129 patients included, 79 (61.2 %) were LP. Characteristics of LP vs NLP: male-46 (58.2 %) vs 20 (40 %), urban area-35 (44.3 %) vs 27 (54.0 %), heterosexual acquired HIV infection- 71 (89.8 %) average vs 43 (86 %), average age at HIV diagnosis-30.7 ± 10.1 years vs 26.2 ± 14.8 years, median CD4- 124 (IQR 1, 335) cells/cmm vs 607 (IQR 359, 3605) cells/cmm. LP were more likely to be older (p = 0.04), male (p = 0.04), heterosexual (p = 0.01) and less likely to be highly educated (p = 0.0001). LP were more likely to have the first HIV test following a doctor recommendation (p = 0.001). NLP were tested for HIV after a specific risk situation (p < 0.0001).

**Conclusions**

In CRC Romania, the proportion of LP was high. Older age, male, heterosexual acquired of HIV and low level of education showed a higher risk of being diagnosed late. Better strategies for HIV testing are needed.

### P7 Some aspects of malignancies in patients HIV / AIDS

#### Simona Manuela Erscoiu^1^, Ionuț Cristian Popa^2^, Denisa Stroie^2^, Petronela Ionescu^2^, Nedeea Duță^2^, Camelia Dobrea^3^, Irina Voican^4^, Emanoil Ceaușu^1^, Petre Iacob Calistru^1^

##### ^1^Carol Davila University of General Medicine and Pharmacy Bucharest, Bucharest, Romania; ^2^Clinical Hospital for Infectious and Tropical Diseases “Dr. Victor Babeș” Bucharest, Bucharest, Romania; ^3^Monza Hospital, Bucharest, Romania; ^4^Bucharest University Emergency Hospital, Bucharest, Romania

###### **Correspondence:** Simona Manuela Erscoiu (serscoiu@yahoo.com) – Carol Davila University of General Medicine and Pharmacy Bucharest, Romania

**Background**

Although 20 years have passed since the initiation of HAART which has resulted in increased survival time and improved quality of life of patients infected with HIV, they are at higher risk of developing certain cancers such as Kaposi sarcoma (KS), non-Hodgkin lymphoma (NHL), and cervical cancer. Unfortunately, in recent years malignancies have increased in number to the entire population of the globe. The connection between HIV/AIDS and certain cancers is not completely understood, but the link likely depends on a weakened immune system. The incidence of KS and NHL has decreased markedly, but there has been a relative increase in tumor types that collectively are referred to as non-AIDS-defining cancers (NADCs) compared with the general population. NADCs now are a major factor contributing to mortality in HIV-infected people.

**Methods**

Retrospective, observational, descriptive study. Data were collected from January 1990 to December 2015 from patients admitted in Casa Andreea department. We included only patients in whom diagnostic criteria for malignancy were confirmed. If in the 90s, diagnosis and therapy were difficult, in last 5 years etiological and therapy possibilities have improved. Explored imaging cases, histological and immunohistochemistry examinations were done, the consequence being targeted chemotherapy.

**Results**

We analyzed 72 patients, 83 % being heterosexually HIV infected men. The median age was 39 years. 40 % of patients HIV had malignancies while only 20.34 % of them had been diagnosed with malignancy before HIV seropositivity. The rest had a range between 3 months and 10 years to malignancy diagnosis HIV viral load had a median of 570 000 c/mL and the CD4 cell count was below 200 lymphocytes /cmm at the cancer diagnosis (40.28 %). Immunohistochemistry was made in 42 % patients. Malignancy were NHL 28 %, KS 11 %, 10 % lung cancer, skin carcinoma 11 %, digestive cancer 11 %, 7 % cerebral lymphoma. At the time of diagnosis 58 % had already metastasized neoplasia. Radiotherapy received 24 %, 42 % had repeated courses of chemotherapy. HAART received 82 % of patients and at the end of the study 31.95 % had undetectable viral load. However the death toll was 71 %, 79 % died in the same year of malignancy diagnosis. Concomitant pulmonary TB had 34 % of patients.

**Conclusions**

As patients live longer with HIV, morbidity and mortality from cancers are increasing. We must be able to quantify and characterize cancers in HIV. Treatment of malignancies in HIV should be vigorous and appropriate to the situation.

### P8 Factors associated with resilience among people living with HIV in Romania

#### Florin Lazăr

##### Bucharest University, Bucharest, Romania

**Background**

Resilience, defined as the ability to adapt to new circumstances, is one of the major aims in the cascade of care of people living with HIV (PLHIV), presumably ensuring an independent, reliable partner during the management of the infection. The aim of the study is to identify factors associated with resilience of PLHIV in Romania.

**Methods**

A cross-sectional survey using a self-reported questionnaire was carried out between November 2014 and March 2015 among 252 adult PLHIV aware of their status for at least 6 months. A global score of resilience representing the dependent variable was derived from Resilience Scale for Adults (RSA, alpha = 0.943) created by Friborg et al. (2003, 2005) based on 33 pairs of situations/items with responses using semantic differential (five points) grouped into four subscales (personal strengths, family, relationships, social resources). Validated measures of quality of life (alpha = 0.934), coping (alpha = 0.843), depression (alpha = 0.946), HIV Stigma Scale (alpha = 0.943), were tested for association with RSA. Independent variables were subscales from the above-mentioned scales accompanied by variables on social support (no. of close relatives and friends to talk to), importance of God and adherence (no. of days of treatment interruptions in the last four weeks). A linear multivariate regression was performed (adjusted r square of the final model was: 0.841) to identify factors associated with resilience.

**Results**

The most important predictors of resilience are: not having lost interest in life (subscale from the depression scale) (ß CI95%: -0.499 [-7.228 - -4.140], p < 0.000), having a better quality of life, domain environment (ß CI95%: 0.338 [1.962- 4.845], p < 0.000), having more social support from family and friends (ß CI95%: 0.220 [0.365- 1.478], p < 0.002), having interrupted treatment less in the previous four weeks (ß CI95%: -0.207 [-3.145 - -0.752], p < 0.002) and considering God less important in life (ß CI95%: -0.184 [-2.686 - -0.528], p < 0.004).

**Conclusion**

PLHIV who are resilient have not given-up, receive more social support, live in a better environment (having a better quality of life) are more adherent to treatment, but do not rely on religion/God, being in control over their life. In building resilience in their patients professionals need to pay special attention to maintaining hope, enhancing social support, ensure access to a qualitative living environment and encourage PLHIV not to let their life in “God Will”, which in turn can have a positive impact on adherence.

### P9 Fever in HIV-infected patients: a thorny problem to be solved by the clinicians

#### Lucian Giubelan^1,2^, Augustin Cupșa^1,2^, Iulian Diaconescu^1,2^, Florentina Dumitrescu^1,2^, Dan Hurezeanu^1,2^, Livia Dragonu^1,2^, Irina Niculescu^1,2^, Andreea Cristina Stoian^1,2^, Oana Obretin^2^, Mariana Stănescu^2^, Mihai Jianu^2^

##### ^1^University of Medicine and Pharmacy, Craiova, Romania; ^2^Victor Babeș Hospital of Infectious Diseases and Pneumology, Craiova, Romania

###### **Correspondence:** Lucian Giubelan (ligiubelan@yahoo.com) – Victor Babeș Hospital of Infectious Diseases and Pneumology, Craiova, Romania

**Background**

Fever in HIV-infected patients poses many problems to the clinicians, forcing them to differentiate between the many possible causes (opportunistic infections, HIV itself, malignancies, various non-infectious causes).

**Case report**

We present the case of a male patient, 53 years old, from Craiova, admitted in our department in November 2015 for isolated fever (max. 40 °C, lasting for the last three weeks before admission). He did not consult his general practitioner, but he self-administered amoxicillin-clavulanate and non-steroidal anti-inflammatory drugs, 7 days before hospitalization. He was diagnosed with shingles 3 years ago; also he has lost 13 kilograms in weight in the last year. On physical examination he appeared well despite high fever, having generalised paleness, laterocervical lymph nodes enlargement, oral thrush and tachycardia (98/minute). Hematologic exploration revealed anemia (8.8 g/dL), normal leukocyte counts (5540/cmm) with lympho-monocytosis (43.7 %) and elevated erythrocyte sedimentation rate (115 mm/1 hour). Chest X-ray demonstrated a mediastinal widening and disseminated micro-opacities. A CT scan confirmed the presence of a tumoral mediastinal mass extending to the right thyroid lobe and strangulating the right subclavian artery. ELISA and Western blot tests for HIV were positive and further exploration showed at baseline a viral load of 574000 copies/mL and a CD4 count of 128 cells/cmm. He started antibacillary treatment (based on a presumptive diagnosis of tuberculosis) and, two weeks later, antiretroviral treatment with Abacavir, Lamivudine and Enfuvirtide. Under treatment fever resolved, but, two months later, reappeared after immune reconstitution (CD4 count 392 cells/cmm), imposing corticosteroid therapy. Currently, tests for diagnosis of malignancy are undergoing (non-Hodgkin lymphoma being suspected).

**Conclusion**

Fever in HIV-infected patients might be a thorny problem for the clinicians; based on epidemiological and statistical data, antibacillary treatment might be the starting point for solving this matter.

**Consent**

Written informed consent was obtained from the patient for publication of this Case report and any accompanying images. A copy of the written consent is available for review by the Editor of this journal.

### P10 Th1, Th2, Th9, Th17 and Th22 cytokines in acute and chronic HIV-1 infection

#### Lana Gorenec^1^, Snjezana Zidovec Lepej^1^, Ivana Grgic^1^, Ana Planinic^1^, Janja Iscic Bes^1^, Adriana Vince^2^, Josip Begovac^3^

##### ^1^Department of Molecular Diagnostics and Flow Cytometry, University Hospital for Infectious Diseases Zagreb, Zagreb, Croatia; ^2^Department of Viral Hepatitis University Hospital for Infectious Diseases Zagreb, and University of Zagreb, School of Medicine, Zagreb, Croatia; ^3^Department of HIV/AIDS, University Hospital for Infectious Diseases Zagreb, and University of Zagreb, School of Medicine, Zagreb, Croatia

###### **Correspondence:** Snjezana Zidovec Lepej (szidovec@gmail.com) – Department of Molecular Diagnostics and Flow Cytometry, University Hospital for Infectious Diseases Zagreb, Zagreb, Croatia

**Background**

The study is a retrospective analysis of cytokines expression on both gene and protein levels in HIV-infected individuals receiving clinical care at the Croatian Reference Center for HIV/AIDS of the University Hospital for Infectious Diseases (UHID) in Zagreb, Croatia.

**Methods**

Thirty four patients were enrolled for cytokine expression analysis on protein level in acute and chronic phase of HIV-1 infection. The expression of 84 cytokine genes was measured in 3 patients in acute and 3 patients in chronic phase of HIV-1 infection using PCR array technology. To measure the concentrations of Th1/Th2/Th9/Th17/Th22 cytokines, bead-based cytometry was applied.

**Results**

The results showed statistically significant increase of 13 cytokine gene expression (cd40lg, csf2, ifna5, il12b, il1b, il20, lta, osm, spp1, tgfa, tnfsf 11, 14 and 8) and downregulation of the il12a expression in chronic HIV-1 infection. Concentrations of IL-10, IL-4 and TNF-α were increased in the acute HIV-1 infection when compared to the control group. During chronic HIV-1 infection there was an increase of IL-10, TNF- α, IL-2, IL-6, IL-13 and IL-22 levels when compared to the control group. Comparison of cytokine expression between two stages of infection showed significant decrease in IL-9 concentration.

**Conclusion**

In this study we showed the existence of cytokine profile change on both gene and protein levels as the HIV-1 infection progresses from acute to chronic stage. There was no Th1 to Th2 cytokine switch between these two stages of infection. In chronic HIV-1 infection 13 genes encoding different cytokines were overexpressed indicating the hyperactivation of the immune system. Among HIV-infected individuals during chronic infection concentrations of IL-9 were significantly reduced when compared to the acute stage of infection. The hyperactivation of the immune system is present even during chronic HIV-1 infection with sustained viral replication.

Acknowledgments

This research was funded by the Croatian science foundation project titled “Molecular, epidemiological and clinical features of HIV infection in Croatia” (principal investigator Prof. Josip Begovac).

### P11 Dyslipidemia in HIV-infected patients treated with protease inhibitors – case report

#### Luminița Elena Horga

##### Infectious Diseases Hospital Cluj-Napoca, Cluj-Napoca, Romania

**Background**

People with HIV infection are getting older, thus they could have comorbidities associated with aged persons, like dyslipidemia. The treatment for the HIV infected patient who has also metabolic abnormalities is a challenge for all health care workers involved. Goals of therapy include promoting long-term adherence, avoiding drug interactions, minimizing toxic effects. The main objective of this presentation was to assess the impact of clinical pharmacist with significant issues related to the use of antiretroviral therapy (ART) and lipid lowering agents in patients with HIV infection and dyslipidemia.

**Case report**

We analyzed significant pharmacokinetic drug-drug interactions between statin and ART, the additive toxicities associated with concomitant ART and statin drug use.

A 47-year-old white male, light smoker, nondrug user, was confirmed with HIV infection stage B3 in 2013; his CD4 cell number was 2/cmm and presented elevated creatinine. He started antiretroviral treatment (ART) with abacavir, atazanavir and ritonavir, well tolerated. Clinical, immunological and virological evolution was very good. One year after starting ART triglyceride values reached 2322 mg / dL. Recommended by the nutritionist, fibrates (fenofibrate) and statins (rosuvastatin) regimen was introduced for lowering triglycerides. The pharmacist intervention was to reviewing the medication, screening for toxicity and drug-drug interactions, proper use of statin. Rosuvastatin increases the risk of myopathy and rhabdomyolysis associated with fenofibrates. If association is absolutely necessary, rosuvastatin daily dose should not exceed 10 mg. Concomitant administration of atazanavir/ritonavir and rosuvastatin may increase plasma concentrations of rosuvastatin- increasing the risk of rhabdomyolysis. If is required, it is recommended to administer the lowest concentrations of rosuvastatin. The dose of rosuvastatin was decreased from 20 to 10 mg. But in this situation the best regimen should be fenofibrate and fluvastatin because there are no interactions.

**Conclusions**

One of the duties of the clinical pharmacist is “to consult with the patient’s physicians and other health care providers in selecting the medication therapy that best meets the patient’s needs and contributes effectively to the overall therapy goals” (AACP).

**Consent**

Written informed consent was obtained from the patient for publication of this Case report and any accompanying images. A copy of the written consent is available for review by the Editor of this journal.

### P12 Why is less treatment used for the metabolic abnormalities in HIV patients-too many drugs?

#### Corina Itu^1^, Luminița Elena Horga^1^, Laura Augusta David-Aldea^1^, Anca Ciorogar^1^, Cristian Jianu^1^, Mihaela Lupșe^2^

##### ^1^Clinical Hospital for Infectious Diseases, Cluj-Napoca, Romania; ^2^Iuliu Hațieganu University of Medicine and Pharmacy, Cluj-Napoca, Romania

###### **Correspondence:** Corina Itu (corinaitu@yahoo.com) – Clinical Hospital for Infectious Diseases, Cluj-Napoca, Romania

**Background**

The etiology of dyslipidemia in HIV infected patients is multifactorial and its treatment is crucial in preventing cardiovascular disease. The life style changes are mandatory followed by medication. The lipid-lowering agents are similar to those used in general population, but polypharmacy (more than 5 daily), drug-drug interactions and adverse reactions are more frequent. Some studies showed that the use of lipid-lowering drugs remains lower in HIV infected adults than in the general population.

**Methods**

We conducted a cross sectional study during 2015 in HIV infected adults aged above 50, from The Regional AIDS Centre Cluj. The objective was to identify metabolic abnormalities, lipid-lowering treatment and associated medications. We analyzed the parameters: age, gender, comorbidities, lipid profile, CD4 cell number; also the number of concomitant HIV drugs, tuberculosis (TB) drugs and drugs for other comorbidities, patients in lipid-lowering treatment. Normal values for total cholesterol, LDL-cholesterol, triglycerides and total lipids were considered up to 220, 150, 200, 750 mg/dL.

**Results**

In the Cluj Regional Centre at the end of December 2015 were followed 446 patients; 370 were under antiretroviral therapy (ART). Above 50 years old there were 66 patients. We selected 60 patients, 59 with ART. Average age was 57 (54.7 for females). 19 were females, in menopause. The number of patients with comorbidities was: 16 gastrointestinal, 14 cardiovascular, 4 with active TB, 1 with diabetes. 17 of 60 were with intercurrences. 32 out of 60 (52 %) had dyslipidemia (12 women, 37.5 %): 11 with mixed dyslipidemia, 14 with hypercholesterolemia, 7 with hypertriglyceridemia. Average values for total cholesterol, LDL-cholesterol, triglycerides and total lipids were 250, 187, 303 and 923 mg/dL. Average CD4 cell count value was 503/cmm. Daily dose of medicines was 5.8 for HIV, TB and chronic comorbidities and 6.8 if adding the intercurrences. Only 4 patients (12 %) from 32 with dyslipidemia were on lipid-lowering drugs.

**Conclusion**

There is an important burden using many drugs in HIV infected adults above 50. Daily average of medicines is 5.8 and increases at 6.8 in case of intercurrences. The priority is represented by ART and drugs for acute comorbidities (like tuberculosis), drugs for chronic comorbidities and, unfortunately, least for dyslipidemia. There is a need for initiating treatment for dyslipidemia, if the life style is changed and dyslipidemia persists. Polypharmacy might be an obstacle in prescribing lipid-lowering medication.

### P13 Sacral Herpes Zoster, with hyperalgesic form, in a patient with C3 stage HIV infection

#### Iuliana Caramangiu^1^, Ovidiu Roșca^2^, Monica Cialma^2^, Andreea Ardeleanu^1^, Iosif Marincu^2^

##### ^1^Department of Infection Diseases I, “Victor Babeș” Clinical Hospital of Infectious Diseases Timișoara, Timișoara, Romania; ^2^Department of Infection Diseases I, University of Medicine and Pharmacy “Victor Babeș” Timișoara, Timișoara, Romania

###### **Correspondence:** Iuliana Caramangiu (balteanu.iuliana@yahoo.com) – Department of Infection Diseases I, “Victor Babeș” Clinical Hospital of Infectious Diseases Timișoara, Romania

**Background**

Patients with HIV/AIDS due to immunosuppression may develop repeated episodes of Herpes Zoster. We report a clinical case of HIV late presenter associated with sacral Herpes Zoster with a hyperalgesic form with favorable evolution.

**Case report**

The authors present the case of a patient of 35 years, known as a HIV late presenter, with mental retardation, esophageal candidiasis and gallstones, who was hospitalized in the Clinic of Infectious Diseases Timisoara for fever (T = 39 °C), maculo-papular-vezicular rash metameric (S1-S5) disposed, with burning sensation and sharp pain, sacral eschar, marked asthenia. The onset is acute two days before hospitalization with chills, malaise, painful skin rash with blisters involving the sacral area. The diagnosis was based on the clinical symptoms associated with biological samples (number of leukocytes, erythrocyte sediment ratio (ESR), C reactive protein (CRP), serum glucose, blood culture, CD4, viral load (VL), sacral wound secretion culture, etc.) and the results of paraclinical investigations (chest radiography). During hospitalization, the patient received treatment with: acyclovir 400 mg, 5x2 cp/day, sulfamethoxazole/trimethoprim, 2x2 cp/day, ceftriaxone 1 g, 2x2 g/day, fluconazole 200 mg, 1cp/day, Perfalgan 100 mL 1 fl/day when required, Arnetin f, 1 f/day, infusion solutions with NaCl 0.9 % 500 mL 1 fl/day, Ringer Solution 500 mL 1 fl/day, blood transfusion 1 U/day, plasma transfusion 1 U/day, non-steroids anti-inflammatory (NSAIDS), Algifen 1f/day if needed, etc.

We mention the following lab results: leukopenia (3330/μL), increased ESR (130 mm/1st hour), CRP = 82.04 mg/L, Hb = 7.9 g/dL, platelet (49000/ μL), sideremia (16 pg/dL), total protein (4.93 g/dL), CD4 = 15 cells/μL, VL = 457825 copies/mL; blood culture = the absence of microbial growth; urine culture, present *Escherichia coli* 100.000 CFU/mL; wound secretion culture present *Staphylococcus aureus* meticilino rezistent and *Acinetobacter baumannii*, with sensitivity to ceftriaxone, amikacin and sulfamethoxazole/trimethoprim. Under treatment with antibiotics, antivirals, analgesics, NSAIDs, infusion solutions and antipyretics, the eruption continued the evolutionary cycle, but the pain persisted throughout the affected dermatomes (S1-S5). It was established antiretroviral treatment with: TDF 1/day + FTC 1/day + RAL 2x1/day. There were no reported adverse drug reactions, and patient showed good adherence to treatment.

**Conclusions**

Severe immunosuppression during HIV infection in C3 stage may favor the occurrence of infections with Herpes Zoster. Clinical, biological and therapeutical monitoring are required for early diagnosis of opportunistic infections in patients with HIV/AIDS and the establishment of appropriate therapy.

**Consent**

Written informed consent was obtained from the patient for publication of this Case report and any accompanying images. A copy of the written consent is available for review by the Editor of this journal.

### P14 Factors associated with in-hospital mortality in tuberculous and cryptococcal meningitis

#### Raluca Jipa^1^, Eliza Manea^1^, Șerban Benea^1,2^, Irina Lăpădat^1^, Nicoleta Irimescu^1^, Irina Panait^1^, Cristian Niculae^1^, Adriana Hristea^1,2^

##### ^1^National Institute for Infectious Diseases “Prof. Dr. Matei Balș”, Bucharest, Romania; ^2^Carol Davila University of Medicine and Pharmacy, Bucharest, Romania

###### **Correspondence:** Raluca Jipa (ralucajipa@yahoo.com) – National Institute for Infectious Diseases “Prof. Dr. Matei Balș”, Bucharest, Romania

**Background**

*Mycobacterium tuberculosis* and *Cryptococcus* spp. are the most frequent etiologies of meningitis in HIV infected patients. Both tuberculous meningitis (TBM) and cryptococcal meningitis (CM) are associated with high risk of mortality, especially in HIV infected patients. We assessed the risk factors for in-hospital mortality in HIV infected patients with TBM and CM.

**Methods**

Retrospective study between January 2006 and December 2015. We included HIV infected patients diagnosed with TBM and CM. Hospital records were reviewed and data on patient history, epidemiological characteristics, clinical findings including neurological examination, cerebrospinal fluid (CSF) changes and cerebral imaging were analyzed. Patients were defined as having TBM according to a consensus definition published by Marais et al.; CM diagnosis was established upon positive India ink stain, culture and/or cryptococcal antigen assay. Neurological staging was made according to Medical Research Council definitions.

**Results**

We identified 41 HIV infected patients with TBM (26 definite, 12 probable and 3 possible) and 42 with CM. Fifteen (37 %) patients with TBM died compared to 17 (41 %) patients with CM. Nine patients (60 %) with definite TBM and 6 (40 %) with probable TBM died. No statistically significant differences were observed between patients with TBM and CM regarding demographic and clinical data except duration of symptoms before admission, 14 (9-15) days in patients with TBM vs. 4 (3-6) in patients with CM (p < 0.001) and CD4 cell count, 68 (20-145) cells/cmm in patients with TBM vs 13 (8-38) cells/cmm in patients with CM (p = 0.002). Most of the patients had severe neurological involvement (stages 2 and 3): 14 (93 %) patients with TBM vs 16 (94 %) patients with CM (p = 1, OR [95 % CI] = 1.1 [0.06-20]). A higher CSF protein level and number of cells per µL were observed in patients with TBM vs patients with CM, 230 (118-778) mg/dl in patients with TBM vs 58 (45-129) in patients with CM (p = 0.006), respectively 200 (9-1406) cells per µL in patients with TBM vs 60 (25-106) cells per µL in patients with CM (p = 0.013). Hydrocephalus was more frequent in patients with CM (41 %) than in patients with TBM (20 %) (p = 0.6, OR [95 % CI] =1.7[0.2-13.1]).

**Conclusion**

Patients diagnosed with TBM and CM were severely immunosuppressed and had a similar neurological involvement and high in-hospital mortality. Demographic and clinical data were similar in patients who died within the two groups. Patients with TBM who died had higher protein levels and numbers of cells per μL in the CSF vs those with CM.

### P15 Lipodystrophy: still present adverse event in resource-limited settings

#### Jovana Kusic^1^, Djordje Jevtovic^1^, Dubravka Salemovic^1^, Jovan Ranin^1^, Bozana Dimitrijevic^2^, Gordana Dragovic^2^

##### ^1^The HIV-AIDS Center, Institute of Infectious and Tropical Disease ‘Dr Kosta Todorovic’, School of Medicine, University of Belgrade, Belgrade, Serbia; ^2^Department for Pharmacology, Clinical Pharmacology and Toxicology, School of Medicine, University of Belgrade, Belgrade, Serbia

###### **Correspondence:** Jovana Kusic (jovanakusic@yahoo.com) – The HIV-AIDS Center, Institute of Infectious and Tropical Disease ‘Dr Kosta Todorovic’, School of Medicine, University of Belgrade, Belgrade, Serbia

**Background**

Highly active antiretroviral therapy (HAART) has led to dramatic reductions in mortality and morbidity of HIV-infected patients. Lipodystrophy, a syndrome with peripheral fat wasting and central obesity, is a well-documented side effect of old antiretroviral drugs, still prescribed as the first line regimen in resource-limited settings. The aim of this study was to evaluate the incidence of lipodystrophy and to determine its risk ratios in HIV-infected patients in a resource-limited setting.

**Methods**

This cross-sectional study included all the antiretroviral-naive HIV-infected patients commencing HAART from 1^st^ January 2001 to 31^st^ December 2013, at the HIV/AIDS Center, Institute of Infectious and Tropical Diseases, Belgrade, Serbia. Univariate and stepwise multivariate logistic regression analyses were used to determine the odds ratios (OR) with the confidence interval (CI) of 95 %, in order to establish the relative risk for development of lipodystrophy. The Kaplan-Meier-method was used to determine the probability of development lipodystrophy over time. All statistical analyses were performed using SPSS software version using 0.05 as a p-threshold for the significance.

**Results**

This study included 955 HIV/AIDS patients, 683 men and 272 women, followed for 8.6 ± 3.8 years. The prevalence of lipodystrophy was 64.9 %. Univariate and stepwise multivariate regression analysis identified that the female gender, hepatitis C virus co-infection, AIDS diagnosis prior to HAART initiation, nucleoside-reverse-transcriptase-inhibitors and protease-inhibitors based regimens had a high risk for developing lipodystrophy in HIV-infected patients (OR = 1.9, 95 % CI = 1.2-3.67, p = 0.04; OR = 3.97, 95 % CI = 1.1-7.1, p < 0.01; OR = 3.9, 95 % CI = 1.2-6.9, p < 0.01; OR = 2.9, 95 % CI = 1.4-3.8, p < 0.01; OR = 6.6, 95 % CI = 4.8-9.1, p < 0.01, respectively).

**Conclusion**

Despite much greater life expectancy of HIV-infected patients, treatment-related toxicities still remain a major concern. Lipodystrophy, as side effect of HAART, is particularly still important in resource-limited settings.

### P16 TB and HIV coinfected patient, an emergent challenge - case report

#### Laura-Augusta Aldea-David

##### Infectious Diseases Hospital, Cluj-Napoca, Romania

**Background**

Concomitant therapy for HIV and tuberculosis leads to polypharmacy. This fact might be a challenge for the physician, but also for the pharmacist. The objective of this presentation was to evaluate the impact of clinical pharmacists on important issues related to the use of antiretroviral therapy (ART) in patients with active tuberculosis disease.

We analyzed significant pharmacokinetic drug-drug interactions between antituberculous and antiretroviral treatment, the additive toxicities associated with concomitant ARV and TB drug use.

**Case report**

A 59-year-old white male nonsmoker, nondrug user, was confirmed with HIV advanced disease in January 2015. His CD4 was 9 cells/cmm. He was coinfected with disseminated tuberculosis, with lung, liver and spleen involvement. He was with bilateral stents and suprapubic drainage. Associated he presented tracheobronchitis with *Candida krusei* and urinary tract infection with *Klebsiella*, *Serratia* and *Enterococcus*. His medications include TB drugs, ART, antifungal and antibiotics. Patient tolerated drugs well, had immunological and virological response to ART. During the treatment his CD4 cell number increased to 104 cells/cmm, the viral load decreased and his clinical evolution was good. We reduced the polypharmacy by reviewing the medication, medication reconciliation, introduced treatment for opportunistic infections, screening for toxicity and drug interactions, proper use of rifampicin-isoniazid: rifampicin is a potent enzyme inducer of the cytochrome P-450 isozymes (CYP3A4, CYP1A2, CYP2A6, CYP2B6, CYP2C8/9, CYP2C19), leading to decreased serum concentrations of medications metabolized by that isoenzyme. Isoniazid is a potent inhibitor of the cytochrome P-450 isoenzymes (CYP2C9, CYP2C19, and CYP2E1) but has minimal effect on CYP3A, so the inductive effect of rifampicin outweighs the inhibitory effect of isoniazid. When used in conjunction with rifampicin, isoniazid dosage >10 mg/kg daily may increase the incidence of hepatotoxicity. The risk of hepatotoxicity is greater when rifampicin and isoniazid are given concomitantly than when either drug is given alone, so we delayed for 30 minutes the administration time between the two drugs; they must be taken on an empty stomach. We administrated Pyridoxine (vitamin B6) 25-50 mg daily to reduce the occurrence of isoniazid-induced side effects in the central and peripheral nervous system.

**Conclusions**

A high percentage of pharmacists’ recommendations were accepted by the physician, the majority of the pharmacist’s functions involved ART dosing, detection of drug interactions or adverse drug reactions, provision of drug information, ART adherence counseling. With the growing number of HIV-positive individuals worldwide, the clinical pharmacist may have a place in the multidisciplinary team, providing care in people life.

**Consent**

Written informed consent was obtained from the patient for publication of this Case report and any accompanying images. A copy of the written consent is available for review by the Editor of this journal.

### P17 Efficacy of prophylactic antiretroviral treatment in new-born infants from HIV-positive mothers in 2012-2014, for the North-Eastern part of Romania

#### Carmen Manciuc^1,2^, Cristina Nicolau^1^, Liviu Prisăcariu^1^, Alexandra Largu^1,2^

##### ^1^“Sf. Parascheva” Infectious Diseases Clinical Hospital, Iași, Romania; ^2^“Gr. T. Popa” University of Medicine and Pharmacy, Iași, Romania

###### **Correspondence:** Cristina Nicolau (crinic2004@yahoo.com) – “Sf. Parascheva” Infectious Diseases Clinical Hospital, Iași, Romania

**Background**

In the North-Eastern region of Romania most of the female HIV-positive population are sexually active and at child bearing age. In Romania there is a strict protocol regarding HIV vertical transmission. We aim to evaluate the efficiency of this protocol and the degree of appliance, reflected in the HIV status of new-born infants from HIV-positive mothers for a period of 3 years.

**Methods**

Of the 1424 patients actively monitored in the HIV/AIDS Regional Center in Iași, Romania, 46.5 % (663) are female. We evaluated retrospectively the files of all new-born infants from HIV-positive mothers for a period of 3 years (January 2012 - December 2014).

**Results**

In the period mentioned above, 100 children were born (36 in 2012, 38 in 2013, 26 in 2014); one death occurred 10 days after birth, due to multiple organ malformations; the lowest weight at birth was 750 g; 3 of the children (3 %) had a detectable viral load at birth; in one case we could not evaluate the viral load; 98 children (98 %) were born through caesarian section; 2 were born through natural labor (2 %), one of which at home. Mothers received treatment with lopinavir/ritonavir + zidovudine/lamivudine through the whole pregnancy in 81 cases; in 8 cases the mothers did not receive any treatment, being tested for HIV at birth. For all newborns prophylaxis was made with zidovudine + lamivudine for 6 weeks. Three children remained positive at 18 months, and therapy for them consisted in zidovudine + lamivudine + nevirapine.

**Conclusions**

Evaluation of pregnant HIV-positive women and prophylaxis for new-born infants in the evaluated period were conducted according to protocols, which resulted in a small percentage of HIV-positive children (3 %).

### P18 Surveillance of mother to child transmission of HIV in Romania – 31 December 2015

#### Mariana Mărdărescu^1,2^, Adrian Streinu-Cercel^1,2^, Cristina Petre^1^, Marieta Iancu^2^, Sanda Vintilă^2^, Daniela Vitelaru^2^, Iosif Ionel^3^, Claudiu Mihai Șchiopu^1^, Alexandra-Henriette Mărdărescu^1^

##### ^1^National Institute for Infectious Diseases “Prof. Dr. Matei Balș”, Bucharest, Romania; ^2^Compartment for Monitoring and Evaluation of HIV/AIDS Data in Romania, Bucharest, Romania; ^3^National Institute for Public Health, Romania-National Centre for Surveillance and Control of Transmissible Diseases, Bucharest, Romania

###### **Correspondence:** Mariana Mărdărescu (mardarescum@yahoo.com) – National Institute for Infectious Diseases “Prof. Dr. Matei Balș”, Bucharest, Romania

**Background**

The UNAIDS Report for 2015 revealed that out of a total of 36.9 million PLWHA (people living with HIV/AIDS) 17.4 million women were infected with HIV. The same statistics showed that out of approximately 5600 new cases of HIV infections daily, 600 were pediatric HIV new cases, with ages below 15 years, while around 5000 were adults over 15, 48 % of them being women. 73 % of pregnant women living with HIV/AIDS had access to antiretroviral treatment in 2014. In Romania statistics at 31 December 2015 for the period 1985-2015, showed that out of 13.766 PLWHA and registered in the national database, 5802 were women (48 %). Women at their fertile age (15-29 years) accounted for 3358 cases (42 %). At the same time, 211 (1.53 %) cases were reported in children through materno-fetal transmission (starting with 1990).

**Methods**

The National Registry of materno-fetal transmission of HIV collects and analyses data on mothers and their exposed infants from the nine Regional HIV Centers in Romania. For the mothers the focus falls on: time of HIV diagnosis, medical history, risk factors, disease and therapeutic history, immunological and virological investigations. Regarding their children, the assessment concentrates on risk factors such as type of birth, the newborn’s postpartum ART, type of feeding and ART during pregnancy. Furthermore, they undergo virological and immunological baseline investigations, and at 6 and 18 months of surveillance.

**Results**

During 01.01.2013-31.12.2015, we registered 568 mothers and children. 36 % of mothers belong to the 1987-1990 cohort, 35 % are cases with HIV sexual transmission who are already in surveillance and another 10 % are sexual and IDU (injecting drug use) transmission cases as well as late presenters in the sanitary system. From the overall female patients registered, 437 (77 %) were under ante- and intra-partum treatment and 460 (81 %) had a Cesarean section. Among infants with maternal exposure to HIV, 535 (94 %) received post-partum prophylaxis. 512 infants (90 %) underwent virological assay during the first 72 hours after birth. In terms of feeding, 540 (95 %) received formula. Early infant diagnosis revealed that 5 % of the assessed children were HIV infected.

**Conclusion**

Taking into consideration 4 essential risk factors that determine the status of infants to HIV positive mothers and by applying logistical regression we concluded that the mother’s treatment during pregnancy and the newborn’s postpartum ART represent risk factors with a significant impact on the infant’s status at birth (p value = 0.019 and p-value = 0.0001).

### P19 The antiretroviral therapy failure and the need to select the effective treatment in the Republic of Moldova

#### Pavel Micsanschi^1^, Tiberiu Holban^1^, Ina Bîstrițchi^1^, Lucia Pârțână^2^, Angela Nagîț^2^, Svetlana Popovici^2^, Maria Talmaci^2^, Irina Cucerova^2^

##### ^1^Department of Infectious Diseases, Parasitology and Tropical Medicine, Nicolae Testemițanu State Medical and Pharmacy University, Chișinău, Republic of Moldova; ^2^Hospital of Dermatology and Communicable Diseases, Chișinău, Republic of Moldova

###### **Correspondence:** Pavel Micsanschi (micsanschipavel@yahoo.com) – Department of Infectious Diseases, Parasitology and Tropical Medicine, Nicolae Testemițanu State Medical and Pharmacy University, Chișinău, Republic of Moldova

**Background**

We observed a continuous recording of new cases of first-line antiretroviral treatment failure that have led to the need to select the individual second-line antiretroviral therapy for the HIV-infected patients with therapeutic failure. The major facts which cause ARV failure are poor adherence, inadequate drug levels or prior existing drug resistance.

**Methods**

In this study we followed-up 45 patients who corresponded by immunological and clinical criteria for first line antiretroviral treatment failure (AZT + 3TC + EFV/ d4T + 3TC + NVP/ AZT + 3TC + NVP/ ABC + 3TC + EFV/ (TDF + FTC) + NVP/ ABC + 3TC + NVP/ (TDF + 3TC + EFV)/ (TDF + FTC) + EFV/ (TDF + 3TC) + NVP) and started the second-line therapy in 2011-2015. Included patients were aged between 23-69 years (38.4 ± 0.3 years). After first-line antiretroviral therapy failure (HIV RNA viral load measurement >400 copies/mL after 5 months) switching to a new second line regimen became necessary.

**Results**

All 45 patients who were confirmed with failure of first-line antiretroviral therapy started the second-line regimens of HAART. At the setting of first-line antiretroviral therapy failure approximately 2/3 of patients had viral load >100000 copies/mL. From the individuals included in the study who have been defined with the failing to the therapy, 38 patients were detected with CD4 counts <350 cells/cmm, out of which 25 patients were detected with CD4 counts <200 cells/cmm and 9 patients of them were detected with CD4 counts <50 cells/cmm that is associated with increased risk for morbidity or death. After 6 months from initiation of second-line antiretroviral treatment 41 patients had HIV RNA <25 copies/mL, three of them responded more difficult to treatment, but one patient had not responded to the therapy and maintained viral load >100000 copies/mL and was detected with CD4 counts <200 cells/cmm. The duration from initiation of first-line antiretroviral treatment until failure of it and initiation of second-line antiretroviral therapy was also significantly different for each patient but the median time was 4.13 years (interquartile range: 1-10 years).

**Conclusion**

Due to detecting early first-line treatment failure and initiation the second-line therapy in the majority of patients who had viral load >100000 copies/mL and the average CD4 cell count was 205.4 cells/cmm, antiretroviral treatment was successful. Our study outcomes support the need for early identification of first-line treatment failure because a delay in switching therapy in patients with high level of viral replication may lead to greater development of resistance and compromise the virological activity of second-line regimens.

### P20 Disseminated cryptococcosis in a patient with C3 HIV stage and multiresistant to antiretroviral therapy with lethal evolution

#### Sorina Georgiana Mitrescu^1^, Dana Mihalcea^1^, Iulia Caramangiu^1^, Ovidiu Roșca^2^, Iosif Marincu^2^

##### ^1^Department of Infection Diseases I, “Victor Babeș” Clinical Hospital of Infectious Diseases Timișoara, Timișoara, Romania; ^2^Department of Infection Diseases I, University of Medicine and Pharmacy “Victor Babeș” Timișoara, Timișoara, Romania

###### **Correspondence:** Sorina Georgiana Mitrescu (sorina.mitrescu@yahoo.ro) – Department of Infection Diseases I, “Victor Babeș” Clinical Hospital of Infectious Diseases Timișoara, Romania

**Background**

Cryptococcosis is a deadly opportunistic infection caused by an encapsulated yeast, *Cryptococcus neoformans*. The major predisposing factor is the profound cellular immune defect caused by HIV infection. Cryptococcal meningitis is the most common life-threatening fungal infection in patients with AIDS.

**Case report**

The authors present a clinical case of disseminated cryptococcosis in a patient with HIV infection stage C3 with multiple ARV therapeutic schemes from 2001 to 2014 and treatment failure. Clinical manifestations of infection associated with the diagnosis: lymphadenopathy and recurrent oropharyngeal candidiasis, anemia, lingering pneumonitis, right pleural effusion, and hilari adenopathy, recurrent diarrhea, wasting syndrome, tuberculous pleurisy, HIV encephalopathy seizure form, *Toxoplasma* chorioretinitis.

At first presentation in the clinic, the patient accuses fatigue, fever, polymorphic eruption, sweating, left hemibody motor deficit, tachycardia, cough, abdominal pains, hematuria and headache. The positive diagnosis of cryptococcal sepsis and cryptococcal meningitis was established based on following criteria: clinical (asthenia, fever and stiff neck), biological (ESR, WBC, CD4, viral load, CSF viral load, blood culture, CSF fungal culture), paraclinical (chest radiography, abdominal echography, cranial MRI).

We mention the following pathological results: ESR = 140 mm/h, CRP = 33.52 mg/L, WBC = 1000 cells/μL, CD4 = 5 cells/μL, viral load = 323867 copies/mL, CSF viral load = 54359 copies/mL, blood culture = *Cryptococcus neoformans*, CSF fungal culture = *Cryptococcus neoformans*. Paraclinical: chest radiography - right basal condensation block with free pleural effusion, cranial MRI - supra tentorial gliotic lesions probably of infectious origin. It was established medical therapy with fluconazole 1200 mg/day, antibiotics, vitamins, antiepileptic, balancing solutions. We changed the Combivir and Viramune treatment set from 2014, with Prezista 1200 mg/day, Norvir 200 mg/day, Intelence 400 mg/day and Isentress 800 mg/day. Under this treatment the patient had improved general state but he developed an anxious depressive syndrome and asked discharge against medical advice. Adherence to ARV treatment was below 70 % with virological and immunological failure despite more than 9 therapeutic regimens he had from 2001 to 2014.

**Conclusions**

Patients with advanced HIV infection, non-adherent and uncooperative to ARV therapy may develop severe forms of disseminated cryptococcosis with fatal outcome. Biological, therapeutical and clinical monitoring measures are required alonside individualized psychological counseling.

**Consent**

Written informed consent was obtained from the patient for publication of this Case report and any accompanying images. A copy of the written consent is available for review by the Editor of this journal.

### P21 Aspects of tuberculosis infection in HIV-positive patients from Romania – our experience

#### Anca Negru^1,2^, Daniela Munteanu^1^, Victoria Aramă^1,2^, Raluca Mihăilescu^1^, Ioan Diaconu^1^, Remulus Catana^1^, Cristina Popescu^1,2^, Alina Orfanu^1,2^, Anca Leuștean^1^, Mihaela Rădulescu^1,2^, Cătălin Tilișcan^1,3^, Raluca Năstase^1^, Violeta Molagic^1^, Irina Duport^1^, Cristina Dragomirescu^1^, Ștefan Sorin Aramă^3^

##### ^1^Adults 3 Department, National Institute for Infectious Diseases “Prof. Dr. Matei Balș”, Bucharest, Romania; ^2^Infectious Diseases Department, Carol Davila University of Medicine and Pharmacy, Bucharest, Romania; ^3^Physiopathology and Immunology Department, Carol Davila University of Medicine and Pharmacy, Bucharest, Romania

###### **Correspondence:** Anca Negru (anca1808@yahoo.com) – Adults 3 Department, National Institute for Infectious Diseases “Prof. Dr. Matei Balș”, Bucharest, Romania

**Background**

Tuberculosis (TB) incidence in HIV infected patients is increasing especially in areas with high TB endemicity like Romania. Furthermore, immunosuppression induced by the HIV infection changes the clinical presentation of TB, this group of patients presenting atypical signs and symptoms, the extrapulmonary TB being more frequent. Another aspect of TB infection is represented by the increase of multidrug resistant strains, leading to high morbidity and mortality.

**Methods**

We included into the study consecutive HIV infected patients diagnosed with tuberculosis in the Adults 3 Department of the National Institute for Infectious Diseases “Prof. Dr. Matei Balș”, from January until December 2015. TB diagnosis was established either through sputum smears microscopy, positive blood cultures or protein chain reaction (PCR) for *Mycobacterium tuberculosis* from pleural fluid, cerebrospinal fluid (CSF) or bronchial aspirate.

**Results**

We enrolled 13 patients with a median age of 33 (25; 38) years old, seven of which had history of intravenous drug use. Median number of CD4 cells at the time of TB diagnosis was 33 (15; 107) cells/cmm. In six cases both infections were diagnosed at the same time. Regarding the symptoms, the majority of patients had prolonged fever, without any respiratory symptoms, which persisted despite large spectrum antibiotic treatment. Also the chest X-ray was not conclusive for TB. Five patients had pulmonary TB, five had systemic infection without clinical or radiological signs of pulmonary involvement and three had both pulmonary and systemic TB. Out of those with systemic TB 5 patients had hepatic and splenic abscesses identified at ultrasound examination. In patients with systemic TB other sites of infection included pericardium, pleura, peritoneum, kidney, lymph nodes and central nervous system. In six cases the diagnosis was established through sputum smear microscopy while three patients had positive blood cultures. In the other cases the diagnosis was established using *Mycobacterium tuberculosis* PCR from other biological fluids such as pleural fluid, bronchial aspirate and CSF. We isolated 2 resistant strains: one resistant to rifampin and the other one to ethambutol and isoniazid. After initiation of anti-TB therapy two patients developed toxic hepatitis, in one case the patient being co-infected with hepatitis C virus. Two patients had died during hospitalization; none of them had resistant tuberculosis.

**Conclusion**

In HIV positive patients extrapulmonary TB is more frequent, this is why the patient may present atypical symptoms, so this diagnosis must always be taken into consideration because early treatment decreases morbidity and mortality rates.

### P22 Dyslipidemia in HIV-infected patients

#### Nicoleta M Negruț

##### Department of Neuroscience and Recovery, Faculty of Medicine and Pharmacy, University of Oradea, Oradea, Romania

**Background**

Dyslipidemia is a common metabolic comorbidity in HIV-infected patients and may increase the risk for cardiovascular disease. According to the World Health Organization ischemic heart disease will be the leading cause of death in all countries by 2030. HIV-infected patients have a high risk for cardiovascular disease due to toxicity from antiretroviral medications and uncontrolled HIV replication.

**Methods**

This retrospective study analyzed the lipid abnormalities mentioned in the clinical database of all HIV-infected patients admitted in Hospital “Dr. Gavril Curteanu” Oradea, Romania, from January to December 2015. Dyslipidemia was classified according to the European Atherosclerosis Society. The program IBM SPSS statistics version 22 was used for analysis of the dates.

**Results**

During 01.01-31.12.2015, a total of 42 files of HIV-infected patients, were available for analysis. In the study group the lipid abnormalities were found in 28 cases (66.67 %), hypercholesterolemia in 6 cases (21.43 %), hypertriglyceridemia in 6 cases (21.43 %) and mixed hyperlipidemia in 16 cases (54.14 %). We found positive correlation between the period of HIV infection and lipid values (Bravais-Pearson correlation coefficient r = 0.33, p = 0.02). No correlation was found between age of the patients and lipid values (Bravais-Pearson correlation coefficient r = 0.29, p = 0.058). Female patients developed hyperlipidemia in 11 cases and males in 17 cases (p = 0.49, chi-square test).

**Conclusion**

In our study, 66.67 % of HIV-infected patients had lipid abnormalities. Dyslipidemia was associated with HIV length infection, but not with gender and age of the patient.

Acknowledgement

Financing of this study was supported by own sources.

### P23 Challenges in the management of an HIV seropositive patient with psoriasis undergoing immunomodulator therapy

#### Violeta Elena Niță^1^, Daniela Ioana Munteanu^1^, Raluca Mihăilescu^1^, Ioan Diaconu^1^, Anca Negru^1,2^, Cristina Popescu^1,2^, Victoria Aramă^1,2^

##### ^1^National Institute for Infectious Disease “Prof. Dr. Matei Balș”, Bucharest, Romania; ^2^Carol Davila University of Medicine and Pharmacy, Bucharest, Romania

###### **Correspondence:** Violeta Elena Niță (violeta.nita@yahoo.com) – National Institute for Infectious Disease “Prof. Dr. Matei Balș”, Bucharest, Romania

**Background**

Psoriasis is recognized as a systemic autoimmune inflammatory disease and the association between HIV and psoriasis represents a challenge for the clinicians because both diseases have in the center of the pathophysiology the T-lymphocyte. Frequently the autoimmune disease (AI) is refractory to treatment and moreover the drugs themselves induce immunosuppression leading to increased morbidity and mortality. Our aim is to present the management difficulties in the setting of immunological failure in a patient with HIV and psoriasis.

**Case report**

A 34-year-old former intravenous drug user, with HCV chronic infection, psoriasis vulgaris and psoriatic arthritis was diagnosed in December 2014 with HIV infection stage B3 (CD4 cell count 93 cells/cmm) and started on combined antiretroviral therapy (cART)/DRV/r + 3TC + ABC. Subsequently he was admitted into the hospital multiple times due to worsening of the arthropathy. He was initiated on Sulfasalazine (SSZ) and a low dose of corticosteroid (Prednisone 30 mg). But the lack of improvement imposed the increase of cortisone dose (Medrol 32 mg) with slow tapering, and the replacement of ABC with AZT, the latter having better results on AI. When cortisone was about to be stopped the arthritis aggravated, requiring a prolonged period of therapy (6 months). In this context the patient’s immunological status deteriorated (CD4 cell count 20 cells/cmm, August 2015), even though the viral load was undetectable after 3 months of cART. The patient returned to the hospital with a right crural zoster infection accompanied by VZV meningitis, responsive to IV acyclovir. Next, the patient was diagnosed with left deep venous thrombosis which required sc anticoagulant therapy for 3 months. Because of drug-drug interactions (DDI) between protease inhibitor (PI) and acenocoumarol, PI was switched to an integrase inhibitor which induced toxic hepatitis. Therefore we resumed the PI and kept the sc anticoagulant therapy. One month later the patient was diagnosed with pulmonary TB (on microbiological and imaging grounds). He was started on standard antituberculous therapy and the PI was replaced with Efavirenz due to DDI. The patient’s AI deteriorated, needing multiple dermatological and rheumatologic consultations which recommended Methotrexate in place of SSZ and further reduction of Medrol dosages.

**Conclusions**

Both HIV and the treatment for AI lead to poor immune status rendering this sort of patients susceptible to opportunistic infections. ART should be selected cautiously in order to avoid DDI, and also the deterioration of the AI. These patients have complex unpredictable poly pathology, which brings supplementary challenges in their management.

**Consent**

Written informed consent was obtained from the patient for publication of this Case report and any accompanying images. A copy of the written consent is available for review by the Editor of this journal.

### P24 Acute peritonitis as a sign of IRIS in an HIV-infected patient with MAC latent infection

#### Alina Orfanu^1,2^, Cristina Popescu^1,2^, Anca Leuștean^1^, Anca Negru^1,2^, Remulus Catana^1^, Ioan Diaconu^1^, Cătălin Tilișcan^1,2^, Victoria Aramă^1,2^, Sorin Ștefan Aramă^2^

##### ^1^National Institute for Infectious Diseases “Prof. Dr. Matei Balș”, Bucharest, Romania; ^2^Carol Davila University of Medicine and Pharmacy, Bucharest, Romania

###### **Correspondence:** Alina Orfanu (alina.lobodan@yahoo.com) – National Institute for Infectious Diseases “Prof. Dr. Matei Balș”, Bucharest, Romania

**Background**

Immune reconstruction inflammatory syndrome (IRIS) is a severe, life-threatening complication in HIV-infected patients under antiretroviral therapy (ART). *Mycobacterium avium* complex (MAC) is a common infection in immunosuppressed individuals (CD4 count < 50 cells/cmm) which is often localized in the respiratory and gastro-intestinal tract. HIV-infected patients are at high risk of MAC reactivation that can be responsible for disseminated infection.

**Case presentation**

A 35-year-old patient was admitted to our clinic for prolonged febrile syndrome associated with weight loss, diarrhea for one month, extreme odynophagia and laterocervical lymphadenopathy. At the clinical exam we found oral candidiasis, wasting syndrome, generalized lymphadenopathy, hepatosplenomegaly. The laboratory tests confirmed stage-C3 HIV infection (CD4 count = 7 cells/cmm), with a viral load of 120000 copies/ml. ART was initiated with CBV + LPV/r, with a favorable outcome. Furthermore, *Pneumocystis jiroveci* pneumonia (PCP) prophylaxis with trimethoprim/sulfamethoxazole and MAC prophylaxis with clarithromycin were initiated. After two weeks, the patient developed high fever and severe abdominal pain, especially in the right flank and iliac fossae, associated with signs of peritoneal irritation. The laboratory tests indicated an important biologic inflammatory syndrome and a CD4 count = 214 cells/cmm (after only two weeks of ART). The patient needed emergency surgery that showed several necroses in the cecum and ascending colon wall, with secondary peritonitis. The cecum and the first part of the ascending colon were ablated and the histopathological examination of the operatory piece indicated granulomas suggestive for mycobacterial infection. In situ PCR for *Mycobacterium tuberculosis* and MAC was effectuated and it was positive for *Mycobacterium avium*. The final diagnosis was probably IRIS with digestive necrosis in an HIV-infected patient with the reactivation of a MAC latent infection. The patient received clarithromycin, ethambutol and ciprofloxacin for 6 months and continued the same ART regimen, with favorable outcome. The prophylaxis for PCP and MAC weren’t necessary anymore because the CD4 count was over 200 cells/cmm at discharge.

**Conclusions**

Every patient diagnosed with HIV should be rigorously monitored at the start of ARV, not only for the potential side effects, but for the risk of IRIS apparition. MAC etiology should be evocated in an HIV-infected patient who develops acute abdominal pain concomitant with a rapid immune reconstruction.

**Consent**

Written informed consent was obtained from the patient for publication of this Case report and any accompanying images. A copy of the written consent is available for review by the Editor of this journal.

### P25 The virologic outcome of the treatment of chronic hepatitis B among HIV co-infected patients on HAART

#### Ivana Pesic Pavlovia^1^, Dubravka Salemovic^2^, Jovan Ranin^2^, Djordje Jevtovic^2^

##### ^1^Department of Microbiology, Clinical Centre of Serbia, Belgrade, Serbia; ^2^Belgrade University School of Medicine Infectious & Tropical Diseases Hospital, Belgrade, Serbia

###### **Correspondence:** Ivana Pesic Pavlovia (ivanapesic69@yahoo.com) – Department of Microbiology, Clinical Centre of Serbia, Belgrade, Serbia

**Background**

Since chronic hepatitis B (CHB) is associated with considerable morbidity and mortality, in HIV co-infected patients, it should be properly treated. The main goal of antiviral therapy of CHB has been to maintain durable suppression of viral replication, precisely by achieving and maintaining undetectable viremia by a sensitive PCR assay. All HBV/HIV co-infected patients initiated lamivudine containing HAART, which was switched to a tenofovir (TDF) based one when it became available, and after HBV developed resistance to lamivudine.

**Patients and methods**

The study included all HBV/HIV co-infected patients who underwent lamivudine containing HAART between 2000 and 2015, and were switched to TDF based HAART, if experienced lamivudine failure. The HBV infection status was assessed using commercial immunoassays for different serological markers, such as HBsAg/Ab, HBeAg/Ab and HBcAb, according to the manufacturer’s protocol. To detect and quantify HBV DNA, real time PCR (Cobas TaqMan HBV Test version 2.0, Roche Molecular Systems, Branchburg, NJ, USA) was used. All HBV DNA values are reported in IU/mL. For the presentation of HBV-DNA levels logarithmic scale was used.

**Results**

After the mean duration of lamivudine containing HAART of 7.3 ± 3.2 years (range 1-15 years), lamivudine failure was recorded in 35/67 patients (52.2 %). Out of twenty-two remaining subjects with favorable virologic response to lamivudine, all achieved HBsAg loss, out of which 2 patients developed anti-HBs antibodies, after 4.1 ± 3.1 years (1-15 years), and 9 ± 2.8 years (7-15 years), respectively. After additional 2.1 ± 1.1 years of TDF containing HAART, hepatitis B viral load was 1.3 ± 1.1 log_10_ IU/mL HBV DNA. After TDF introduction, the probability of achieving optimal treatment response, which included either suppression of HBV DNA to less than 20 IU/mL, and/or HBsAg loss, was 20 %, 60 % and 90 % after additional 2, 3 and 5 years of TDF containing HAART, respectively. Taken together, among lamivudine, and/or TDF containing HAART treated patients with HBV/HIV co-infection the estimated median time to achieving optimal anti-HBV treatment response was 7 years. Since no HBV viral breakthrough occurred while on TDF, we consider that a prolonged treatment with TDF containing HAART is mandatory among those with suboptimal virologic suppression.

**Conclusion**

The benefit of tenofovir containing HAART among HBV/HIV co-infected patients, who previously failed HBV therapy with lamivudine containing HAART, was rather modest after approximately two years of treatment, while the estimated treatment success reached 90 % after 5 years suggesting that the prolonged TDF therapy is mandatory to achieve the favorable response.

### P26 A case of HIV encephalopathy with aphasia, agnosia, apraxia and right homonymous hemianopsia

#### Ovidiu Roșca^1^, Andreea Ardeleanu^2^, Iulia Caramangiu^2^, Daniela Desaga^1^, Valerica Bică^2^, Sorina Mitrescu^2^, Iosif Marincu^1^

##### ^1^Department of Infectious Diseases I, “Victor Babeș” University of Medicine and Pharmacy Timișoara, Timișoara, Romania; ^2^Department of Infectious Diseases, Clinical Hospital of Infectious Diseases and Pneumology “Victor Babeș”, Timișoara, Romania

###### **Correspondence:** Ovidiu Roșca (roscaovidiu2003@yahoo.com) – Department of Infectious Diseases I, “Victor Babeș” University of Medicine and Pharmacy Timișoara, Timișoara, Romania

**Background**

The central nervous system (CNS) plays an important role in human immunodeficiency virus (HIV) infection, being a major target of this virus. In the CNS, which acts as a unique compartment and as a sanctuary, HIV can replicate independently from the plasma being protected from the host immune system and antiretroviral therapy. The authors present a case of HIV encephalopathy with aphasia, agnosia, apraxia and right homonymous hemianopsia.

**Case report**

We report the case of a 30 year-old male patient admitted in our department in January 2011, for receptive and expressive language disorders progressively installed (mixed aphasia); subsequently occur: apraxia, agnosia for colors, right homonymous hemianopsia. The patient's symptoms started two months before admission; he was admitted to the neurology department where he tested HIV-positive. Based on clinical elements, biological samples (number of leukocytes, erythrocyte sedimentation ratio (ESR), C reactive protein (CRP), serum glucose, blood culture, CD4, viral load (VL), HIV-RNA from cerebrospinal fluid (CSF), etc.), and imagistic evaluation (cerebral magnetic resonance imaging - MRI) the diagnosis of HIV infection stage C3 and HIV encephalitis was established. Antiretroviral treatment was started with lopinavir/ritonavir 200/50 mg 4 tb/day and lamivudine/zidovudine 150/300 mg 2 tb/day. The clinical and biological evolution was initially favorable. The patient was admitted again in November 2011 presenting in addition to the first admission right hemiplegia, seizures, urinary incontinence.

Cerebral MRI revealed extensive encephalitis lesions. In January 2011: CD4: 164 cells/μL, HIV-RNA from blood: 210319 copies/mL, HIV-RNA from CSF: 59240 copies/mL. Neurological exam: conscious, alert (within aphasia), disoriented, possible walk, positive Romberg with slight oscillations, Babinski sign positive in the right, expressive and receptive language disorders, mixed aphasia, apraxia, agnosia for colors, right homonymous hemianopsia. In November 2011, after 10 months of antiretroviral therapy: CD4: 495 cells/μL, HIV-RNA from blood: undetectable, HIV-RNA from CSF: 9444 copies/mL. Despite administration of ARV with good CNS penetration, the evolution was unfavorable and the patient died.

**Conclusion**

Highly active antiretroviral therapy (HAART) is not enough to control HIV associated dementia (HAD), the proof being the lack of reversibility of dementia in the presence of HAART in some cases, like the appearance of neurocognitive disorders in patients with good cellular immunity. In our case, there was probably a compartmentalization of HIV-1 in CSF, leading to the emergence of a resistant virus.

**Consent**

Written informed consent was obtained from the patient for publication of this Case report and any accompanying images. A copy of the written consent is available for review by the Editor of this journal.

### P27 Molecular footprints on human immunodeficiency virus -1 genome and association with phylogenetic clustering among subtype B infected patients in Serbia

#### Marina Siljic^1^, Dubravka Salemovic^2^, Valentina Nikolic^1^, Djordje Jevtovic^2^, Ivana Pesic-Pavlovic^3^, Jovan Ranin^2^, Marija Todorovic ^1^, Maja Stanojevic^1^

##### ^1^Institute of Microbiology and Immunology, University of Belgrade Faculty of Medicine, Belgrade, Serbia; ^2^University Hospital for Infectious and Tropical Diseases CCS, HIV/AIDS Unit, Belgrade, Serbia; ^3^Microbiology Department, Clinical Center Serbia, Belgrade, Serbia

###### **Correspondence:** Marina Siljic (marinasiljic@gmail.com) – Institute of Microbiology and Immunology, University of Belgrade Faculty of Medicine, Belgrade, Serbia

The HIV evolutionary process is driven by complex interplay between viral and host factors. Within hosts, the viral evolution is strongly influenced by natural selection as a result of a continuous effort to evade the immune response. HIV successively fixes mutations that allow it to evade immune response, acquiring combinations of polymorphisms in viral genome termed molecular footprint. The most clearly defined footprints are those associated with HLA alleles that are linked to successful control of HIV infection. Additionally, other viral features may have important influences on viral evolution, pathogenesis and disease outcomes, often through indirectly disturbing replicative capacity of virus, including resistance mutations to antiretroviral drugs and other mutations in different HIV-1 genes. The impact of positive selective pressure on viral diversity inter-host, as opposed to spatial and temporal factors, is not equally clear.

We investigate the prevalence of different molecular footprints in HIV *pol* region among subtype B infected patients in Serbia and their persistence in HIV-1 transmission clusters. The presence of molecular footprints associated with HLA alleles considered to be protective (HLA B*57, HLA B*58, HLA B*13), as well as risk allele HLA B*35, transmission associated drug resistance mutations (TDR) and other mutations in viral *pol* sequences within the clades, will be explored.

Subtype B viral sequences obtained from patients at the Center for HIV/AIDS, Institute for Infectious and Tropical Diseases in Belgrade, in the period 2002-2015 will be included. Additionally we will include a number of viral sequences downloaded from Los Alamos database. Maximum likelihood phylogenetic trees will be constructed using Mega and Paup software under the nucleotide substitution model that will be chosen using JModelTest. Bootstrapped replicates of phylogenies will be sampled to assess support for clades. Bayesian methods will be performed for reconstruction of phylogenetic trees. Transmission clusters will be defined as those clades consisted of three or more sequences with bootstrap value higher than 90 %, Bayesian posterior probability higher than 0.9 and genetic distance among sequences in the clade less than 1.5 %. In order to maintain specificity in identifying true transmission clusters, we will perform Phylopart software. Mutations specifically associated with transmitted HIV-1 drug resistance will be analyzed with the Calibrated Population Resistance Tool (CPR) and Stanford Genotypic Resistance Interpretation Algorithm.

Revealing how viral evolution within hosts is translated into viral evolution inter hosts in one population is an important aspect in determining how immune escape polymorphism and drug resistance might spread.

Acknowledgement

This work was partially funded by the Ministry of Education and Science Republic of Serbia (grant No 175024).

### P28 Neurosyphilis and human immunodeficiency virus infection: double challenge

#### Nina-Ioana Șincu, Anca Georgescu, Brândușa Țilea, Iringo Zaharia Kezdi, Andrea Incze, Cristina Gârbovan, Carmen Lucia Chiriac

##### Department of Infectious Diseases, University of Medicine and Pharmacy Tîrgu-Mureș, Tîrgu-Mureș, Romania

###### **Correspondence:** Nina-Ioana Șincu (ninasincu@yahoo.com) – Department of Infectious Diseases, University of Medicine and Pharmacy Tîrgu-Mureș, Tîrgu-Mureș, Romania

**Background**

*Treponema pallidum* and human immunodeficiency virus share similar routes of transmission, as well as a predisposition for central nervous system impairment, with intertwining clinical symptoms. Co-infection represents a challenge, from the point of view of diagnosis, possible complications and complex therapy.

**Case report**

We present the case of a young, 46-year-old male patient, with risk factors for sexually transmitted diseases. The disease had an abrupt onset, with repeated episodes of transient right limbs deficit, interpreted by the neurologist as repeated transient ischemic attacks, with complete remission of symptoms between attacks.

Cerebral CT scan was negative, but cerebral angiography raised the suspicion of left anterior cerebral artery dissection. Since the patient had no obvious risk factors for cerebro-vascular disease, the neurologist considered a possible underlying infectious etiology. TPHA and VDRL were positive, and the neurosyphilis diagnosis lead to investigations regarding other sexually transmitted diseases. Anti-HIV antibodies were positive, which is when the patient was referred to the Clinic of Infectious Diseases. CD4+ T-lymphocytes count was 398 cells/μL. Positive TPHA and VDRL both from the patient’s serum and from his cerebro-spinal fluid confirmed the diagnosis of neurosyphilis. The patient received antibiotic treatment with iv penicillin G for neurosyphilis and started antiretroviral therapy: tenofovir + emtricitabine + darunavir + ritonavir. He tolerated therapy well. A cerebral angio-MRI scan was performed in order to clarify the suspicion of left anterior cerebral artery dissection, but the examination did not reveal any arterial impairment.

**Conclusions**

Both syphilis and HIV infection raise significant diagnostic issues regarding central nervous system impairment. Complex diagnosis and therapy of this co-infection requires the involvement of a multidisciplinary team. Central nervous system involvement can represent an inaugural manifestation in the course of HIV infection.

**Consent**

Written informed consent was obtained from the patient for publication of this Case report and any accompanying images. A copy of the written consent is available for review by the Editor of this journal.

### P29 Differences between HIV-infected adults since childhood and non HIV-infected persons on managing everyday life

#### Anca Elena Luca^1^, Florin Lazăr^2^, Adrian Luca^3^, Luminița Ene^1^, Roxana Rădoi^1^, Adina Talnariu^1^, Silvia Suciu^1^, Cristian Achim^4^

##### ^1^“Dr. Victor Babeș” Hospital for Infectious and Tropical Diseases, Bucharest, Romania; ^2^Faculty of Sociology and Social Work, University of Bucharest, Bucharest, Romania; ^3^Faculty of Psychology and Education Sciences, University of Bucharest, Bucharest, Romania; ^4^HIV Neurobehavioral Research Center, University of California, San Diego, USA

###### **Correspondence:** Anca Elena Luca (ancaluca@gmail.com) – “Dr. Victor Babeș” Hospital for Infectious and Tropical Diseases, Bucharest, Romania

**Background**

Children parenterally infected with HIV in the ‘90s are living for more than 25 years. The study aimed to investigate the long-term neurocognitive consequences of living with HIV compared to an age-matched control group.

**Methods**

HIV positive (HIV+) and negative (HIV-) young adults have undergone extensive neurocognitive testing (2012-2015) using a standardized battery assessing cognitive performance in 7 domains. Cognitive deficit was estimated by a composite global deficit score (GDS) > 0.5. Patients’ functioning was assessed using two self-report questionnaires: Activities of Daily Living (ADL, α = 0.69) which reports the competence in performing basic tasks of everyday life required for independent living such as housekeeping, shopping, cooking, managing finances etc. and Patient’s Assessment of Own Functioning Inventory (PAOFI α = 0.93): rated patients’ self-perceptions regarding the adequacy of their functioning in various everyday tasks and activities for items in five subscales (memory, language and communication, use of hands, sensory-perceptual, cognitive and intellectual functions) with answers ranging on a 6-point scale. Depressive symptoms were assessed with Beck Depression Inventory II (BDI α = 0.89). Analysis of variance (ANOVA) compared HIV+ with HIV- patients in terms of cognitive performance (GDS), depression (BDI), self-perceived functioning (PAOFI), cognitive complaints, level of education, parent’s education, use of academic abilities, different confounders (medical, psychiatric etc.) employment status and incomes.

**Results**

The study group included 233 HIV+ and 53 HIV- participants with median age of 24.3 years for both groups. HIV+ participants were less educated (F = 9.419, sig. = 0.002), less likely to be employed (F = 15.25, sig. = 0.000), more depressed (F = 4.572, sig. = 0.033), and had a higher GDS (F = 14.617, sig. = 0.000) than their HIV- counterparts. Based on ANOVA the effect size of employment status on depression (F = 5.970, sig. = 0.003) is significant and higher than the effect of HIV status (F = 4.712, sig. = 0.031). However, there were not statistically significant differences in terms of PAOFI, ADL, social interactions, various confounding factors (e.g. medical, psychiatric, language, educational), parents’ education or monthly incomes.

**Conclusion**

Overall young adults with chronic HIV infection had higher rates of neurocognitive impairment, more depressive symptoms, and were less likely to have a job than HIV uninfected matched controls. Having similar levels of self-perceived functioning and cognitive complaints suggests a coping mechanism developed by HIV+ participants to face the everyday challenges of living with the infection.

Acknowledgement

NIH-funded grant R01MH094159-01 “Long term effects of chronic HIV infection on the developing brain”.

### P30 Molecular detection of *Bartonella quintana* in a HIV immunodepressed patient with fever and isolated lymphadenopathy - Case report

#### Diana Gabriela Iacob^1^, Dragoș Florea^1,2^, Simona Iacob^1,2^

##### ^1^National Institute for Infectious Diseases “Prof. Dr. Matei Balș”, Bucharest, Romania; ^2^Carol Davila University of Medicine and Pharmacy, Bucharest, Romania

###### **Correspondence:** Diana Gabriela Iacob (dianagiacob@gmail.com) – National Institute for Infectious Diseases “Prof. Dr. Matei Balș”, Bucharest, Romania

**Background**

*Bartonella quintana* is an opportunistic pathogen with a wide array of presentations, including isolated lymphadenopathies. We report a case of *Bartonella quintana* detected using a PCR assay in a HIV-positive patient with axillary lymphadenopathy in which the gold standard culture tests were unavailable.

**Case report**

We describe the case of a 31 year-old patient with HIV-HCV coinfection, previously diagnosed and treated for tuberculous lymphadenopathy who presented for fever, headaches, weight loss and painful axillary lymphadenopathy for the past 6 weeks. The patient was non-adherent to the antiretroviral treatment and reported close contact with cats. Clinical examination revealed an isolated, well defined left axillary lymphadenopathy of 5/6 cm, tender and mobile, with no overlying inflammatory signs. Laboratory assays revealed a severe immunosuppression (CD4 = 11 cells/cmm) and a moderate inflammatory syndrome, with negative procalcitonin. Blood culture and PCR for *Mycobacterium tuberculosis* (Cepheid GeneXpert MTB/RIF) were negative, as were the serologic analysis for *Toxoplasma* and syphilis. Nevertheless a blood sample sent for PCR coupled with mass spectrometry retrieved *Bartonella quintana*. Other imaging studies did not reveal any other mediastinal, retroperitoneal or mesenteric lymphadenopathies, cerebral MRI showed cortical atrophy and cerebrospinal fluid studies were normal. Unfortunately, the patient refused lymph node biopsy and we could not rule out a co-infection with *Mycobacterium tuberculosis* in the setting of an incomplete antituberculosis therapy. He underwent a successful 2 week-course of ciprofloxacin (the patient was allergic to clarithromycin and cotrimoxazole) along with the anti-tuberculous treatment with izoniazid/rifampicin/streptomycin and antiretroviral treatment.

Conclusions

The case raises the awareness for the importance of molecular diagnostic assays and also highlights the possibility for *Bartonella* infections in previous tuberculous lymphadenopathies.

Consent

Written informed consent was obtained from the patient for publication of this Case report and any accompanying images. A copy of the written consent is available for review by the Editor of this journal.

### P31 Present epidemiological characteristics of HIV/AIDS newly diagnosed cases in South-Eastern Romania

#### Manuela Arbune^1,2^, Miruna Drăgănescu^1,2^, Alina Iancu^2,3^

##### ^1^Clinical Department, “Dunărea de Jos” University Galați, Galați, Romania; ^2^Infectious Diseases Clinical Hospital Galați, Galați, Romania; ^3^Morphologic and Functional Sciences Department, “Dunărea de Jos” University Galați, Galați, Romania

###### **Correspondence:** Manuela Arbune (manuela.arbune@ugal.ro) – Clinical Department, “Dunărea de Jos” University Galați, Galați, Romania

**Background**

Patients from the Romanian HIV/AIDS pediatric cohort have grown up to the current age 25-29 years old. They signify 53.3 % from 328 beneficiaries of HIV Clinic Galați. New HIV/AIDS cases with various transmission ways are continuing to be diagnosed year by year. A previous evaluation (2005 - 2010) in Galați reported 73 % HIV late presenters (LCD4 < 350/μL). The social, economic, and political changes that occurred in Europe have impact on HIV epidemic.

**Methods**

We have retrospectively evaluated the new HIV diagnosed cases in Galați Clinic, from 2011 to 2015. The analysis of the HIV case report form included demographic data, risk factors of virus transmission, baseline immunity, co-morbidities and the antiretroviral treatment. The data were compared with situation 2005-2010. Statistical analysis performed descriptive and correlation tests, with significance level <0.005.

**Results**

An average of 20.2 new HIV cases were diagnosed yearly. Three children under age 13 were perinatally HIV infected. The average age in adults was 33.1 ± 12.3 years. Among 106 patients, 61.7 % were males, 56.3 % lived in urban area, 13.5 % had low education, 51.4 % were married/couples, 62.2 % travelled or worked abroad more than 6 months, especially in Italy (38.8 %). Positive HIV tests in other countries were found in 31 % adults. Symptomatic diseases were indicative for HIV testing in 21.3 %. The main reasons for volunteer HIV testing were the screening programs for sexually transmitted diseases (23.3 %), tuberculosis (10.68 %), blood donors (15.5 %), pregnancy (45.6 % of fertile women) and circumstances of a HIV positive partner (36.8 %). Frequency of the viral hepatitis was 21.3 % for HBV and 8.5 % for HCV. Baseline LCD4 was 379 ± 336.7/μL, corresponding to 56.3 % late presenters and 23.3 % very late presenters. Antiretroviral treatment was initiated in 63.2 % patients, mostly with protease inhibitors. The retention in care was 91.3 %, while 9 patients were lost from evidence. Mortality was 14.6 %. The risk of death was correlated with older age, single status, work abroad, lower immunity and tuberculosis as “sentinel” disease.

**Conclusions**

Mortality in patients newly diagnosed with HIV infection tends to increase, although late presentation and the retention in care were improved in the last years. Travel and work migration are run on risks for HIV transmission and poor prognostic. The current strategy for better control of HIV infection, is earlier time diagnostic, continuing the screening programs and encouraging the volunteer testing.

### P32 The gender’s preferences among opportunists?

#### Ruxandra Moroti^1,2^, Cristian M Niculae^1^, Simona Merisor^1^, Eliza Manea^1^, Serban Benea^1,2^, Andrada Stan^1^, Raluca Hrisca^1^, Raluca Jipa^1,2^, Diana Tanase^1^, Adriana Hristea^1,2^

##### ^1^Clinical Department, National Institute for Infectious Diseases “Prof. Dr. Matei Balș”, Bucharest, Romania; ^2^Carol Davila University of Medicine and Pharmacy, Bucharest, Romania

###### **Correspondence:** Ruxandra Moroti (ruxandra_moroti@yahoo.com) – Clinical Department, National Institute for Infectious Diseases “Prof. Dr. Matei Balș”, Bucharest, Romania

**Background**

We are living in symbiosis with a large variety of viruses, bacteria, fungi and parasites. Some of them act like opportunists when the immune defense gets weak. Part of our immunological reactivity is gender-related and we can speculate that there could be a gender-linked permissiveness for specific microbes or, vice-versa, specific germs have predilection for males or for females. Our objective was to explore a group of patients with very low immunological status, in order to find if there is a gender affinity of the opportunistic agents implied in their infections.

**Method**

Our retrospective study analyzed all HIV-positive adult patients (>18 years-old) admitted in one year interval (01 January-31 December 2015) in the National Institute for Infectious Diseases with a newly diagnosed invasive opportunistic infection (OI): mycobacteriosis - tuberculosis (TB) and atypical mycobacteriosis, *P. jirovecii* pneumonia (PJP), cryptococcosis, toxoplasmosis, cytomegalovirus (CMV) infections, Kaposi sarcoma (KS), progressive multifocal leukoencephalitis (PML). Three parameters were further analyzed: sex, age and the patient’s outcome.

**Results**

There were 118 HIV-positive patients admitted with at least one OI, 76 male patients (64 %) and 42 female patients (36 %), with a sex distribution of 1.8:1. The median age of the entire group was 33 [26-40] years old. There is significant difference between median age in men (34) and in women (32), p = .028. The outcome was fatal in 13 patients (11.01 %), with a mortality rate being double in male (13.1 %) versus female (7.1 %), p = 0.3. There was TB predominance among IO, consisting in 63 cases (53.38 %), 2:1 male:female ratio, with a fatal course in 4 cases: 3:1 male:female. PJP was diagnosed as IO in 20 cases, 3:2 male:female, with one death. For toxoplasmosis (10 cases), the sex-ratio was 2:3 male:female, with one death. PML was an IO in 10 cases, 4:6 male:female, with a fatal outcome in 3 male patients. Kaposi was diagnosed in 10 cases, 4:1 male:female and 2 cases were fatal (2 males). PML was IO in 10 cases, 1:1, with 3 fatal cases in males. In 7 cases, CMV was implied (4:3 male:female). Cryptococcosis was involved in 5 cases, all males, with 4 deaths.

**Conclusions**

OI roughly predominated in males in our study. Within specific OI categories, the sex-ratio seems to be different, the male predominance being perceptible for *Cryptococcus*, MTB and Kaposi and a female predominance for *Toxoplasma* and polyomaviruses.

### P33 Polymorphism of interleukin-28B gene in persons with chronic hepatitis C from Croatia

#### Ivana Grgic^1^, Ana Planinic^1^, Lana Gorenec^1^, Snjezana Zidovec Lepej^1^, Adriana Vince^2^

##### ^1^Department for Molecular Diagnostic and Flow Cytometry, University Hospital for Infectious Diseases Zagreb, Zagreb, Croatia; ^2^Department of Viral Hepatitis, University Hospital for Infectious Diseases Zagreb, Zagreb, Croatia

###### **Correspondence:** Ivana Grgic (ivanahobby@gmail.com) – Department for Molecular Diagnostic and Flow Cytometry, University Hospital for Infectious Diseases Zagreb, Zagreb, Croatia

**Background** Polymorphism of the promoter region in the gene coding for interleukin-28B (IL-28B) is associated with the natural history of HCV infection and response to antiviral and immunomodulatory therapy. Despite the fact that the predictive role of IL-28B genotyping is decreasing in the era of highly successfully direct acting antiviral (DAA)-based treatment, it remains to be useful in resource-limited settings with limited access to new treatment options.

**Methods**

The study included 380 Caucasian patients with chronic hepatitis C receiving clinical care at the Department of Viral Hepatitis of the University Hospital for Infectious Diseases, Zagreb and Croatian Reference Center for Viral Hepatitis. Human DNA was extracted from whole blood by using Qiagen Blood Mini kit. Detection of single nucleotide polymorphism (SNP) rs12979860 was determined by using a real-time PCR test IL28B LightMix® Kit IL28B rs12979860 (TIB Molbiol, Berlin, Germany) and LightCycler® FastStart DNA Master HybProbe (Roche Diagnostic, Germany).

**Results**

Analysis of rs12979860 SNP in the IL-28B gene revealed 222 of 380 (58.4 %) CT heterozygotes, followed by C homozygotes (102 of 380 patients, 26.8 %) and T homozygous patients were the least frequent (56 of 380 patients, 14.7 %).

**Conclusion**

The patterns of IL-28 polymorphism in Croatian patients with chronic hepatitis C is in accordance with other results for Caucasians.

